# Supplementary material for: Can Schwartz Center Rounds support healthcare staff with emotional challenges at work, and how do they compare with other interventions aimed at providing similar support? A systematic review and scoping reviews
Source: BMJ Open. 2018 Oct 18;8(10):e024254. doi: 10.1136/bmjopen-2018-024254 (PMC6196967; doi:10.1136/bmjopen-2018-024254)
Supplement: Supplementary file 1 [file bmjopen-2018-024254supp001.pdf]

Supplementary file 1: Composite definition of Rounds (key features shown in bold)

"Schwartz Rounds are the signature programme of the Schwartz Center for Compassionate Care. They provide a **regular** (usually monthly) **open forum** (drop-in rather than by invitation) for **multidisciplinary** clinical and non-clinical **staff at all positions** within the healthcare organisation to come together in an environment that is intended to provide a **safe and confidential space**. They are intended to provide staff with a **level playing field** to **reflect** upon, explore and tell stories about the difficult, **challenging and rewarding experiences they face when delivering patient care**, and **receive the support of their colleagues**. Rounds are typically organised and managed by a steering group, **championed by a senior doctor/clinician**. They **last for one hour** and are often held during lunch periods (with **food provided**). They are a **group intervention** within which **multiple perspectives on a theme, scenario or patient case** (i.e. their stories) are briefly presented by a **pre-arranged and pre-prepared panel** and then opened to the audience for group reflection and discussion, usually **facilitated** by a senior doctor and psychosocial practitioner. The **focus is on the non-clinical aspects (e.g. psychosocial, ethical and emotional issues) surrounding the patient-caregiver relationship** – thereby addressing a wide range of important topics rarely discussed elsewhere - and the attendees are encouraged to be **open and honest** and reflect, discuss and explore their experiences thoughts and feelings. The interaction between the panellists and audience is felt to foster insight and support from colleagues, create a sense of working in a supportive environment and lead to improved relationships and communication within the hospital hierarchy, improved communication and teamwork between staff and patients and among staff, improved wellbeing of staff including enhanced resilience, improved compassionate care and ultimately impact on organisational culture."

Some sources explicitly articulated what Rounds were ***not***. This included: that aims were distinct from traditional clinical/grand rounds; that is stories and discussions should **not focus**

on the patient, their diagnosis or plan of care; that Rounds should not be used for **problem solving**; and that they are **not intended to produce actionable outputs**.
