# Supplementary material for: Can Schwartz Center Rounds support healthcare staff with emotional challenges at work, and how do they compare with other interventions aimed at providing similar support? A systematic review and scoping reviews
Source: BMJ Open. 2018 Oct 18;8(10):e024254. doi: 10.1136/bmjopen-2018-024254 (PMC6196967; doi:10.1136/bmjopen-2018-024254)
Supplement: Supplementary file 2 [file bmjopen-2018-024254supp002.pdf]

Supplementary file 2: References for included papers from the scoping reviews of comparable interventions

Action Learning Sets

Bailie L, Bromley B, Walker M, Jones R, Mhlanga F (2014) Implementing service improvement projects within pre-registration nursing education: A multi-method case study evaluation. *Nurse Education in Practice*, 14(1): 62-68.

Booth A, Sutton A, Falzon L (2003) Working together: supporting projects through action Learning, *Health Information and Libraries Journal*, 20: **2**25–231.

Bourner T & Frost P (1996) Experiencing action learning. *Employee Counselling Today*, 8(6): 11–18.

Burgess H & Carpenter J (2008) Building capacity and capability for evaluating the outcomes of social work education (the OSWE project): Creating a culture change. *Social Work Education*, 27(8): 898-912.

Currie K, Biggam J, Palmer J, Corcoran T (2012) Participants' engagement with and reactions to the use of on-line action learning sets to support advanced nursing role development. *Nurse Education Today* 32: 267–272.

Douglas S & Machin T (2004) A model for setting up interdisciplinary collaborative working in groups: lessons from an experience of action learning. *Journal of psychiatric and mental health nursing*, 11: 189–193.

Eisen S, Sukhani S, Brightwell,A, Stoneham S, Long A (2013) Peer mentoring: evaluation of a novel programme in paediatrics, *Arch. Dis. Child.* 0:1–5.

Lamont S, Brunero S, Russell R (2010) An exploratory evaluation of an action learning set within a mental health service. *Nurse Education in Practice*, 10: 298-302.

Leggat S, Balding C and Anderson J. (2011) Empowering health-care managers in Australia: an action learning approach, *Health Services Management Research*, 24: 196–202.

Machin A & Pearson P (2014) Action learning sets in a nursing and midwifery practice learning context: A realistic evaluation. *Nurse Education in Practice* 14: 410-416.

Mann R, Ball K, Watson G (2011) Mentoring for NHS general practitioners: a prospective pilot study of an action learning approach, *Educ Prim Care,* 22(4): 235-240.

Moore L (2007) Partnerships and work-based learning: an evaluation of an opportunity to pioneer new ways to care for the older people in the community, *Assessment & Evaluation in Higher Education*, 32(1): 61-77.

Phillips S, Tapping J, Ooms A, Marks-Maran D, Godden R (2013) A preceptorship programme for health visitors and school nurses: a pilot study *Community Practitioner*, 86(1): 18-22.

Rivas K & Murray S (2010) Our shared experience of implementing action learning sets in an acute clinical nursing setting: approach taken and lessons learned. *Contemporary Nurse*, 35 (2): 182-7.

After Action Reviews

Reiter-Palmon R, Kennel V, Allen JA, Jones KJ, Skinner AM (2015) Naturalistic decision making in after-action review meetings: The implementation of and learning from post-fall huddles *Journal of Occupational and Organizational Psychology*, 88(2): 322–340.

Walker J, Andrews S, Grewcock D, Halligan A (2012) Life in the slow lane: making hospitals safer, slowly but surely. *Journal of the Royal Society of Medicine*, 105: 283–287.

Balint Groups

Abeni MS, Magni M, Conte S, Mangiacavalli L, Pochintesta G, Vicenzi VV, Ferretti A, Pompa F, Cocito C, Klersy, Corso C (2014) Psychological care of caregivers, nurses and physicians: A study of a new approach. *Cancer Medicine* 3(1): 101-110.

Adams KE, O'Reilly M, Romm J, James K (2006) Effect of Balint training on resident professionalism. *American Journal of Obstetrics & Gynecology* 195(5): 1431-1437.

Airagnes GSM, Consoli O, De Morlhon AM, Galliot C, Lemogne, Jaury P (2014) Appropriate training based on Balint groups can improve the empathic abilities of medical students: A preliminary study. *Journal of psychosomatic research* 76(5): 426-429.

Dahlgren MA, Almquist A, Krook J (2000) Physiotherapists in Balint group training. *Physiotherapy Research International* 5(2): 85-95.

Dokter HJ, Duivenvoorden HJ, Verhage HF (1986) Changes in the attitude of general practitioners as a result of participation in a Balint group. *Family practice* 3(3): 155-163.

Fitzgerald G & Hunter MD (2003) Organising and evaluating a Balint group for trainees in psychiatry. *Psychiatric Bulletin* 27(11): 434-436.

Johnson AH, Brock CD, Hamadeh G, Stock R (2001) The current status of Balint groups in US family practice residencies: A 10-year follow-up study, 1990-2000. *Family medicine* 33(9): 672-677.

Johnson AH, Brock CD, Hueston WJ (2003) Resident physicians who continue Balint training: a longitudinal study 1982-1999. *Family medicine* 35(6): 428-433.

Johnson AH, Nease DE, Milberg LC, Addison RB (2004) Essential characteristics of effective Balint group leadership. *Family medicine* 36(4): 253-259.

Kjeldmand D & Holmstrom I (2008) Balint groups as a means to increase job satisfaction and prevent burnout among general practitioners. *Annals of Family Medicine* 6(2): 138-145.

Kjeldmand D. and Holmstrom I (2010). Difficulties in Balint groups: a qualitative study of leaders' experiences. *British Journal of General Practice* 60(580): 808-814.

Kjeldmand D, Holmstrom I, Rosenqvist U (2004) Balint training makes GPs thrive better in their job. *Patient education and counseling* 55(2): 230-235.

Kjeldmand D, Holmstrom I, Rosenqvist U (2006) How patient-centred am I? A new method to measure physicians' patient-centredness. *Patient Education & Counseling* 62(1): 31-37.

Lee E & Kealy D (2014). Revisiting Balint's innovation: enhancing capacity in collaborative mental health care. *Journal of Interprofessional Care* 28(5): 466-470.

Leggett A (2012). Transcultural issues in the dynamics of a Balint clinical reflection group for community mental health workers. *Transcultural Psychiatry* 49(2): 366-376.

Levenstein S (1978). A report of three years' experience of a Balint group in Cape Town. *South African Medical Journal*, 54(3): 121-123.

Levenstein S. (1981). An undergraduate Balint group in Cape Town. *South African Medical Journal*. 59(18): 642-643.

Levenstein S (1982). An undergraduate Balint group in Cape Town - A follow-up report. *South African Medical Journal* 62(3): 89-90.

Margalit AP, Glick SM, Benbassat J, Cohen A (2004) Effect of a biopsychosocial approach on patient satisfaction and patterns of care. *Journal of General Internal Medicine* 19(5 Pt 2): 485-491.

Nielsen HG & Tulinius C (2009) Preventing burnout among general practitioners: is there a possible route? *Education for Primary Care* 20(5): 353-359.

Salander Pä, Sandström M (2014) A Balint-inspired reflective forum in oncology for medical residents: Main themes during seven years. *Patient Education & Counseling* 97(1): 47-51.

Samuel O (1989) How doctors learn in a Balint group. *Family practice* 6(2): 108-113.

Sekeres MA, Chernoff M, Lynch TJ, Kasendorf EI, Lasser DH, Greenberg DB (2003) The impact of a physician awareness group and the first year of training on hematology-oncology fellows. *Journal of Clinical Oncology* 21(19): 3676-3682.

Smith M & Anandarajah G (2007) Mutiny on the balint: balancing resident developmental needs with the balint process. *Family medicine* 39(7): 495-497.

Stojanovic-Spehar S, Blazekovic-Milakovic S, Matanic D (2004) Education about pharmacotherapy and psychotherapy of anxiety among primary care physicians in Croatia: Balint group approach, *Croatian medical journal* 45(5): 625-629.

Torppa MA, Makkonen E, Martenson C, Pitkala KH (2008) A qualitative analysis of student Balint groups in medical education: contexts and triggers of case presentations and discussion themes. *Patient Education & Counseling* 72(1): 5-11.

Yakeley J, Shoenberg P, Morris R, Sturgeon D, Majid S (2011) Psychodynamic approaches to teaching medical students about the doctor-patient relationship: Randomised controlled trial. *Psychiatrist* 35(8): 308-313.

Caregiver Support Program

Heaney C (1991) Enhancing Social Support at the Workplace: Assessing the Effects of the Caregiver Support Program *Health Educ Behav* 18(4); 477-494.

Heaney C, Price RH, Rafferty J (1995) Increasing Coping Resources at Work: A Field Experiment to Increase Social Support, Improve Work Team Functioning, and Enhance Employee Mental Health. *Journal of Organizational Behavior*, 16 (4): 335-352.

Heaney CA, Price RH, & Rafferty J (1995) The caregiver support program: An intervention to increase employee coping resources and enhance mental health. In L. R. Murphy, J. J. J. Hurrel, S. L. Sauter, & G. P. Keita (Eds.), *Job stress interventions* (pp. 93-108). Washington, DC: American Psychological Association.

Clinical and Restorative Supervision

Primary studies:

Arvidsson B, Lofgren H, Fridlund B (2001) Psychiatric nurses' conceptions of how a group supervision programme in nursing care influences their professional competence: a 4-year follow-up study. *Journal of Nursing Management*, 9(3): 161-171.

Ardvidsson B, Skarsater I, Oijervall J, Fridlund B (2008) Process-oriented group supervision implemented during nursing education: nurses' conceptions 1 year after their nursing degree. *Journal of Nursing Management*, 16: 868–875.

Ashmore R, Carver N, Clibbens N, Sheldon J (2012) Lecturers' accounts of facilitating clinical supervision groups within a pre-registration mental health nursing curriculum. *Nurse Education Today* 32: 224-228.

Begat IBE, Severinsson, EI (2001) Nurses' reflections on episodes occurring during their provision of care-An interview study. *International Journal of Nursing Studies*, 38: 71-77.

Bogo M, Paterson J, Tufford L, King R (2011) Interprofessional clinical supervision in mental health and addiction: Toward identifying common elements. *The Clinical Supervisor*, 30:1, 124-140.

Bondas T (2010) Nursing leadership from the perspective of clinical group supervision: A paradoxical practice. *Journal of Nursing Management*, 18: 477-486.

Bradshaw T, Butterworth A, Mairs H (2007) Does structured clinical supervision during psychosocial intervention education enhance outcome for mental health nurses and the service users they work with? *Journal of Psychiatric and Mental Health Nursing*, 14: 4-12.

Brunero S, Lamont S (2012) The process, logistics and challenges of implementing clinical supervision in a generalist tertiary referral hospital. *Scand J Caring Sci*; 26; 186–193.

Buus N, Angel S, Traynor M, Gonge H (2010) Psychiatric hospital nursing staff's experiences of participating in group-based clinical supervision: An interview study. *Issues in Mental Health Nursing*, 31:10, 654-661.

Danielsson A, Sundin-Andersson C, Hov R, Athlin E (2009) Norwegian and Swedish preceptors' views of their role before and after taking part in a group supervision program. *Nurs Health Sci*, 11(2):107-113.

Davey B, Desousa C, Robinson S, Murrells T (2006) The policy--practice divide: Who has clinical supervision in nursing? *Journal of Research in Nursing*, 11(3): 237-248.

Davys AM & Beddoe L (2009) Interprofessional learning for supervision: 'taking the blinkers off'. *Learning in Health and Social Care*, 8(1): 58-69.

Edwards D, Burnard P, Hannigan B, Cooper L, Adams J, Juggessur T, Fothergil A, Coyle D (2006) Clinical supervision and burnout: The influence of clinical supervision for community mental health nurses. *Journal of Clinical Nursing* 15, 1007–1015.

Edwards D, Cooper L, Burnard P, Hannigan B, Adams J, Fothergill A, Coyle D (2005) Factors influencing the effectiveness of clinical supervision. *Journal of Psychiatric and Mental Health Nursing*, 12: 405-414.

Flackman B, Fagerberg I, Haggstrom E, Kihlgren A, Kihlgren M (2007) Despite shattered expectations a willingness to care for elders remains with education and clinical supervision. *Scand J Caring Sci*, 21: 379-389.

Gonge H & Buus N (2011) Model for investigating the benefits of clinical supervision in psychiatric nursing: A survey study. *International Journal of Mental Health Nursing*, 20: 102-111.

Gonge H & Buus N (2010) Individual and workplace factors that influence psychiatric nursing staff's participation in clinical supervision: a survey study and prospective longitudinal registration. *Issues in Mental Health Nursing*, 31:5,345-354.

Hallberg IR (1995) Clinical group supervision and supervised implementation of planned individualized care of severely demented people: effects on nurses, provision of the care, and patients. *Journal of Psychiatric and Mental Health Nursing*, 2: 111-114.

Hallberg IR (1994) Systematic clinical supervision in a child psychiatric ward: Satisfaction with nursing care, tedium, burnout, and the nurses' own report on the effects of it. *Archives of Psychiatric Nursing*, 8(1): 44-52.

Hall-Lord M, Theander K, Athlin E (2013) A clinical supervision model in bachelor nursing education -- Purpose, content and evaluation. *Nurse Education in Practice*, 13: 506-511.

Hansebo G & Kihlgren M. Nursing home care: changes after supervision. *Journal of Advanced Nursing* 45(3), 269–279.

Heaven C, Clegg J, Maguire P (2006) Transfer of communication skills training from workshop to workplace: The impact of clinical supervision. *Patient Education and Counseling* 60, 313–325.

Holmlund K, Lindgren B, Athlin E (2010) Group supervision for nursing students during their clinical placements: its content and meaning. *Journal of Nursing Management*, 18, 678–688.

Hyrkas K (2005) Clinical supervision, burnout, and job satisfaction among mental health and psychiatric nurses in Finland. *Issues in Mental Health Nursing*, 26:5, 531-556.

Hyrkas K, Appelqvist-Schmidlechner K, Haataja R (2006) Efficacy of clinical supervision: Influence on job satisfaction, burnout and quality of care. *Journal of Advanced Nursing*, 55(4): 521-535.

Jones A (2003) Some benefits experienced by hospice nurses from group clinical supervision. *European Journal of Cancer Care* 12, 224–232.

Jones 2001 Jones, A. Some experiences of professional practice and beneficial changes derived from clinical supervision by community Macmillan nurses. *European Journal of Cancer Care*, 10: 21-30.

Kelly B, Long A, McKenna H (2001) A survey of community mental health nurses' perceptions of clinical supervision in Northern Ireland. *Journal of Psychiatric and Mental Health Nursing*, 8: 33-44.

Kennedy TJT, Lingard, L, Baker, GR, Kitchen L, Regehr, G (2007) Clinical oversight: Conceptualizing the relationship between supervision and safety. *Society of General Internal Medicine*, 22:1080–1085.

Kenny A & Allenby A (2013) Implementing clinical supervision for Australian rural nurses. *Nurse Education in Practice*, 13: 165-169.

Kilcullen N (2007). An analysis of the experiences of clinical supervision on Registered Nurses undertaking MSc/graduate diploma in renal and urological nursing and on their clinical supervisors. *Journal of Clinical Nursing* 16, 1029–1038.

Knox S, Caperton W, Phelps D, Pruitt N (2014) A qualitative study of supervisees internal representations of supervisors. *Counselling Psychology Quarterly*, 27:4, 334-352.

Koivu A, Saarinen PI, Hyrkas K (2012) Who benefits from clinical supervision and how? The association between clinical supervision and the work-related well-being of female hospital nurses. *Journal of Clinical Nursing*, 21, 2567–2578.

Koivu A, Hyrka, SK, Saarinen PI (2011) Who attends clinical supervision? The uptake of clinical supervision by hospital nurses. *Journal of Nursing Management* 19, 69–79.

Koivu A, Saarinen PI, Hyrkas K (2012) Does clinical supervision promote medical–surgical nurses_ well-being at work? A quasi-experimental 4-year follow-up study *Journal of Nursing Management* 20, 401–413.

Lakeman R & Glasgow C (2009) Introducing peer-group clinical supervision: an action research project. *International Journal of Mental Health Nursing,* 18: 204-210.

Lantz I & Severinsson E (2001) The influence of focus group-oriented supervision on intensive care nurses' reflections on family members' needs. *Intensive and Critical Care Nursing*, 17: 128-137.

Lindgren B & Athlin E (2010) Nurse lecturers' perceptions of what baccalaureate nursing students could gain from clinical group supervision. *Nurse Education Today* 30:360-364.

Lindgren B, Brulin C, Holmlund K, Athlin E (2005) Nursing students' perception of group supervision during clinical training. *Journal of Clinical Nursing* 14, 822–829.

Magnusson A, Lutzen K, Severinsson E (2002) The influence of clinical supervision on ethical issues in home care of people with mental illness in Sweden. *Journal of Nursing Management*, 10: 37-45.

Malin NA (2000) Evaluating clinical supervision in community homes and teams serving adults with learning disabilities. *Journal of Advanced Nursing*, 31(3): 548-557.

O'Connell B, Ockerby CM, Johnson S, Smenda H, Bucknall TK (2011) Team clinical supervision in acute hospital wards: a feasibility study. *Western Journal of Nursing Research*, 35(3): 330-347.

Odling G, Danielson E, Jansson L (2001) Caregivers' descriptions of patients with advanced breast cancer in connection with supervision sessions in a surgical ward. *Cancer Nursing*, 24(1): 28-34.

Olofsson B (2005) Opening up: psychiatric nurses' experiences of participating in reflection groups focusing on the use of coercion. *Journal of Psychiatric and Mental Health Nursing*, 12: 259-67.

Olsson (1998) Systematic clinical supervision of home carers working in the care of demented people who are at home: structure content and effect as experienced by participants. *Journal of Nursing Management,* 6: 239-46.

Palsson M-BE, Hallberg IR, Norbert A (1994) Systematic clinical supervision and its effects for nurses handling demanding care situations: interviews with Swedish district nurses and hospital nurses in cancer care. *Cancer Nursing* 17(5): 385-394.

Pesut DJ & Williams CA (1990) The nature of clinical supervision in psychiatric nursing: a survey of clinical specialists. *Archives of Psychiatric Nursing* 4(3): 188-194.

Saarikoski M, Marrow C, Abreu W, Riklikiene O, O'zbicakc S (2007) Student nurses' experience of supervision and Mentorship in clinical practice: A cross cultural perspective. *Nurse Education in Practice*, 7, 407–415.

Severinsson EI, Hallberg IR (1996) Systematic clinical supervision, working milieu and influence over duties: the psychiatric nurse's viewpoint -a pilot study. *Int J. Nurs. Stud.*, 33(4): 394-406.

Sirola-Karvinen P, Hyrkas K (2008) Administrative clinical supervision as evaluated by the first-line managers in one health care organization district. *Journal of Nursing Management*, 16: 588-600.

Sloan G (1999) Good characteristics of a clinical supervisor: a community mental health nurse perspective. *Journal of Advanced Nursing*, 30(3), 713-722.

Walsh K, Nicholson J, Keough C, Pridham R, Kramer M, Jeffrey J (2003) Development of a group model of clinical supervision to meet the needs of a community mental health nursing team. *International Journal of Nursing Practice*, 9: 33-39.

White E, Butterworth T, Bishop V, Carson J, Jeacock J, Clements A (1998) Clinical supervision: insider reports of a private world. *Journal of Advanced Nursing* 28(1), 185-192.

White E & Winstanley J (2009) A randomised controlled trial of clinical supervision: selected findings from a novel Australian attempt to establish the evidence base for causal relationships with quality of care and patient outcomes, as an informed contribution to mental health nursing practice development. *Journal of Research in Nursing*, 15(2): 151-167.

Williams L & Irvine F (2009) How can the clinical supervisor role be facilitated in nursing: a phenomenological exploration. *Journal of Nursing Management* 17: 474-483.

Secondary studies (Literature reviews):

Brunero S & Stein-Parbury J (2008) The effectiveness of clinical supervision in nursing: an evidenced based literature review. *Australian Journal of Advanced Nursing*, 25(3):86-94.

Butterworth T, Bell L, Jackson C, Majda P (2008) Wicked spell or magic bullet? A review of the clinical supervision literature 2001–2007. *Nurse Education Today*, 28: 264-72.

Dawson M, Phillips B, Leggat S (2013) Clinical Supervision for Allied Health Professionals: A Systematic Review. *J Allied Health,* 42(2):65–73.

Ducat WH, Kumar S (2015) A systematic review of professional supervision experiences and effects for allied health practitioners working in non-metropolitan health care settings. *Journal of Multidisciplinary Healthcare*, 8:397-407.

Farnan JM, Petty LA, Georgitis E, Martin S, Chiu E, Prochaska M, Arora VM (2012) A Systematic Review: The Effect of Clinical Supervision on Patient and Residency Education Outcomes *Academic Medicine*, 87(4): 428-442.

Henshaw A-M, Clarke D, Long AF (2013) Midwives and supervisors of midwives' perceptions of the statutory supervision of midwifery within the United Kingdom: A systematic review. *Midwifery*, 29: 75–85.

Kilminster SM & Jolly BC (2000) Effective supervision in clinical practice settings: a literature review. *Medical Education*, 34:827-840.

Pearce P, Phillips B, Dawson M, Leggat SG (2013) Content of clinical supervision sessions for nurses and allied health professionals: a systematic review. *Clinical Goverance: An international journal*, 18(2): 139-154.

Wheeler S & Richards K (2007) *The impact of clinical supervision on counsellors and therapists, their practice and their clients: a systematic review of the literature* (BACP). British Association for Counselling and Psychotherapy: Lutterworth.

Resilience Training

Mealer M, Conrad D, Evans J, Jooste K, Solyntjes J, Rothbaum B, Moss M (2014) Feasibility and Acceptability of a Resilience Training Program for Intensive Care Unit Nurses, *Am J Crit Care*, 23(6): e97-105.

Peng L, Min L, Zuo X, Miao Y, Chen L, Yu Y, Liu B, Wang T (2014) Application of the Pennsylvania resilience training program on medical students. *Personality and Individual Differences*, 61-62: 47-51.

Sharma V, Bauer B, Prasad K, Sood A, Schroeder D (2012) Self help intervention to decrease stress and increase mindfulness: A pilot trial. *BMC Complement Altern Med*, 12(suppl 1): P253.

Sharma V, Sood A, Prasad K, Loehrer L, Schroeder D, Brent B (2014) Bibliotherapy to decrease stress and anxiety and increase resilience and mindfulness: A pilot trial. *Explore* (NY), 10(4): 248-52 *(reports on the same study as the 2012 paper).*

Sood A, Prasad K, Schroeder D, Prathibha V (2011) Stress management and resilience training among department of medicine faculty: A pilot randomized clinical trial. *J Gen Intern Med*, 26(8): 858-61.

Varker T & Devilly GJ (2012) An analogue trial of inoculation/resilience training for emergency services personnel: Proof of concept. *J Anxiety Disord*, 26(6): 696-701.

Critical Incidence Stress Debriefing

Matthews LR (1998) Effect of Staff Debriefing on Posttraumatic Stress Symptoms After Assaults by Community Housing Residents. *Psychiatric Services*, 49(2): 207-212.

O'Connor J & Jeavons S (2003) Perceived effectiveness of critical incident stress debriefing by Australian nurses. *Australian Journal of Advanced Nursing*, 20(4): 22-29.

Peer Supported Story Telling

Macpherson CF (2008) Peer-supported storytelling for grieving pediatric oncology nurses *J Pediatr Oncol Nurs* 25(3): 148-163.

Reflective Practice Groups

Abbas K & Pierce CM (2013) Time to reflect: Reflective practice groups for consultants and registrars on a neonatal and paediatric intensive care unit (N/PICU). *24th Annual Meeting of the European Society of Paediatric and Neonatal Intensive Care, ESPNIC 2013 Rotterdam Netherlands,* 39, S51-S52.

Binks C, Jones FW & Knight K (2013) Facilitating reflective practice groups in clinical psychology training: A phenomenological study. *Reflective Practice,* 14: 305-318.

Boyd EM & Fayles AW (1983) Reflective learning: Key to learning from experience. *Journal of Humanistic Psychology*, 23: 99–117.

Dawber C (2013) Reflective practice groups for nurses: A consultation liaison psychiatry nursing initiative: Part 2 - the evaluation. *International Journal of Mental Health Nursing,* 22: 241-248.

Gould B & Masters H (2004). Learning to make sense: the use of critical incident analysis in facilitated reflective groups of mental health student nurses. *Learning in Health & Social Care,* 3: 53-3.

Hansom J & Butler M (2003) Sharing reflections in midwifery practice. *British Journal of Midwifery,* 11: 34-37.

Hayes J (1998) Learning from practice: developing the reflective skills of forensic psychiatric nurses. *Psychiatric Care,* 5: 30-33.

Heneghan C, Wright J, & Watson G (2014) Clinical psychologists' experiences of reflective staff groups in inpatient psychiatric settings: A mixed methods study. *Clinical Psychology & Psychotherapy,* 21:324-340.

Hong LP & Chew L (2008) Reflective practice from the perspectives of the bachelor of nursing students in International Medical University (IMU). *Singapore Nursing Journal,* 35:5.

Johnston J & Paley G (2013) Mirror mirror on the ward: Who is the unfairest of them all? Reflections on reflective practice groups in acute psychiatric settings. *Psychoanalytic Psychotherapy,* 27:170-186.

Kiff J, Holmes G, & Cushway D (2010) Personal awareness / development groups and clinical training. *Clinical Psychology Forum*, 207: 30–34.

Knight K, Sperlinger D, & Maltby M (2010) Exploring the personal and professional impact of reflective practice groups: A survey of 18 cohorts from a UK clinical psychology training course. *Clinical Psychology & Psychotherapy,* 17: 427-437.

Kung JW, Eisenberg RL, & Slanetz PJ (2012) Reflective Practice as a Tool to Teach Digital Professionalism. *Academic Radiology,* 19: 1408-1414.

Manning A, Cronin P, Monaghan A & Rawlings-Anderson K (2009) Supporting students in practice: an exploration of reflective groups as a means of support. *Nurse Education in Practice,* 9: 176-183.

Parish C, Bradley L, & Franks V (1997) Managing the stress of caring in ITU: a reflective practice group. *British journal of nursing (Mark Allen Publishing),* 6: 1192-1196.

Platzer H, Blake D, & Ashford D (2000) An evaluation of process and outcomes from learning through reflective practice groups on a post-registration nursing course. *Journal of advanced nursing,* 31: 689-695.

Schon D (1983) *The Reflective Practitioner: How Professionals Think in Action*. New York, NY: Basic Books.

Psychosocial Intervention Training

Redhead K; Bradshaw T; Braynion P; Doyle M (2011) An evaluation of the outcomes of psychosocial intervention training for qualified and unqualified nursing staff working in a low-secure mental health unit. *Journal of Psychiatric & Mental Health Nursing*, 18(1): 59-66.

Ewers P; Bradshaw T; McGovern J; Ewers B (2002) Does training in psychosocial interventions reduce burnout rates in forensic nurses? *Journal of Advanced Nursing* 37(5): 470-476.

Doyle M, Kelly D, Clarke S, Braynion P(2007) Burnout: the impact of psychosocial interventions training. *Mental Health Practice* 10: 16–19.

Mindfulness Based Stress Reduction

Bazarko D, Cate RA, Azocar F, Kreitzer MJ (2013) The impact of an innovative mindfulness-based stress reduction program on the health and well-being of nurses employed in a corporate setting. *Journal of Workplace Behavioral Health*, 28(2): 107-133.

Beddoe AE. Murphy SO (2004) Does mindfulness decrease stress and foster empathy among nursing students? *J.Nurs.Educ*.43(7): 305-312.

Brady S, O'Connor N, Burgermeister D, Hanson P (2012) The Impact of Mindfulness Meditation in Promoting a Culture of Safety on an Acute Psychiatric Unit. *Perspect.Psychiatr.Care*, 48(3): 129-137.

Cohen-Katz J, Wiley S, Capuano T, Baker DM, Deitrick L, Shapiro S (2005) The effects of mindfulness-based stress reduction on nurse stress and burnout: a qualitative and quantitative study, part III. *Holist.Nurs.Pract*, 19(2): 78-86.

de Vibe M, Solhaug I, Tyssen R, Friborg O, Rosenvinge JH, Sorlie T, Bjorndal A (2013) Mindfulness training for stress management: a randomised controlled study of medical and psychology students. *BMC medical education*, 13: 107.

Foureur M, Besley K, Burton G, Yu N, Crisp J (2013) Enhancing the resilience of nurses and midwives: Pilot of a mindfulness-based program for increased health, sense of coherence and decreased depression, anxiety and stress. *Contemporary Nurse*, 45(1): 114-125.

Geary C, Rosenthal S, (2011) Sustained impact of MBSR on stress, well-being, and daily spiritual experiences for 1 year in academic health care employees. *The Journal of Alternative and Complementary Medicine*, 17(1): 939-944.

Goodman MJ, Schorling JB (2012) A mindfulness course decreases burnout and improves well-being among healthcare providers. *Int.J.Psychiatry Med*., 43(2): 119-128.

Hallman I, O'Connor, N, Hasenau, S, Brady S (2014) Improving the Culture of Safety on a High-Acuity Inpatient Child/Adolescent Psychiatric Unit by Mindfulness-Based Stress Reduction Training of Staff. *J.Child Adolesc.Psychiatr.Nurs*. 27(4): 183-189.

Irving JA, ParkSaltzman J, Fitzpatrick M, Dobkin PL, Chen A, Hutchinson T (2014) Experiences of health care professionals enrolled in mindfulness-based medical practice: A grounded theory model. *Mindfulness*, 5(1): 60-71.

Mackenzie CS, Poulin PA, Seidman-Carlson R (2006) A brief mindfulness-based stress reduction intervention for nurses and nurse aides. *Applied Nursing Research*, 19(2): 105-109.

Martin-Asuero A, Garcia-Banda G (2010) The mindfulness-based stress reduction program (MBSR) reduces stress-related psychological distress in healthcare professionals. *Spanish Journal of Psychology*, 13(2): 897-905.

Marx R, Strauss C, Williamson C, Karunavira T (2014) The eye of the storm: A feasibility study of an adapted mindfulness-based cognitive therapy (MBCT) group intervention to manage NHS staff stress. *The Cognitive Behaviour Therapist*, 7 Dec, Art e18-17.

Moody K; Kramer D, Santizo RO, Magro L, Wyshogrod D, Ambrosio J, Castillo C, Lieberman R, Stein J (2013) Helping the helpers: Mindfulness training for burnout in pediatric oncology-a pilot program. *Journal of Pediatric Oncology Nursing,* 30(5): 275-284.

Poulin PA, Mackenzie CS, Soloway G, Karayolas E (2008) Mindfulness training as an evidenced-based approach to reducing stress and promoting well-being among human services professionals. *International Journal of Health Promotion and Education*, 46(2): 72-80.

Rosenzweig S, Reibel DK, Greeson JM, Brainard GC, Hojat M (2003) Mindfulness-based stress reduction lowers psychological distress in medical students. *Teach.Learn.Med.*, 15(2): 88-92.

Shapiro SL, Astin JA, Bishop SR, Cordova M (2005) Mindfulness-Based Stress Reduction for Health Care Professionals: Results From a Randomized Trial. *International Journal of Stress Management*, 12(2): 164-176.

Shapiro SL, Schwartz GE, Bonner G (1998) Effects of mindfulness-based stress reduction on medical and premedical students. *J.Behav.Med.*,21(6): 581-599.
