# Supplementary material for: Can Schwartz Center Rounds support healthcare staff with emotional challenges at work, and how do they compare with other interventions aimed at providing similar support? A systematic review and scoping reviews
Source: BMJ Open. 2018 Oct 18;8(10):e024254. doi: 10.1136/bmjopen-2018-024254 (PMC6196967; doi:10.1136/bmjopen-2018-024254)
Supplement: Supplementary file 3 [file bmjopen-2018-024254supp003.pdf]

Supplementary File 3: Evidence base (in healthcare professionals) for Schwartz Rounds® and comparative interventions

Intervention	Healthcare settings	Number/type of papers included	Intervention fidelity between studies	Key Findings to:<br>Self/ Others/ Organisation	Overall strength of the evidence base*
**Schwartz Center Rounds®**	Hospitals, hospices, and educational settings (e.g. medical schools) – open to all staff of all grades (clinical and non-clinical).	10 papers (8 studies):<br>- 2 quantitative<br>- 1 qualitative<br>- 5 mixed methods	Yes as Rounds are licenced and have to be run according to a format subsequent to obtaining a licence.	**Self**<br>*Wellbeing:* reduced stress, emotional labour, and isolation at work<br>*Coping:* improved ability to cope with emotional difficulties at work; confidence in handling sensitive issues<br>*Self-reflection*: increased self-reflection/validation of experiences<br>*Knowledge*: students report increased knowledge/understanding about emotional side of providing patient care.<br><br>**Others**<br>*Patients*: increased compassion and empathy, increased interaction.<br>*Colleagues*: improved teamwork, communication, compassion/empathy<br><br>**Organisation**<br>*Access*: improved access to specific services (e.g palliative care)<br>*Culture change*: having dialogue that doesn't happen elsewhere; reinforced shared values and support strategic vision; reduced hierarchy	Quantitative: low/moderate due to cross-sectional design, non-validated measures, and lack of control (non-attender) data.<br>Qualitative: moderate due to limited reporting of theoretical underpinnings and strategies to improve rigour (e.g. deviant case analysis; reflexivity)<br>Mixed methods: Low

Intervention	Healthcare settings	Number/type of papers included	Intervention fidelity between studies	Key Findings to: Self/ Others/ Organisation	Overall strength of the evidence base*
**Action Learning Sets**	Pre/post registration education/training; primary care (e.g. online networks for GPs); acute and mental health services; palliative and continuing care; care homes and prisons.	14 papers:<br>- 6 quantitative<br>- 4 qualitative<br>4 mixed methods	Most hybrid interventions where ALS only one component. Group sizes between 4-8 members. Evaluation period 3months to a year, and 2- 6 sessions.	**Self**<br>*Empowerment:* foster greater psychological empowerment; sense of self-efficacy, self-esteem and confidence<br>*Awareness:* offer opportunity to explore self<br><br>**Others**<br>*Colleagues:* improved understanding and knowledge of colleagues; offers opportunity to share experiences and give/receive peer support<br><br>**Organisation**<br>*Workforce*: opportunities for mentoring and advice; satisfaction with the intervention being accessible and inclusive; possible limitations include having conflicting commitments and lack of group cohesiveness	Quantitative: Low due to lack of validated measures, and small sample sizes that were underpowered.<br><br>Qualitative: Moderate to high quality: some well-designed studies but others lacked detail on analytical strategy, limited transparency of findings (e.g. deviant cases not discussed, quotes not fitting themes and/or author interpretation)<br><br>Mixed methods: Low
**After Action Reviews (AAR)**	Acute settings	2 papers:<br>2 quantitative	Variability evident: 2 year evaluation of post-fall huddles vs. cross-sectional evaluation of one-off	**Self**<br>*Empowerment:* improved confidence in dealing with difficult situations<br><br>**Others**<br>*Patients:* improved communication and listening skills	Quantitative: Low to moderate – mostly non-experimental designs with convenience samples and non-validated (study-specific)

**Intervention**	**Healthcare settings**	**Number/type of papers included**	**Intervention fidelity between studies**	**Key Findings to: Self/ Others/ Organisation**	**Overall strength of the evidence base***
			training in AAR	**Organisation**<br>*Practice:* reduced task and coordination errors; increased uptake of post-fall huddles; positive impact on patient care	outcome measures; poor survey response.
**Balint groups**	Primary care (predominantly GPs), medical schools, acute settings	26 papers:<br>- 12 quantitative<br>- 6 qualitative<br>- 8 mixed methods	Principles applied consistently but some variations or hybrid-models (e.g. Balint-inspired roundtable groups)	**Self**<br>*Awareness:* raised personal awareness in doctors; helped medical students build a professional identity as doctors<br>*Resilience:* reduced stress; helped doctors and students resolve challenging situations in practice<br>*Job satisfaction*: improved job satisfaction in doctors<br><br>**Others**<br>*Patients:* foster positive attitudes towards ‘difficult’ patients; improve students’ ability to relate with patients; improve patient-centredness<br>*Colleagues:* promote teamwork<br><br>**Organisation**<br>*Practice:* reduced unnecessary prescriptions; increased uptake of psychosocial support; higher patient satisfaction	Quantitative: Low – mostly observational studies that lack randomisation, use non-validated measurement tools, and inadequately control confounding variables.<br><br>Qualitative: Moderate – mostly reflective case studies, with loose adherence to qualitative designs and limited description of approaches to rigour.<br><br>Mixed methods: Low

Intervention	Healthcare settings	Number/type of papers included	Intervention fidelity between studies	Key Findings to:<br>Self/ Others/ Organisation	Overall strength of the evidence base*
**Caregiver Support Program**	Mental health /learning disability homes	3 papers:<br>- 3 quantitative (all the same study)	No variability – has only been used and evaluated in one study.	**Self**<br>*Empowerment:* measured ability to cope with common work problems and ability to influence decision making (non-significant improvement);<br>*Wellbeing:* psychological wellbeing (non-significant improvement)<br><br>**Others**<br>*Colleagues:* greater supervisor support, less undermining, greater praise and feedback<br><br>**Organisation**<br>*Workforce:* No change in outcomes for managers (only direct-care staff); "train the trainers" element (to translate intervention to workplace) did not work	Quantitative: Moderate to low – the main limitations concern the sample (low response rates to surveys), method of analysis (did not use all the data, could have included people responding at only one time point) and measures (use of measures that were not well validated)
**Clinical and Restorative Supervision**	Nursing education leadership, and nursing generally (including mental health, paediatrics, addiction, elderly and dementia care, oncology, hospice nursing), and in	64 papers:<br>9 Secondary studies (literature reviews)<br>55 Primary studies: - 22 quantitative<br>27 qualitative<br>- 6 mixed methods	Wide variability in every aspect. Duration ranged from 1 mth to 5 yrs (median 12 mths). Session length ranged from 1- 3hrs	**Self**<br>*Awareness*: improved knowledge and insights, professional awareness and development<br>*Empowerment:* improved communication skills, job resources, professional efficacy and capacity for reflection<br>*Wellbeing*: reduced psychological distress; improved vitality; reduced stress; reduced burnout (lower emotional	Quantitative: Mostly Low/Moderate. Most are cross-sectional studies, with a few pre-post (mostly without control groups) and short-term follow-up. Only a few RCTs have been conducted and these

Intervention	Healthcare settings	Number/type of papers included	Intervention fidelity between studies	Key Findings to:<br>Self/ Others/ Organisation	Overall strength of the evidence base*
	emergency departments.		(median 1.5hrs), and frequency from weekly to monthly. Most were group supervision, ranging from 2 to 12 supervisees. Treatment fidelity was rarely referred to.	exhaustion and depersonalisation); improved sense of security, belonging and encouragement<br>*Resilience:* improved rational coping<br>*Job stress/satisfaction*: reduced job stress; improved job satisfaction<br><br>**Others**<br>Patients: better knowledge of patients' suffering and how to take responsibility; improved individualised documentation in patient notes;<br>Colleagues: foster solidarity, sharing and reflecting<br><br>***Organisation***<br>*Practice:* Reduced positive and total symptoms (in patients with psychosis) reported by student nurses<br>*Workforce:* staff retention<br><br>Continuity and quality of supervision are key moderators of outcomes.	are moderate-high quality. Inadequate sample sizes and unvalidated/ unreliable measures in most (mostly self-report of supervisor/supervisee rather than impact on patient/care).<br><br>Qualitative: Low/Moderate. Majority lack conceptual/theoretical bases; have inappropriate sampling and lack of transparency in methods/analysis.<br><br>Mixed methods: Low
**Critical Incidence Stress Debriefing**	Acute and community settings.	2 papers:<br>-2 quantitative	Fairly consistent approach to CISD following the	**Self**<br>*Wellbeing:* no significant impact on stress reduction, but significantly lower PTSD scores; feeling part of a group and realising they were not alone	Quantitative: Moderate –weaknesses or limited reporting of group allocation, sampling approach,

Intervention	Healthcare settings	Number/type of papers included	Intervention fidelity between studies	Key Findings to: Self/ Others/ Organisation	Overall strength of the evidence base*
			Mitchell model (1983). Details of the content of the intervention lacking. Consistency in approach between group facilitators questionable.	**Others**<br>*Colleagues:* opportunity for sharing experiences, and learning from others<br><br>**Organisation**<br>*Workforce:* potentially negative impacts included increased intrusive thoughts	consideration of confounding variables, and psychometric values of instruments used.
**Mindfulness-based Stress Reduction**	Acute and community settings, with doctors and nurses (qualified and in-training) and a range of allied health professions.	17 papers:<br>-15 quantitative<br>-1 qualitative<br>-1 mixed method	Mostly informed by Kabat-Zinn (1990).<br>One study combined face-to-face and telephone delivery of sessions; and five studies reported on a shortened version.<br>Content of sessions was similar across	**Self**<br>*Wellbeing:* improved relaxation and mood. Some mixed results in relation to burnout, stress, anxiety, distress, and satisfaction with life<br>*Resilience:* improved self-compassion, self-care, acceptance and coping skills<br>**Others**<br>*Colleagues:* opportunity for peer support	Quantitative: Moderate due to non-probability sampling, bias from confounding and lack of power calculations.<br>Qualitative: Moderate due to limited reporting of approaches to enhancing rigour (e.g. member checking, deviant cases, reflexivity, data saturation).<br>Mixed Methods: Low

Intervention	Healthcare settings	Number/type of papers included	Intervention fidelity between studies	Key Findings to:<br>Self/ Others/ Organisation	Overall strength of the evidence base*
			studies, but many lacked detail to judge fidelity.		
**Peer-supported storytelling**	Paediatric 17 papers<br>-15 quantitative<br>-1 qualitative<br>-1 mixed method<br>nurses who had experienced grief	1 paper:<br>- mixed methods	Only one study consisted of 3 self-selected dyads (6 nurses). Each dyad met biweekly for 2 months for brief informal storytelling sessions (each member taking a turn telling a story and listening at each session, mean length 17mins).	**Self**<br>*Wellbeing:* positive impact on grief<br>*Resilience:* positive impact on meaning-making, making sense of, and identifying benefit in, their experiences<br><br>**Others**<br>*Colleagues:* opportunity to receive and provide support during sessions<br><br>**Organisation**<br>*Workforce:* significant positive correlation between number of 'special' patient deaths during career and impact of sessions on grief	Quantitative: Low due to small (n=6), self-selected sample and no comparison group<br><br>Qualitative: Moderate due to descriptive approach to analysis, lacking in theoretical saturation<br><br>Mixed methods: Low
**Psychosocial intervention training**	Mental health settings in the UK	3 papers:<br>-3 quantitative	Consistent programme; delivered weekly over 4-5 months.	**Self**<br>*Wellbeing:* improved burnout (emotional exhaustion, depersonalisation and personal achievement)	Quantitative: Moderate –all 3 studies were quasi-experimental, utilising non-probability sampling,

Intervention	Healthcare settings	Number/type of papers included	Intervention fidelity between studies	Key Findings to:<br>Self/ Others/ Organisation	Overall strength of the evidence base*
			Variable duration between 4 to 8 months.	<u>*Empowerment:*</u> improved knowledge of, and attitude towards, mental illness and psychosocial approaches	but lacking a power calculation and not clearly accounting for confounding variables.
**Reflective Practice Groups**	Clinical psychology, psychotherapy, nursing, medicine, midwifery and radiology.	8 papers:<br>-3 quantitative<br>-3 qualitative<br>-2 mixed methods	All described as facilitated groups that explore practice related issues. Can last between 45-90 minutes; held weekly, fortnightly or monthly; group sizes varied from six to 20 attendees, with one or two facilitators.	**Self**<br><u>*Awareness:*</u> improved self-awareness and clinical insight<br><u>*Empowerment:*</u> increased confidence and capacity for reflection; better understanding of psychological ideas<br><u>*Wellbeing:*</u> increased ability to cope with stress<br><br>**Others**<br><u>*Colleagues:*</u> opportunity for peer support, sharing experience and learning; improved communication skills<br><u>*Patients:*</u> positive impact on empathy<br><br>**Organisation**<br><u>*Practice:*</u> perceived improvements in the quality of care provided	Quantitative: Low – mostly weakened by the absence of probability sampling, lack of validated tools, missing baseline measurements and inadequate control for confounding variables.<br><br>Qualitative: Low – mostly weakened by absence of an overall design and inadequate attention to data saturation, sampling and researcher reflexivity.<br><br>Mixed methods: Low
**Resilience Training**	Medical students, intensive care unit nurses, general medicine,	6 papers:<br>- 6 quantitative (reporting on five studies)	Two studies (three papers) evaluated resilience	**Self**<br><u>*Wellbeing:*</u> reduced depression, PTSD, stress and anxiety	Quantitative: Moderate –mostly pilot studies to determine the feasibility,

Intervention	Healthcare settings	Number/type of papers included	Intervention fidelity between studies	Key Findings to:<br>Self/ Others/ Organisation	Overall strength of the evidence base*
	emergency service personnel.		training alone. The remainder combined resilience training with other interventions. Interventions varied from 10-12 weeks of regular training, to 40min one-off.	*Resilience:* improved cognitive appraisal, control of stress; decreased negative emotion and expression inhibition	acceptability or prove the concept of the intervention. Limitations included only comparing within groups (despite having control group), ambiguous details regarding sampling and/or lacking control group.

References:

Kabat-Zinn, J. (1990). *Full catastrophe living: Using the wisdom of your body and mind to face stress, pain and illness.* New York: Delacorte

Mitchell, J. T. (1983). When disaster strikes: The critical incident stress debriefing process. Journal of Emergency Medical Services, **8**(1):36-9.
