# Supplementary material for: Can Schwartz Center Rounds support healthcare staff with emotional challenges at work, and how do they compare with other interventions aimed at providing similar support? A systematic review and scoping reviews
Source: BMJ Open. 2018 Oct 18;8(10):e024254. doi: 10.1136/bmjopen-2018-024254 (PMC6196967; doi:10.1136/bmjopen-2018-024254)
Supplement: Author's manuscript [file bmjopen-2018-024254.draft_revisions.pdf]

BMJ Open is committed to open peer review. As part of this commitment we make the peer review history of every article we publish publicly available.

When an article is published we post the peer reviewers' comments and the authors' responses online. We also post the versions of the paper that were used during peer review. These are the versions that the peer review comments apply to.

The versions of the paper that follow are the versions that were submitted during the peer review process. They are not the versions of record or the final published versions. They should not be cited or distributed as the published version of this manuscript.

BMJ Open is an open access journal and the full, final, typeset and author-corrected version of record of the manuscript is available on our site with no access controls, subscription charges or pay-per-view fees (http://bmjopen.bmj.com).

If you have any questions on BMJ Open's open peer review process please email info.bmjopen@bmj.com

BMJ Open

Can Schwartz Centre Rounds support healthcare staff with emotional challenges at work, and how do they compare to other interventions aimed at providing similar support? A systematic review and scoping reviews

Journal:	*BMJ Open*
Manuscript ID	bmjopen-2018-024254
Article Type:	Research
Date Submitted by the Author:	17-May-2018

Complete List of Authors:	Taylor, Cath; University of Surrey, School of Health Sciences<br>Xyrichis, Andreas; King's College London, Florence Nightingale Faculty of Nursing, Midwifery and Palliative Care<br>Leamy, Mary; King's College London, Florence Nightingale Faculty of Nursing, Midwifery and Palliative Care<br>Reynolds, Ellie; King's College London, Florence Nightingale Faculty of Nursing, Midwifery and Palliative Care<br>Maben, Jill; University of Surrey, School of Health Sciences

Keywords:	Schwartz Rounds, Occupational Stress, Staff Wellbeing, Systematic Review, Compassion, Reflection

For peer review only

SCHOLARONE™ Manuscripts

Can Schwartz Centre Rounds support healthcare staff with emotional challenges at work, and how do they compare to other interventions aimed at providing similar support? A systematic review and scoping reviews

Authors:

*Cath Taylor, School of Health Sciences, University of Surrey, Guildford GU2 7XH, UK

Email: cath.taylor@surrey.ac.uk Tel: +44 1483 688862

Andreas Xyrichis, Florence Nightingale Faculty of Nursing, Midwifery and Palliative Care, Kings College London, London UK

Mary Leamy, Florence Nightingale Faculty of Nursing, Midwifery and Palliative Care, Kings College London, London UK

Ellie Reynolds, Florence Nightingale Faculty of Nursing, Midwifery and Palliative Care, Kings College London, London UK

Jill Maben, School of Health Sciences, University of Surrey, Guildford, UK

*corresponding author

Word count (excluding title page, abstract, references, figures and tables): 4024

For peer review only

Abstract

Objectives: (i) To synthesise the evidence-base for Schwartz Center Rounds® (Rounds) to assess any impact on healthcare staff and identify key features; (ii) to scope evidence for interventions with similar aims, and compare effectiveness and key features to Rounds.

Design: Systematic review of Rounds literature; scoping reviews of comparator interventions (Action Learning Sets; After Action Reviews; Balint Groups; Caregiver Support Programme; Clinical Supervision; Critical Incident Stress Debriefing; Mindfulness-Based Stress Reduction; Peer Supported Storytelling; Psychosocial Intervention Training; Reflective Practice Groups; Resilience Training).

Data Sources: PsychINFO, CINAHL, MEDLINE, and EMBASE, internet search engines; consultation with experts.

Eligibility criteria: Empirical evaluations (qualitative or quantitative); any healthcare staff in any healthcare setting; published in English.

Results: The overall evidence base for Rounds is limited. We developed a composite definition to aid comparison with other interventions from 41 documents containing a definition of Rounds. Twelve (ten studies) were empirical evaluations. All were of low/moderate quality (weak study designs including lack of control groups). Findings showed the value of Rounds to attenders, with a self-reported positive impact on individuals, their relationships with colleagues and patients, and wider cultural changes. The evidence for the comparative interventions was scant and also low/moderate quality. Some features of Rounds were shared by other interventions, but Rounds offer unique features including being open to all staff and having no expectation for verbal contribution by attenders.

For peer review only - http://bmjopen.bmj.com/site/about/guidelines.xhtml

Conclusions: Evidence of effectiveness for all interventions considered here remains limited. Methods that enable identification of core features related to effectiveness are needed to optimise benefit for individual staff members and organisations as a whole. A systems approach conceptualising workplace wellbeing ~~as~~ arising from both individual and environmental/structural factors, and comprising interventions both for assessing and improving the wellbeing of healthcare staff, is required. Schwartz Rounds could be considered as one strategy to enhance staff wellbeing.

Keywords: Schwartz Rounds, Occupational Stress, Staff wellbeing, Systematic Review, Compassion, Reflection

For peer review only

Article Summary: Strengths and limitations of this study

- This is the first systematic review of Schwartz Center Rounds®, a healthcare staff intervention from the US that has spread rapidly through UK healthcare organisations
- Additional scoping reviews of 11 interventions with similar aims to support the wellbeing of healthcare staff, enables a novel comparative analysis to key features of Schwartz Rounds.
- This paper compares other staff wellbeing interventions to Rounds, thereby resulting in a focus on key features of Rounds; we did not explicitly draw out key features of other interventions or compare them against each other.
- The use of scoping reviews for comparator interventions, and exclusion of evidence in populations other than healthcare staff means that some evidence may have been omitted.
- The heterogeneity of study designs and outcomes, and weak study designs, means that findings are summarized narratively rather than using meta-analysis.

For peer review only - http://bmjopen.bmj.com/site/about/guidelines.xhtml

Introduction

In this paper, we report the systematic review of evidence regarding Schwartz Center Rounds® (Rounds) and conduct a comparative analysis to eleven interventions also broadly aimed at supporting healthcare staff with the emotional challenges of their work. In doing so, we define Rounds from the literature and discuss the future potential use of interventions to support staff with the emotional challenges of providing healthcare. Healthcare providers are amongst the largest employers in many countries worldwide. For example, the UK NHS employs 1.5 million staff,[1] and in 2014 there were approximately 1.8 million physicians,[2] and 3.4 million nurses[3] across the EU. Provision of healthcare relies upon both clinical and non-clinical staff (e.g. managers, administrators, porters/orderlies, caterers, and domestic staff) all of whom may be impacted by the emotional challenges they face in their interactions with the patients and families they come across in day to day life.

Numerous publications have highlighted the high prevalence of psychological morbidity amongst healthcare staff in both clinical and non-clinical roles, and in many different countries worldwide.[4-10] Indeed, studies have typically reported between a quarter to a third of healthcare staff to have levels of psychological distress indicative of the need for clinical intervention, and in the UK mental health reasons explain a third of all NHS sickness absence, costing approximately £1bn (of the total £2.4bn cost of sickness absence in 2015).[11] Together with the clear consequences of this for their wellbeing and quality of life, and impact on their families, there is now increasing recognition of the link between the wellbeing of healthcare staff and quality of patient care (in relation to both patient experience, and clinical outcomes).[12-15]

For peer review only - http://bmjopen.bmj.com/site/about/guidelines.xhtml

Consequently, the wellbeing of healthcare staff is high on the agenda of healthcare organisations in the UK and worldwide.[16-21] In the UK, NICE guidance published in 2009 recommended that organisations take a strategic approach to tackling staff wellbeing, encompassing approaches that focus on both prevention and treatment, and that include interventions for individuals as well as "organisation-wide approaches that encompass all employees".[22] However, the reviews underpinning this guidance highlighted the poor quality of evidence overall and in particular the limited evidence on organisation-wide policies or approaches, with the strongest evidence in relation to interventions aimed at stress management for individuals.[22]

Schwartz Rounds are a rare example of an organisation-wide intervention that has seen rapid spread across healthcare organisations in the UK.[23] Rounds originated in the U.S. where they now run in over 430 organisations. After a pilot introduction to two UK hospitals in 2009, they now run in over 170 UK health and social care organisations (hospitals, hospices, community settings). They were developed to support healthcare staff to deliver compassionate care by providing a safe space where staff could openly share and reflect on the emotional, social and ethical challenges faced at work. The premise is that caregivers will be more able to make personal connections with colleagues and patients if they have insight into their own responses and feelings. Their rapid adoption in the UK was despite a limited evidence base, though attendance at Rounds was reported to be associated with improved compassion for patients, better teamwork, and reduced stress in staff members, as well as having a positive impact on organisational culture.[24-25] Consequently, the National Institute for Health Research commissioned a national evaluation of Rounds that has recently concluded,[26] supporting these earlier findings and showing attendance at Rounds to be associated with a reduction in psychiatric morbidity. A key component of the evaluation,

For peer review only - http://bmjopen.bmj.com/site/about/guidelines.xhtml

intended to support organisational decision-making regarding staff wellbeing interventions, was to review the evidence for Schwartz Rounds and contextualise them by comparing the features of Rounds to other staff wellbeing interventions with similar aims. This paper reports the results from this, and thereby aims to answer the following review questions:

1) What are the defining features of Schwartz Centre Rounds®, and what is their evidence base?
2) What comparable interventions providing staff support/reflective space exist, what key features do they share with Schwartz Rounds and what is their evidence base in healthcare professionals?

Specifically, we aim to (i) identify key features of Rounds by synthesising published descriptions of Rounds to create a composite definition; (ii) systematically review and appraise all empirical evaluations of Rounds; (iii) identify comparative interventions, describe their key features and scope their evidence base; and (iv) document similarities and differences between Rounds and comparative interventions.

Methods

The review of Schwartz Rounds literature followed PRISMA systematic literature review guidance where applicable.

Search strategy

The search strategies for the systematic review of Rounds literature involved: (i) a traditional database search (PsychINFO, CINAHL, MEDLINE, and EMBASE to give comprehensive coverage of medical, psychological, nursing and social sciences literature). As an example, the MEDLINE database search for Schwartz Rounds was: (Schwartz adj2 Round*).mp. [mp=title, abstract, original title, name of substance word, subject heading word, keyword heading word, protocol supplementary concept word, rare disease supplementary concept word, unique identifier] (ii) use of internet search engines; and (iii) consultation with

For peer review only - http://bmjopen.bmj.com/site/about/guidelines.xhtml

experts. Inclusion criteria included having a health professional sample (either qualified or trainee) and empirically evaluate the intervention using qualitative and/or quantitative methods. The review excluded non-English language sources, unpublished dissertations/theses and any papers not accessible via the institution's online library, Google Scholar or directly from the journal website. All records were pooled together into a bibliographic database. First, records were screened to exclude duplicate entries. Second, the title and abstract of remaining records was reviewed for eligibility. All database searches were conducted between 14th October 2014 and 5th February 2015, though searches for Schwartz Rounds evaluations and consultation with experts continued until September 2017.

Data extraction and quality appraisal

Standard data items were extracted to describe included papers (e.g. citation, country, setting, population/sample, overall design etc) and the evaluation (e.g. length of evaluation; data collection method/s; outcome measures; key findings) using extraction sheets that were developed and piloted by all data extractors. In addition, items were developed that were specific to each intervention, for example, whether group or individual focused, size of group, length/number of sessions, content of sessions, whether facilitated or not (and if facilitated whether training/supervision was provided). Quality assessment of qualitative and quantitative primary studies was undertaken for each study using the tools developed by Jones et al[27] which include assessment of key criteria and then an overall rating (High - No or few flaws; Moderate - Some flaws; Low - Significant flaws). Mixed methods studies were, in addition, assessed against the six criteria for good reporting of mixed methods studies developed by O'Cathain et al[28]. Quality was rated low (<3 criteria were met); moderate (3-4) or high (5+).

For peer review only - http://bmjopen.bmj.com/site/about/guidelines.xhtml

Synthesis

Thematic analysis of the types of outcomes reported resulted in the identification of three categories relating to: a) self; b) others (e.g. patients, colleagues); or c) wider organisation (e.g. changes to policies; organisational metrics such as safety or satisfaction). Findings are presented according to these three categories. Finally the overall quality of the evidence base for each intervention is described based on the range in quality for individual studies.

Constructing a composite definition of Schwartz Rounds

In order to determine the key features of Rounds for comparison with other interventions, we constructed a 'composite' definition based on descriptions used in Rounds literature. For this process, we included all literature (including non-empirical literature for example letters, editorials) providing it included a description of Rounds. Text describing Rounds (what they were and their aims, e.g. structure and purpose, as well as any text describing what they were 'not') was extracted from published accounts. The text was analysed thematically by four team members independently (CT, JM, ML, MH), core concepts were discussed and agreed, and a single definition was produced. The face validity of the definition was confirmed after review by study advisory and steering group members.

Scoping reviews of comparative interventions

The reviews of comparable interventions followed an interpretative scoping literature review methodology based on the framework outlined by Arksey and O'Malley[29]. The searching, data extraction and synthesis followed similar steps to the review of Schwartz Rounds literature (except where noted below) but instead of producing a detailed critique and review of individual studies they were instead aimed at producing a summary description of the evidence base in relation to size, scope and quality, and used to extract data relevant for the

For peer review only - http://bmjopen.bmj.com/site/about/guidelines.xhtml

comparative analysis. For each intervention, the number/type of included papers was recorded, and each intervention was described in relation to its original format (e.g. number of participants, original setting and healthcare setting/s, and intended aims/outcomes); and the variability in its application within the literature (fidelity to original format). Main findings were examined across all interventions and analysed thematically (using the same categories as for Schwartz Rounds: self, others, organisation) to enable synthesis within, and comparison across, each intervention.

Identification of comparative interventions to include

We aimed to identify interventions that support health professionals with the emotional challenges of delivering patient care. Initially we identified aspects that were fundamental to Rounds, including providing an opportunity for reflection, disclosure, and offering psychological safety; and these informed choices regarding potential comparative interventions. Included interventions needed to focus on psychological (as opposed to physical) wellbeing of staff; be person-directed (versus work directed); and provide primarily emotional rather than cognitive/clinical support (thus for example excluding mortality/morbidity meetings which aim to provide lessons in terms of cognitive errors or systems issues). Although Rounds are a 'group' (rather than individual) intervention we chose not to limit comparative interventions by this characteristic, due to the importance of reflection and/or disclosure as a key potential mechanism in Rounds that is shared by other interventions that are not group-based. Potential comparative interventions were identified through published reviews of psychological/emotional support interventions for healthcare staff[30-33] and through consultation with steering and advisory group members (with expertise in Rounds/wellbeing interventions in healthcare). A total of 11 interventions were scoped: Action Learning Sets; After Action Reviews; Balint Groups; Caregiver Support

For peer review only - http://bmjopen.bmj.com/site/about/guidelines.xhtml

Programme; Clinical Supervision; Critical Incident Stress Debriefing; Mindfulness-Based Stress Reduction; Peer Supported Storytelling; Psychosocial Intervention Training; Reflective Practice Groups; Resilience Training.

Comparative analysis to Schwartz Rounds

The composite definition of Rounds was disaggregated into its individual descriptive features which were extracted into a table, together with the features that were 'not' part of Rounds. Further clarification was added for some descriptive features to ensure clarity of meaning (e.g. "reflection" became "provides an explicit opportunity for reflection"). The description of each comparative intervention was then reviewed by the research team and assessed in relation to whether or not it also provided each of the key features of Rounds. The face validity of the comparison between Rounds and other interventions was confirmed with study advisory and steering groups (with expertise in Rounds/healthcare staff wellbeing interventions).

Patient Involvement

We actively involved patients through membership of the Project Steering Group (PSG) which included two PPI representatives (Havi Carel, Christine Chapman) who had previously provided input to the original funding application. The PSG provided oversight to all aspects of the study, and alongside other group members our PPI representatives and Rounds staff members advised on design, inclusion of comparative interventions, and commented on the findings.

For peer review only - http://bmjopen.bmj.com/site/about/guidelines.xhtml

Results

Key features of Rounds

Forty-three documents/sources were included in the review of descriptions of Rounds (Table 1) which allowed development of the definition.[24,25,34-74]. The majority (n=33) were non-empirical publications (e.g. commentaries, descriptive reports of a single Round). The thematic synthesis resulted in the production of the composite definition (supplementary file 1), a summary version is provided in Table 1.

Evidence base for Rounds: results from the systematic review

Twelve empirical evaluations of Rounds were included (Table 2) arising from ten studies (four in the USA, six in the UK). Most were mixed methods evaluations, typically comprising attenders completing evaluation forms post-Round attendance, followed by interviews or focus groups (n=5), one mixed method study comprised case studies (observation/interviews) together with descriptive analysis of evaluation forms[75] and one that used both quantitative and qualitative methods to analyse evaluation forms.[76] Two were quantitative studies, and one qualitative study. Only one study included non-attenders[66] (Table 2).

Overall quality of the evidence-base was assessed to be low/moderate. Most studies had study designs prone to risk of bias (e.g. cross-sectional), used non-validated questionnaires (typically self-report views/satisfaction with Rounds and impact of attendance), and none of the quantitative evaluations had control group (non-attender) comparisons. Little information was provided on the samples/sampling frames in quantitative studies (e.g. in relation to breadth of professional group representation or role in Rounds), nor were findings analysed

For peer review only - http://bmjopen.bmj.com/site/about/guidelines.xhtml

or presented in relation to such factors. In two studies that did report the characteristics of their quantitative sample, most were female and of white ethnicity, and nurses predominated (but neither study reported the seniority of nurse).[24,47] Findings from these studies included that Rounds are highly valued by attenders (though represented a small proportion of total staff). Most studies reported positive impact on 'self' (e.g. improved wellbeing, coping)[24-25,44,47,49,51,59,66,70,75-76] and impact on patients (increased compassion, empathy),[24-25,51,59,66,70,75-76] and colleagues (improved teamwork, compassion/empathy).[24-25,44,47,50-51,59,66,75-76] Six studies provide evidence of wider institutional impacts from interviews with attenders[24-25,44,51,66,75-76] (Table 2). Three of the included studies were evaluations of Rounds adapted for educational purposes;[39,50,70] all reporting that Rounds were felt to be useful and that students gained knowledge/understanding about the emotional side of providing patient care.

Comparative interventions: results from the scoping reviews

Electronic searches for the 11 comparative interventions yielded a total of 1725 papers, of which 146 were included (ranging between one and 64 across interventions, Table 1, see supplementary file 3 for included references). A number of publications (n=253) were not obtainable due to being published in sources that no longer existed or not available through institutional subscription and internet searches. The largest evidence base was for clinical supervision (n=64) followed by Balint groups (n=26). Half of the studies were quantitative (n=74: RCT, observational, quasi experimental), 41 were qualitative (mixed designs, interviews, focus groups), 22 were mixed-methods, and nine were secondary studies (literature reviews). The literature was international with the majority of studies from the USA and the UK; other countries represented included Canada, Australia, Finland, Norway,

For peer review only - http://bmjopen.bmj.com/site/about/guidelines.xhtml

Sweden, Croatia, Spain, Italy, Israel and South Africa. There was a distinct lack of studies from Asia, although that may be a reflection of the English language limit.

For most interventions, high-quality evidence was sparse. Populations for many of the interventions lacked diversity across health professions and settings, with many mostly nursing-focused. The aims of studies varied widely, with a few aimed at assessing efficacy or effectiveness but most were small-scale exploratory descriptive studies. The content and format of interventions (fidelity) was in most cases widely heterogeneous (and/or lacked detail), and consequently synthesis of findings is problematic. Most of the quantitative evaluations across all interventions relied upon weaker study designs (e.g. cross-sectional studies, post-intervention evaluations, lacking control comparisons), used non-probability sampling, had small samples likely to be underpowered, and used non-validated outcomes measures. Many qualitative studies also lacked clear reporting of aspects of rigour (e.g. limited reporting of member checking, deviant cases, reflexivity or evidence of data saturation). A summary of the evidence base for each intervention is provided in Supplementary file 2.

Synthesis

Most interventions presented evidence in relation to all three categories of outcomes ("self", "others" and "organisation"), though evidence for Resilience training, Mindfulness-based stress reduction and Reflective practice groups lacked inclusion of organisational outcomes. All of the interventions had evidence of positive benefits to self (e.g. raised self-awareness, resilience, job satisfaction, empowerment, or overall wellbeing), and most provided some evidence of positive benefits to 'others'. Impact on patients included fostering of better provider-service user relationships, communication with and/or attitudes towards patients;

For peer review only - http://bmjopen.bmj.com/site/about/guidelines.xhtml

and improved patient-centredness, knowledge of patients' suffering and empathy. Impact on colleagues, included associations with better teamwork, peer support and knowledge/understanding of colleagues.

At organisational-level, there was evidence from some interventions of association with improved practice, for example, reductions in unnecessary prescriptions, increased uptake of psychosocial support (Balint groups), reduction in task and coordination errors and increased uptake of post-fall huddles (After action reviews). Two interventions provided evidence of a positive impact on the workforce, including providing opportunities for mentoring and advice (Action learning sets) and improved staff retention (Clinical supervision).

Schwartz vs alternative interventions: comparative features

In comparison to the other interventions, Rounds offer a unique organisation-wide 'all-staff' forum to share stories about the emotional impact of providing patient care (Table 3). Whilst many of the other interventions expect 'open, honest communication' as a key feature, and provide an explicit opportunity for reflection, none are open to all staff (e.g. clinical and non-clinical, voluntary attendance), and many are not ongoing programmes but instead are one-off training courses or events. Some of the training interventions (e.g. mindfulness-based stress reduction, or resilience training) are multidisciplinary in training attendance, but conduct/practice of the intervention occurs subsequently and is individual, compared to Schwartz (and other interventions such as Balint Groups) where learning and practice take place simultaneously in group settings.

Other key aspects in which Rounds are distinct from the comparative interventions relate to what Rounds are intentionally 'not' meant to be. In particular, discussions within Rounds should not 'problem solve' in order to avoid focus on the clinical decision-making in a patient case, whereas problem solving/action planning are key features of many of the other

For peer review only - http://bmjopen.bmj.com/site/about/guidelines.xhtml

interventions (e.g. Action learning sets, After action reviews, Critical incident stress debriefing). Most of the comparative interventions also offered flexibility in format, compared to Rounds which require a contractual license (with stipulated conditions) obtained via the Schwartz Centre for Compassionate Care (USA) or Point of Care Foundation (UK).

Arguably the closest types of interventions to Rounds are Balint groups (though rooted in uni-disciplinary primary care - physicians only- with closed membership), and Reflective practice groups (again generally closed membership and can be uni-disciplinary). In particular, both are ongoing group programmes in which challenging/rewarding experiences about delivering patient care are shared and discussions are facilitated, and both provide the opportunity to give and/or receive peer support in safe and confidential environments. However, neither offers an organisation-wide opportunity for staff to attend, and both would have an expectation that members/attenders would contribute, whereas in Rounds attenders can choose to be silent listeners. Clinical supervision can also provide an opportunity to reflect on the emotional and ethical challenges of care without problem-solving/action planning –but unlike Rounds this usually occurs in a one to one situation, not group, and requires those being supervised to verbally contribute.

Discussion

Our work revealed a rich portfolio of available interventions to support staff with the emotional challenges of providing healthcare, each designed with different audiences and uses in mind. The evidence base regarding the effectiveness of these in the main remains weak, and more should be done to examine these more systematically. The studies reviewed here show some evidence of impact at different levels, and future work should seek to unpick which interventions work best, under which conditions and for which participants. To our knowledge this is the first comparative review of staff wellbeing interventions.

For peer review only - http://bmjopen.bmj.com/site/about/guidelines.xhtml

Given the high rates of work-related stress and mental health issues amongst healthcare staff it is not acceptable for employers not to act, despite the weak evidence base for most approaches and interventions currently. Some staff groups have clinical supervision for example as an integral part of their work (mental health nurses; midwives; psychologists and social workers) whereas most doctors and nurses do not, and such staff often have little or no support with the emotional, social and ethical challenges of their work. Non-clinical staff – who may have much contact with patients and the events they encounter - are even more neglected in relation to the impact of delivering patient care on them. Selection of interventions should be based on a strategic approach that incorporates needs assessment, implementation of interventions/approaches and policies, and monitoring and review to determine the impact of these and refine/revise as necessary. There is a need for a range of approaches, not a one size fits all and our work does not suggest an either/ or approach for individual interventions. Rounds should not be seen as a replacement for or instead of clinical supervision (or other support/interventions) but could be offered to staff in addition. Organisation-wide interventions are important to tackle workplace environmental/cultural factors that impact on wellbeing; to change attitudes and cultural norms around staff needing support as well as changing conversations in organisations around empathy, compassion and the support required to deliver these. Involving all employees may improve co-worker and supervisor support, which in turn can facilitate the development of a supportive workplace environment that reduces stress by improving attitudes and behaviours.[77] Compared to other interventions reviewed here, Rounds offer a unique organisation-wide "all staff" forum to reflect on the emotional impact of providing patient care, offering opportunities for staff to reflect, whether or not they choose to disclose/contribute to discussions, and accruing

For peer review only - http://bmjopen.bmj.com/site/about/guidelines.xhtml

evidence suggests they may have many benefits to individuals, others (colleagues, patients) as well as wider organisational impacts.[26]

Schwartz Rounds were originally conceived to meet a very specific identified need in healthcare: to support healthcare providers to be compassionate to patients through giving them insight into their own thoughts, feelings and behaviours.[26] In the UK the reasons given for adoption has been more about staff wellbeing, in line with evidence linking quality of patient care and experience with staff wellbeing.[13, 23] Unlike many of the other interventions, they have a structured format, and are specifically not intended to be 'problem-solving'. In doing so they provide a 'counter-cultural' space that differs from the protocol-driven, outcome-orientated healthcare environment that values emotional stoicism: "Good Rounds shift an organisation and its workers away from their default position of urgent action, reaction and problem solving to an hour of stillness and slowness".[78,p41]

Workforce interventions are often complex in nature, with many components and aims. Their evaluation is thereby challenging, particularly with regard to attributing any changes to outcomes to the intervention as opposed to other causes within the organisation/system. The challenge of conducting a robust evaluations of organisation-wide interventions may be one explanation as to why such evidence is so sparse,[22] and for why there is instead a predominance of evidence regarding individually targeted interventions such as mindfulness-based stress reduction. The application of new methodologies to address these challenges, such as realist evaluation, could enable a more robust understanding of how and why interventions work (or don't work), and has recently been applied in the first UK national evaluation of Schwartz Rounds.[26]

For peer review only - http://bmjopen.bmj.com/site/about/guidelines.xhtml

Limitations

The focus of this review on the evidence within healthcare staff meant that wider evidence for some interventions, beyond healthcare, was not considered. Also, the scoping methods applied to the comparable interventions inevitably means that some relevant evidence may have been omitted, though systematic electronic searching and consultation with experts aimed to minimise this risk. The rapid uptake of Rounds in the UK and need to contextualise them within staff wellbeing interventions, informed the design of this review. It was thereby a review that compared other interventions to the key features of Rounds, and did not thereby compare the key features of all the other interventions to each other, apart from by describing and synthesising their origins and evidence base.

Conclusion

Given the time and resources already committed to the interventions considered here, it is important to determine how best to identify the core features of effectiveness to optimise benefit for individual staff members and organisations as a whole. This work has now been undertaken for Schwartz Rounds using a realist-informed methodology that has identified the contextual factors that influence how and for whom Schwartz Rounds work, resulting in an organisational guide giving practical guidance and recommendations for organisations to maximise the effectiveness of Rounds in their organisations.[26,79] The application of similar methodologies for other interventions such as clinical supervision and Balint groups may further help ensure optimal outcomes. A systems approach as opposed to an individual approach to tackling staff wellbeing, in order to improve patient care, is required, comprising effective interventions for assessing and improving the wellbeing of healthcare staff.

For peer review only - http://bmjopen.bmj.com/site/about/guidelines.xhtml

Funding:

This work was supported by the National Institute for Health Research Health Services and Delivery Research Programme (project number 13/07/49).

Disclaimer:

The views and opinions expressed therein are those of the authors and do not necessarily reflect those of the of the NHS, the NIHR, MRC, CCF, NETSCC, the HS&DR programme or the Department of Health.

Author contributions:

CT and JM conceived the original idea and designed the study; AX designed the search strategies and performed the database searches; CT, AX, ML and ER performed internet searches; screened titles and abstracts independently; extracted data and assessed quality for three interventions each; CT, JM, ML and ER created the composite definition of Rounds; All authors contributed to the overall synthesis and comparative analysis; CT drafted the manuscript; AX, ML, ER and JM revised critically the manuscript. All the authors approved the final version of the manuscript. We also thank Michelle Hope (MH) for her contribution to the analysis of definitions of Schwartz Rounds.

Acknowledgements: We thank the following members of our project advisory group and steering groups for their guidance and comments throughout the study.

Advisory Group: Joanna Goodrich, Barbara Wren, Jenny Firth-Cozens, Michael West, John Pepper, Glenn Robert.

Steering Group: Dean Fathers, Sharon Fleming, Nicola Mackintosh, Rachel Massey-Chase, Katherine Hopkins, Jude Bayly, Havi Carel, Christine Chapman, Elisabeth Buggins, Jennifer Gardner, Ruth Harris, and Lisl Klein.

For peer review only - http://bmjopen.bmj.com/site/about/guidelines.xhtml

Competing interests: Jill Maben reports that she was a member of an advisory group from 2006 to 2009, advising on the development of the Point of Care project at The King's Fund, and a member of the Point of Care Foundation Board 2013–14; she stepped down as board member at the start of the evaluation. All other authors declare no conflicts of interest.

Data sharing statement: No additional data are available

For peer review only

For peer review only - http://bmjopen.bmj.com/site/about/guidelines.xhtml

References

1. NHS (2018) https://www.nhs.uk/NHSEngland/thenhs/about/Pages/overview.aspx. Last accessed 15/2/18
2. Europa Eurostat (2018) http://ec.europa.eu/eurostat/statistics-explained/index.php/Healthcare_personnel_statistics_-_physicians. Last accessed 15/2/18.
3. Europa Eurostat (2018) http://ec.europa.eu/eurostat/statistics-explained/index.php/Healthcare_personnel_statistics_-_nursing_and_caring_professionals Last accessed 15/2/18.
4. Taylor C., Graham J., Potts H., Candy J., Richards M. and Ramirez A. Changes in mental health of UK hospital consultants since the mid-1990s Lancet. 2005; 366:742-44
5. Wall T.D., Bolton R.I., Borrill C.S., Carter A.J., Golya D.A., Hardy G.E., et al. Minor psychiatric disorders in NHS Trust employees: occupational and gender differences. British Journal of Psychiatry. 1997; 171:519-23.
6. Maslach C., Jackson S.E. Burnout in health professionals: a social psychological analysis In: Sanders GS, Suls J, editors. Social Psychology of Health and Illness. Hillsdale NJ: Lawrence Erlbaum Associates; 1982.
7. Arigoni, F., Bovier, P.A., Mermillod, B., Waltz, P., Sappino, A.-P. Prevalence of burnout among Swiss cancer clinicians, paediatricians and general practitioners: who are most at risk?. *Support Care Cancer*. 2009;17:75–81.
8. Trufelli, D., Bensi, C., Garcia, J. Burnout in cancer professionals: a systematic review and meta-analysis. *Eur J Cancer Care*. 2008;6:31.
9. Bressi, C., Manenti, S., Porcellana, M. et al, Haemato-oncology and burnout: an Italian survey. *Br J Cancer*. 2008;98:1046–1052.
10. Harvey, S.B., Laird, B., Henderson, M., Hotopf, M. The mental health of health care professionals: a review for the Department of Health. Department of Health, London; 2009
11. Quality Watch (2018) http://www.qualitywatch.org.uk/indicator/nhs-staff-sickness-absence# Last accessed 15/2/18.
12. Taylor C., Graham J., Potts H., Candy J., Richards M., Ramirez A. Impact of hospital consultants' poor mental health on patient care. British Journal of Psychiatry. 2007; 190:268-269
13. Maben J., Peccei R., Adams M., Robert G., Richardson A., Murrells T. and Morrow E. Patients' experiences of care and the influence of staff motivation, affect and well-being. Final report NIHR Service Delivery and Organisation programme; 2012. http://www.netscc.ac.uk/hsdr/files/project/SDO_FR_08-1819-213_V01.pdf
14. Maben J., Adams M., Peccei R., Murrells T., Robert G. Poppets and parcels: the links between staff experience of work and acutely ill older people's experience of hospital care. International Journal of Older People's Nursing. 2012;7(2):83-94
15. Raleigh V., Hussey D., Seccombe I. Do associations between staff and inpatient feedback have the potential for improving patient experience? An analysis of surveys in NHS acute trusts in England. Quality and safety in health care; 2009. 18:347-54.
16. Australian Government (2018) Healthy workers healthy futures initiative. http://www.healthyworkers.gov.au/ Last accessed 15.2.18.
17. American Nurses Association (2018) http://www.healthynursehealthynation.org Last accessed 15.2.18
18. Royal College of Physicians (2015)Work and Wellbeing in the NHS: why staff health matters to patient care. https://www.rcpsych.ac.uk/pdf/RCP-%20WorkWellbeingNHS.pdf 12th October 2015. Last accessed 15.2.18.
19. Rao AS, Bhutani G, Dosanjh N, Clarke J, Hughes JH, Easton S, Van Laar D, Cohen-Tovee E (2016) Psychological Wellbeing and Resilience: Resetting the Balance http://www.bps.org.uk/system/files/Public%20files/ar_v10_resetting_the_balance_160516.docx.pdf 2016 Last accessed 15.2.18.

For peer review only - http://bmjopen.bmj.com/site/about/guidelines.xhtml

20. The Kings Fund (2015) Staff Engagement: six building blocks for harnessing the creativity and enthusiasm of NHS staff. https://www.kingsfund.org.uk/sites/default/files/field/field_publication_file/staff-engagement-feb-2015.pdf Last accessed 15.2.18.
21. NHS Wales (2017) Staff Psychological Wellbeing Policy http://www.wales.nhs.uk/sitesplus/documents/862/340-PsychologicalWellbeingPolicy-ext31.12.17.pdf Last accessed 15.2.18.
22. NICE (2009) Mental wellbeing at work. Public Health Guidance (PH22) https://www.nice.org.uk/guidance/ph22 Last accessed 15.2.18.
23. Robert G, Philippou J, Leamy M, Reynolds E, Ross S, Bennett L, Taylor C, Shuldham C, & Maben J (2017) Exploring the adoption of Schwartz Center Rounds as an organisational innovation to improve staff wellbeing in England, 2009-2015. *BMJ Open*, 7(1): 1-10.
24. Lown BA, Manning CF The Schwartz Center Rounds: Evaluation of an Interdisciplinary Approach to Enhancing Patient-Centered Communication, Teamwork, and Provider Support Acad Med. 2010; 85:1073–1081.
25. Goodrich J (2012) Supporting hospital staff to provide compassionate care: Do Schwartz Center Rounds work in English hospitals? *JRSM*, **105**: 117–122. DOI 10.1258/jrsm.2011.110183.
26. Maben J, Taylor C, Dawson, J, Leamy M, McCarthy I, Reynolds E, Ross S, Shuldham, C, Bennett L, Foot C (2018) A realist informed mixed methods evaluation of Schwartz Center Rounds® in England. First Look Summary available from: https://www.journalslibrary.nihr.ac.uk/programmes/hsdr/130749/#/ Last accessed 15.2.18.
27. Jones CEL, Maben J, Jack RH, et al. A systematic review of barriers to early presentation and diagnosis with breast cancer among black women. BMJ Open 2014; 4:e004076. doi:10.1136/bmjopen-2013-004076
28. O'Cathain A, Murphy E, Nicholl J (2008) The quality of mixed methods studies in health services research. J Health Serv Res Policy 13(2):92-8. doi: 10.1258/jhsrp.2007.007074
29. Arksey, H. and O'Malley, L. (2005) Scoping studies: towards a methodological framework. *International Journal of Social Research Methodology*, 8(1):19-32.
30. Ruotsalainen J, Serra C, Marine A, Verbeek J. Systematic review of interventions for reducing occupational stress in health care workers. *Scand J Work Environ Health* 2008;**34**:169-78.
31. Boorman S. *NHS Health and Wellbeing Review*. London: Department of Health; 2009.
32. Ruotsalainen JH, Verbeek JH, Mariné A, Serra C. Preventing occupational stress in healthcare workers. *Cochrane Database of Systematic Reviews* 2015.
33. Harvey SB, Laird B, Henderson M, Hotopf M. The mental health of health care professionals: A review for the Department of Health. In. London; 2009.
34. Anon (2008) A place to Heal (2008) https://www.nurse.com/blog/2008/09/08/a-place-to-heal/ (Last accessed 15/2/18).
35. Barnard P (2013) Schwartz Rounds: Librarian Roles and Opportunities. *Journal of Hospital Librarianship*, 13:1, 71-78, DOI: 10.1080/15323269.2013.743368.
36. Baverstock AC, Finlay FO (2015) Maintaining compassion and preventing compassion fatigue: a practical guide *Arch Dis Child Educ Pract Ed* **0**:1–5. doi:10.1136/archdischild-2015-308582.
37. Berry M, Chaggar R (2014) Introducing Schwartz Center Rounds® to Watford General Hospital – challenging the status quo. *Anaesthesia*, **69**(Suppl. 4): 46 (no.72) Abstract Only.
38. Booth M (2014) Schwartz Center Rounds – a moment to reflect at St Wilfrid's Hospice eHospice: available from: http://www.ehospice.com/uk/Default/tabid/10697/ArticleId/11798 (last accessed 15/2/18).
39. Corless IB, Michel TH, Nicholas M, Jameson D, Purtilo R, Angelique D (2009) Educating Health Professions Students About the Issues Involved in Communicating Effectively: A Novel Approach *Journal of Nursing Education*, **48**(7): 367-373.
40. Cornwell J, Goodrich J (2010) Supporting staff to deliver compassionate care using Schwartz Center rounds – a UK pilot. *Nursing Times* **106**(5):10-12.

For peer review only - http://bmjopen.bmj.com/site/about/guidelines.xhtml

41. Davis C (2011) Forum to help staff deal with day-to-day stress. *Nursing Management* **18**(4): 18–21.
42. Dean E (2013) Time for Reflection. *Nursing Standard* **27**(48): 61.
43. Dedier J, Penson R, Williams W, Lynch T (1999) Race, Ethnicity, and the Patient-Caregiver Relationship. *The Oncologist* **4:**325-331 [included to represent the many publications with the same definition from the Schwartz Centre, Massachusetts General Hospital, Boston USA].
44. Deppoliti DI, Côté-Arsenault D, Myers G, Barry J, Randolph C, Tanner B (2015) Evaluating Schwartz Center Rounds® in an urban hospital center. *Journal of Health Organization and Management*, **29**(7): 973 – 987.
45. Foster S (2015) Schwartz Rounds: our experience. *British Journal of Nursing* **24**(5):309.
46. Gannon C (2014) Contrasting Schwartz Rounds with clinical ethics: three perspectives on their potential to impact on end-of-life care. *Nursing Ethics* **21**(5): 621-626.
47. George M (2016) Stress in NHS staff triggers defensive inward-focussing and an associated loss of connection with colleagues: this is reversed by Schwartz Rounds. *Journal of Compassionate Health Care* **3**:9.
48. Gibson S (2008) Schwartz Center Rounds: Focusing on the Patient-Caregiver Relationship. *Health Progress*. **Nov-Dec 2008**: 40-43.
49. Gishen F, Wood M (2015) Using Schwartz Center Rounds to change hospice culture: does it work? *BMJ Supportive & Palliative Care,* **5**(1): 108 10.1136/bmjspcare-2014-000838.17. Abstract Only.
50. Gishen F Whitman S, Gill D, Barker R, Walker S. (2016) Schwartz Centre Rounds: a new initiative in the undergraduate curriculum—what do medical students think? *BMC Medical Education* **16**:246.
51. Goodrich J (2011) Schwartz Center Rounds: Evaluation of the UK Pilots. The Kings Fund: London. Available from: https://www.kingsfund.org.uk/publications/schwartz-center-rounds-pilot-evaluation *(last accessed 15/2/18).*
52. Goodrich J (2014) Compassionate care and Schwartz Rounds: The nature of the work - Acknowledging it is hard. *Nurse Education Today*, **34**: 1185-87.
53. Hargreaves R (2015) "The value of Schwartz Rounds should not be underestimated" (comment) *Nursing Times* **111**(14):11.
54. Johal J (2015) Kindling Kindness for Compassionate Disaster Management. *PLOS Currents: Disasters* Available from: http://currents.plos.org/disasters/article/kindling-kindness-for-compassionate-disaster-management/ (last accessed 15/2/18).
55. Justice S, Rawles J, Brown J, Szczepanska S (2013) Will introducing 'Schwartz rounds' help foster a culture of compassionate care in the NHS? *Nursing Standard*, **27**(5)2: 26-27.
56. Kaplan, K (Powerpoint presentation): Leading Important Conversations in Healthcare: The Schwartz Center Rounds http://c.ymcdn.com/sites/www.odnetwork.org/resource/resmgr/2011_conf_ppts/g3_-_leading_important_conve.pdf (accessed 15/2/18).
57. Lee KJ, Forbes ML, Lukasiewicz GJ, Williams T, Sheets A, Fischer K, Niedner MF (2015) Promoting staff resilience in the pediatric intensive care unit. *Am J Crit Care*;**24**(5) 422-430 10.4037/ajcc2015720.
58. Lima J, Cavaliere R, Porensky E (2014) Closing the gap: Bereavement support for families, caregivers and medical staff. *Neuro-Oncology* **16**:178–183 doi:10.1093/neuonc/nou269.
59. Manning CF, Acker M, Houseman L, Pressman E, Goodman IF (2008) Schwartz Center Rounds: Evaluation Report (executive summary). Goodman Research Group Inc: Boston USA. Available from: http://www.theschwartzcenter.org/media/PTXAAE65CHR5UU4.pdf (Last accessed 15/2/18).
60. Moore CD, McCole Phillips J (2009) In These Rounds, Health-Care Professionals Heal Themselves. *Journal of Social Work in End-Of-Life & Palliative Care*, **5**(3-4): 116-125 DOI: 10.1080/15524250903555064.

For peer review only - http://bmjopen.bmj.com/site/about/guidelines.xhtml

61. Mudge B Stanzler M (2012) Schwartz Center Rounds® and Magnet®: Enhancing patient- and family-centered care American Nurse Today, 7(1) available from: https://www.americannursetoday.com/schwartz-center-rounds-and-magnet-enhancing-patient-and-family-centered-care/ Last accessed 1/3/17.
62. Mullick A, Wright A, Watmore-Eve J, Flatley M (2013) Supporting hospice staff: the introduction of Schwartz Center Rounds to a UK hospice setting. *European Journal of Palliative Care*, **20**(2): 62-65.
63. Pepper JR, Jaggar SI, Mason MJ, Finney SJ, Dusmet M (2012). Schwartz Rounds: reviving compassion in modern healthcare. *JRSM* **105:** 94–95.
64. Raphael-Grimm T (2008) Fostering compassionate patient care as an integrated, multidisciplinary outcome: Lessons learned from facilitating Schwartz Rounds. *International Journal for Human Caring*, 12(3):97.
65. Raso R (2009) Improving practice with Schwartz rounds; the DNP degree. *Nursing Management.* August 2009: 56.
66. Reed E, Cullen A, Gannon C, Knight A, Todd J (2015) Use of Schwartz Centre Rounds in a UK hospice: Findings from a longitudinal evaluation. *J. Interprof. Care* **29**:365–366.
67. Rees-Adams D, Hughes L (2014) Introducing Schwartz Center Rounds into a Welsh NHS Health Board: six months in – a reflection on the process and experience to date. *Clinical Psychology Forum* **263**: 26-27 (*Special Issue of the British Psychological Society Clinical Psychology Forum on The Francis Report).*
68. Ryan B (2013) Schwartz Center Rounds are one way to boost compassion (Letter). *Nursing Standard* **27**(50):31.
69. Sanghavi D (2006) What makes for a compassionate patient-caregiver relationship? *Joint Commission Journal on Quality and Patient Safety* **32**(5):283-292.
70. Shield RER, Tong I, Tomas M, Besdine RW (2011) Teaching communication and compassionate care skills: An innovative curriculum for pre-clerkship medical students. *Medical Teacher* **33**: e408–e416.
71. Smyth M (2011) End of shift: goodbye to baby https://www.nurse.com/blog/2011/07/11/end-of-shift-goodbye-to-baby-2/ (last accessed 1/3/17).
72. Thompson A (2013) How Schwartz rounds can be used to combat compassion fatigue. *Nursing Management* 20(4):16–20.
73. Whitehead P (2015) The Impact of Schwartz Center Rounds on Moral Distress in a Large Healthcare System. *Journal of pain and symptom management* **49**(2):461. Abstract Only.
74. Wren B (2014) Schwartz Rounds: An intervention with potential to simultaneously improve staff experience and organisational culture. *Clinical Psychology Forum* **263** (*Special Issue of the British Psychological Society Clinical Psychology Forum on The Francis Report).*
75. Farr M, Barker R (2017) Can staff be supported to deliver compassionate care through implementing Schwartz Rounds in community and mental health services? *Qualitative Health Research*, 27(11): 1652-63.
76. Chadwick RJ, Muncer SJ, Hannon BC, Goodrich J, Cornwell J (2016) Support for compassionate care: Quantitative and qualitative evaluation of Schwartz Center Rounds in an acute general hospital. *JRSM Open*, 7(7): doi: 10.1177/2054270416648043
77. Babin BJ, Boles JS (1996) The effects of perceived co-worker involvement and supervisor support on service provider role stress, performance and job satisfaction. Journal of Retailing 72(1): p57.
78. Wren B. Schwartz Rounds: An intervention with potential to simultaneously improve staff experience and organisational culture. (Special Issue of the British Psychological Society Clinical Psychology Forum on The Francis Report). *Clinical Psychology Forum* 2014;**263**.
79. Maben J. Leamy M. Taylor C. Reynolds E. Shuldham C. Dawson J. McCarthy I. Ross S. Bennett L. (2018) *An Organisational guide: Understanding, implementing and sustaining Schwartz Rounds*® Copyright © 2018 King's College London Available from: https://www.surrey.ac.uk/SchwartzEvaluation

For peer review only - http://bmjopen.bmj.com/site/about/guidelines.xhtml

Table 1 Search results for each intervention

Intervention	Description of intervention	Database search result	Total excluded (duplicates; not eligible; full paper not available)	Papers from experts/ internet search	Total number included for review
Schwartz Center Rounds (Rounds)	Regular (usually monthly) open forum for staff in all positions. Organised and managed by a steering group, championed by a senior clinician, and facilitated – usually by a senior doctor and psychosocial practitioner. Last for one hour, with food provided. Multiple perspectives on a theme, scenario or patient case are briefly presented by a pre-arranged and pre-prepared panel and then opened to the audience for group reflection and discussion. Focus on non-clinical aspects (psychosocial, ethical, emotional issues) surrounding the patient-caregiver relationship. [*See Supplementary File 1 for a full definition*]	41	0	2	**43** **(33 non – empirical; 10 empirical)**
Action learning sets (ALS)	Based on the concept of learning by reflection on (or reviewing) an experience, ALS usually contains 4-6 members (peers), with (or without) a "set advisor" to facilitate the process. ALS tend to be held intermittently, over a fixed programme cycle, and most participants contract with the facilitator for an agreed length of time. They are often closed groups. The set is not a team, as the focus is on actions of individuals, rather than shared work objectives.	83	70 (8; 36; 26)	1	**14**
After action reviews (AAR)	AARs are facilitated meetings, led by a senior member of staff, which aim to encourage active reflection on performance following a specific event. An AAR is a one-off meeting post-event and includes those who were involved with the event. The focus is on gaps in performance, and what could be done differently to enhance the outcome. AARs generally last about 30 minutes.	76	74 (9; 64; 1)	0	**2**
Balint groups	Balint groups meet every 1-4 weeks for 1-3 years. In the group, typically a doctor presents a troubling patient incident while the group listens. The goal of the presentation is to understand the issue from both the patient's and doctor's perspectives. The presentation can last about ten minutes, after which group members can ask clarifying questions. When all questions are exhausted, the group is invited to imagine themselves in the roles of the doctor and the patient.	384	358 (170;151;37)	0	**26**
Caregiver support programme (CSP)	Originally developed for mental health/learning disability care homes, CSP is described as a theory-based social support intervention aimed at increasing exchanges of social support and decreasing negative social interaction. It consists of six 4-5-hour group training sessions (ten managers, ten direct-care staff and two facilitators) conducted over a nine week period. Strategies for improvement are drawn from the participants, based on their own experiences.	84	83 (10, 71, 2)	2	**3**

For peer review only - http://bmjopen.bmj.com/site/about/guidelines.xhtml

Intervention	Description of intervention	Database search result	Total excluded (duplicates; not eligible; full paper not available)	Papers from experts/ internet search	Total number included for review
Clinical supervision	Clinical supervision originated in psychotherapy but also adopted by other disciplines, e.g. psychology/nursing. Process described as identifying a key issue, describing and defining it, undertaking a critical analysis, examining solutions, formulating an action plan, implementation and evaluation. It can take five different forms: one-to-one with expert from same discipline; one-to-one with supervisor from different discipline; one-to-one with colleague of similar expertise; supervision between groups of colleagues working together, and network supervision between people who do not usually work together.	307	252<br>(42, 160, 50)	9	**64**
Critical incident stress debriefing (CISD)	In its original form, CISD is a single-issue debriefing session in a group context, led by an external team, following a traumatic event. CISD has seven phases: *introduction, fact, thought, reaction, symptom, teaching,* and *re-entry*. The debriefing session lasts for approximately 1.5-3 hours and takes place 24-72 hours after the traumatic event. The debriefing team is made up of a leader, a co-leader and a support, who work in conjunction to support the participants and to allow them to feel safe.	388	386<br>(62; 248; 76)	0	**2**
Mindfulness-based stress reduction (MBSR)	The central principle of MBSR is mindfulness - being focused on and aware of the present moment with a non-judging attitude of acceptance. The original training module is eight weeks long with weekly sessions of 2.5 hours each. There is a seven hour session which takes place between weeks six and seven, and participants are asked to complete 45 minutes of daily formal mindful practice. They are taught a variety of mindful meditative practices, and there are group discussions about the application of these practices.	127	110<br>(13; 72; 25)	0	**17**
Peer-supported storytelling	Narrative storytelling is the act of an individual recounting verbally to one or more people a plausible account of an event, or series of events, possessing narrative truth for the teller. The story is arranged in a time sequence with plot, characters, context, intentionality, and perspective taking, possibly including the teller's actions, thoughts and feelings.	4	3<br>(3; 0; 0)	0	**1**
Psychosocial intervention training	Psychosocial intervention training involves cognitive behavioural approaches for managing symptoms, understanding symptom-related behaviour, relationship formation and helping service users to cope with symptoms. Teaching sessions are supplemented by small group supervision. Students are required to provide brief case study presentations about service users they are working with and receive feedback. Early courses were developed for nurses but quickly became multidisciplinary.	37	35<br>(6; 25; 4)	1	**3**
Reflective practice groups (RPG)	RPGs are facilitated groups of about ten healthcare professionals or students in which participants share and explore professional, clinical, ethical, and personal insights arising from their clinical work or training. RPGs are ongoing, convening regularly with each group lasting for about one hour. Discussion topics can either be raised by the facilitator or by the participants. The discussion is meant to be supportive as well as challenging, encouraging consideration of alternative viewpoints.	91	83<br>(8; 73; 2)	0	**8**

For peer review only - http://bmjopen.bmj.com/site/about/guidelines.xhtml

Intervention	Description of intervention	Database search result	Total excluded (duplicates; not eligible; full paper not available)	Papers from experts/ internet search	Total number included for review
Resilience training	Resilience training is in part based on CBT theories and in its original form is a manualised intervention comprising 18 hours of workshops. The key characteristics include delivery to groups of practitioners who support one another and facilitated by an expert in personal and professional transition supervision. University of Pennsylvania well-known example consists of: learning ways to challenge unrealistic negative beliefs, strengthening problem solving, adopting assertiveness and negotiation skills, improving ability to deal with strong feelings, and learning how to tackle procrastination through use of decision-making and action-planning tools.	144	138<br>(36, 72, 30)	0	**6**
**TOTAL**		**1725**	**1592**<br>**(367; 972; 253)**	**13**	**146**

For peer review only - http://bmjopen.bmj.com/site/about/guidelines.xhtml

Table 2: Schwartz Rounds empirical evaluations: data extracted from included papers (Short legend: Schwartz Rounds Included Studies)

Authors	Setting	Aims/purpose	Design/methodology	Measures	Main findings	Quality
Corless et al (2009)[39]	Educational<br><br>USA	Development, implementation and evaluation of Educational Rounds for interdisciplinary graduate students to help them learn empathy, self-reflection and moral courage	Quantitative post-Round evaluation survey<br><br>Graduate students. Over a 4-years comprising 11 Rounds (n=329 individual evaluations)	Survey included 7 statements about Rounds (according to agreement on a 5-point Likert scale) plus an overall rating of the quality of the Round they are evaluating.	**Overall**<br>High support and satisfaction with Rounds (e.g. 86% rated Rounds as excellent or exceptional).<br>67% stated intention to attend future Rounds (range 57-93% for individual Rounds). Lowest intention from a Round presented by lab scientists. Highlighted importance of topic to encourage attendance.	Low/Moderate (lack of clarity e.g. sampling, measures, not all data presented).
Manning et al (2008)[59]<br>Lown et al (2010)[24]	Hospitals<br><br>USA	To assess the impact of Rounds e.g. changes in attendees behaviours and beliefs about patient care, teamwork, stress and personal support	Mixed method evaluations<br><br>- Retrospective survey of 256/413 (62%) attenders at 6 experienced Rounds sites (offering Rounds for 3+years) plus 44 interviews with providers, Rounds leaders, facilitators and hospital administrators.<br><br>- Prospective pre-post web-based survey of 222/399 (56%). Rounds attenders from 10 hospitals newly implementing Rounds (had held 7 or more Rounds)	Study-specific (non-standardised) - some adapted from published measures) Likert scale measures to investigate:<br>1) insights into psychosocial and emotional aspects of clinical care on patient interactions (15 items)<br>2) teamwork (9 items)<br>3) support for providers (number of items not mentioned)	**Overall**<br>Found "dose" effect: more rounds attended, more impact they have.<br>**Self**<br>Attendance at Rounds associated with decreased stress and improved ability to cope with psychosocial demands/emotional difficulties at work.<br>**Others**<br>Rounds attendance led to increased patient interaction and teamwork scores. Interviews highlighted benefits including: getting to know colleagues and putting themselves in their shoes, and an improved sense of connection/shared purpose.<br>**Organisation**<br>Both samples (51% retrospective; 40% prospective) reported changes in practices/polices including: culture change (dialogue that doesn't happened elsewhere); focus on patient-centred care; practice changes (e.g. increased/earlier palliative care use)	**Quantitative:** Moderate (lack of control group of non-attenders and used non-standardised measures).<br>**Qualitative:** Moderate (limited reporting of theoretical underpinnings and strategies to improve rigour e.g. deviant case analysis)<br>**Mixed methods:** low (limited reporting of mixed methods rationale/features)
Goodrich (2011)[51]<br>Goodrich (2012)[25]	Two hospitals<br><br>UK	Pilot study to evaluate introduction of Rounds to UK in 2 hospital sites.	Mixed methods evaluation over 2 year period:<br><br>1) Pre-post pilot surveys<br><br>2) Evaluation forms from each Round (quality,	Used same questionnaires as Lown et al. (2010)[24]	**Overall**<br>Majority (86% site A, 78% site B) rated rounds as excellent/good.<br>**Self**<br>- Increases (pre-post) in:<br>- confidence in handling sensitive issues<br>- beliefs in the importance of empathy	**Quantitative:** Moderate (limitations in measures used and lack of control group)<br><br>**Qualitative:** Moderate (low reporting of

For peer review only - http://bmjopen.bmj.com/site/about/guidelines.xhtml

Authors	Setting	Aims/purpose	Design/methodology	Measures	Main findings	Quality
			logistics, demographics, plans to attend future Rounds). Each site held 10 Rounds (n=301 attenders site A, 74% completed evaluation form; n=949 at site B, 69% completed evaluation form).<br><br>3) Qualitative interviews: Experience of attenders, steering group, panellist, facilitators (n=23). Second interview at end with n=13.		- confidence in handling non-clinical aspects of care<br>Also reported feeling less stressed and less isolated in their work.<br>Interview findings: increased compassion, reduced stress<br>**Others**<br>Increases (pre-post) in:<br>- actual empathy with patients<br>- openness to expressing thoughts, questions and feelings about patient care with colleagues<br>Interview findings: greater respect/empathy for colleagues, better teamwork/collaboration<br>**Organisation**<br>Interview findings:<br>- Board/senior support important<br>- Wider impacts: reduced hierarchy, help build shared values/support strategic vision.	strategies to improve rigour and theoretical underpinnings)<br><br>**Mixed methods:** low (limited reporting of mixed methods rationale/features)
Reed et al (2015)[66]	Hospice<br><br>UK	Evaluate the impact of Rounds on staff and the organisation	Longitudinal mixed methods evaluation (1 year): survey and focus groups<br><br>Exit survey: 398/535 (74%) attendees)<br><br>4 interprofessional focus groups (n=33, including attendees, non-attendees and presenters)	5-point Likert scale assessing: Topic relevance, knowledge gained, impact on individual, facilitation and working relationships<br><br>(*Similar questions to Lown & Manning, 2010*[24]*/Goodrich, 2011*[51]).	**Overall**<br>78% rated Rounds as excellent or exceptional<br>**Self**<br>- Focus groups:<br>- Validation of experiences<br>- Honesty, openness and vulnerability allowed others to see person on human level<br>**Others**<br>87% gained insight into how others think/feel in caring for patients<br>- Focus groups: fostered understanding of importance of non-clinical staff contribution<br>- BUT non-attenders felt responsibility to smooth running of hospice and felt they contributed to wider team without needing to hear stark realities of care/work.<br>**Organisation**<br>- Focus groups: more connected, shared purpose	**Quantitative**: Moderate, (non-validated measures and lack of control group)<br>**Qualitative:** Moderate (lack of elements of rigour in qualitative component e.g. reflexivity, contradictory/ deviant cases other than non-attenders)<br>**Mixed methods:** low (limited reporting of mixed methods rationale/features)
Deppoliti et al (2015)[44]	Hospital<br><br>USA	- Learn why people attend Rounds<br>- Understand what is gained from the experience<br>- Identify key	Qualitative: 4 focus groups (n=27) and 3 telephone interviews<br><br>Purposive sampling of attenders by steering group to represent those that were active contributors and	N/A	**Overall**<br>Rounds viewed as beneficial.<br>**Self**<br>- impact on behaviour/attitudes "think differently"<br>**Others**<br>- exposing emotions (increased appreciation, awareness and sensitivity of what others in the healthcare team experience)	Qualitative: High

For peer review only - http://bmjopen.bmj.com/site/about/guidelines.xhtml

Authors	Setting	Aims/purpose	Design/methodology	Measures	Main findings	Quality
		elements to use in measuring effectiveness	included range of roles/professions and frequency (low and high attenders).		- walking in another's shoes (empathic awareness)<br>**Organisation**<br>- culture change (strong message that staff matter; values/beliefs/ norms evolved positively; not about productivity; improved teamwork due to level playing field).<br>**Other findings:**<br>- inequality of topics (some topics more than others lead to increased learning, growth)<br>- influence of rules and boundaries (spoken/unspoken rules about what is acceptable to share)<br>*Suggested improvements:*<br>- providing list of upcoming topics so staff can plan attendance<br>- providing anonymised method to contribute (eg Qs on cards)	
George (2016)[47]	Hospital<br><br>UK	To examine the impact of Schwartz Rounds on staff wellbeing and patient care	Mixed methods<br><br>Interviews with staff (nurses and HCAs) about stress (n=11, 10 were female, 10 were white British)<br><br>Key themes extracted using grounded theory ➔ development of a new measure administered at the beginning and end of 2 Rounds (n=55 forms completed) Mostly female, white and only 2 were over 59yrs old.	The Organisational Response to Emotions Scale (ORES) (investigator-designed): 9 scales<br><br>Analysis controlled for whether it was first ever Round, length of time in role, session attended.	**Self**<br>- Emotional Labour: significantly reduced in staff where pre-round was their first round.<br>- Self-reflection increased pre-post<br>Compared SCR attenders with 10/11 interviewees who also completed ORES (did not attend Rounds). Found non-attenders had higher burnout and emotional labour, and more negative appraisal of organisation.<br>**Others**<br>- More negative appraisal of line manager	**Quantitative:** Moderate (small biased sample, lack of control group, measure based on limited staff group input (nurses/HCA only).<br><br>**Qualitative:** Moderate (limited reporting of elements of rigour e.g. audit trail, theoretical saturation)<br><br>**Mixed methods:** high (good reporting of mixed methods)
Shield et al (2011)[70]	Medical school<br><br>USA	To improve communication skills, they designed "Schwartz Communication Sessions" Aimed to provide medical students with the	Quantitative: evaluation form (both quant and qual/content analysis).<br><br>Sampling is unclear (a sample of 92, 99 and 94 are reported) but report having evaluation forms from 66-95% of students for all three sessions (71-80% for two	Not specified (but appear similar to Lown & Manning, 2010)[24]	**Overall**<br>93% of faculty and 83% of students rated the sessions as good, excellent or exceptional<br>**Self**<br>80% of students and 96% of faculty believe students gained knowledge that will help them care for patients<br>**Others**<br>75% of students and 96% of faculty believe the sessions will help students communicate better with patients and family members	Moderate (lack of clarity regarding sampling/sample and measures)

For peer review only - http://bmjopen.bmj.com/site/about/guidelines.xhtml

Authors	Setting	Aims/purpose	Design/methodology	Measures	Main findings	Quality
		rationale and proficiency for effective communication.	sessions) and Faculty members (n=24) response rate 42-92%			
Gishen et al (2016)[50]	Medical school<br><br>UK	Examine the potential of Rounds within the undergraduate curriculum	Mixed method evaluation<br><br>2 student-focused Rounds were piloted at a medical school (1 Round each for year 5 and 6 students)<br><br>Evaluation questionnaire immediately following the Rounds:<br>258/334 (77%) year 5 students attended the Round and 247 (94%) responded.<br><br>180/343 (52%) year 6 students attended the Round and 126 (70%) responded.<br><br>Focus group (n=7 year 5 students) to explore student views on the Round	Feedback form from the Point of Care Foundation; plus free text comments. Questions either Yes/No or 5-point Likert rating scale (1= poor to 5 = exceptional)	**Overall**<br>Mean student ratings of a session were 3.5/5 (year 5) and 3.3/5 (year 6)<br>- 81% agreed/strongly agreed the presentation of cases was helpful<br>- 80% would attend a future Round<br>- 64% agreed Rounds should be integrated into the curriculum<br><br>Focus group finding:<br>Feelings about the Round (response to round, size of audience- large inhibiting, positive comparison to current reflective practice; post event peer discussions)<br>**Self**<br>- 69% year 5 vs 87% year 6 students were worried about compassion fatigue or burnout<br>- 92% agreed/strongly agreed that they appreciated hearing stories demonstrating human side of medicine<br>- Focus group finding: Psychological aspects of SCR (psychological pressures of medicine, how session encouraged positive processing of emotion, sharing personal stories between health professionals).<br>**Others**<br>82% agreed/strongly agreed that attending Round gave insight into how others feel/think about caring for patients	Quantitative:<br>Moderate due to convenience sampling approach and lack of control group<br><br>Qualitative:<br>Moderate due to limited reporting of measures taken to enhance rigour<br><br>Low for mixed methods reporting.
Farr and Baker (2016)[75]	Community and mental health services<br><br>UK	To investigate how Schwartz Rounds are implemented and how they support staff in mental health and community services	Mixed methods: Realist evaluation involving 3 case studies including 22 interviews, 5 observations and descriptive analysis of evaluation sheets as secondary data.<br><br>Key themes extracted using	Topic guides developed from a previous evaluation of Schwartz Rounds Goodrich, 2012[4]<br><br>Process data collected through	**Overall**<br>Rounds had the potential for successful implementation in these settings.<br>**Self**<br>More mindful of emotional impact of work<br>**Others**<br>Positive impact on relationships with patients and other staff (e.g. better communication, increased trust)<br>**Organisation**	**Qualitative:** Moderate (methods for realist methods and analysis lack clarity; e.g. unclear presentation of Context-Mechanism-Outcome configurations)<br>**Quantitative**: Low (descriptive data on

For peer review only

For peer review only - http://bmjopen.bmj.com/site/about/guidelines.xhtml

Authors	Setting	Aims/purpose	Design/methodology	Measures	Main findings	Quality
			framework analysis, then used to track context, mechanism outcome configurations	evaluation sheets i.e attendance, types of participants and perspectives	Rounds can positively influence wider culture of the organisation (e.g. prompt introduction of other forms of staff support)<br>**Other findings:**<br>Facilitators and barriers to implementation are identified including that strong leadership is a crucial element in success	attenders only; lack of clarity on analysis of evaluation sheet data and integration with interview data).<br>**Mixed methods**: Low (limited reporting of mixed methods rationale/features)
Chadwick et al (2016)[76]	Hospital<br><br>UK	To assess the perceived impact of Schwartz Rounds™ on hospital staff over a 3 year period	Mixed methods analysis of evaluation forms<br><br>795 participant evaluation forms analysed quantitatively and qualitatively<br><br>Key themes extracted using thematic analysis<br>Quantitative (scale) data analysed descriptively and inferentially (e.g. for differences in scores for different items/scales).	Standard evaluation form, eight statements rated on five-point scale and free text comments.	**Overall**<br>All aspects of Rounds highly rated by staff<br>**Self**<br>Improved reflection on own experiences<br>**Others**<br>Improved insight and understanding of others and their roles and support within the organization<br>Most agreed that attended helped caring for patients and work with colleagues<br>**Organisation**<br>Reducing the effect of 'silo working'<br>Staff were given a space where powerful emotions were accepted and responded to constructively<br>Need to make Rounds more accessible to wider cross section of staff<br>**Other findings:**<br>- No significant differences between disciplines/staff groups in survey ratings	**Qualitative:** High<br>**Quantitative:** Moderate (statistical methods unclear; lack of account of impact of sample size on outcomes of analysis)<br>**Mixed Methods:** Not a mixed methods study, just in analysis of evaluation data therefore criteria n/a

For peer review only - http://bmjopen.bmj.com/site/about/guidelines.xhtml

Table 3 Features of Rounds compared/contrasted with comparative interventions (Short Legend: Comparative Analysis)

		**Intervention (see footnote for full label)**										
	**Feature of Rounds**	**ALS**	**AAR**	**Balint**	**Care**	**Super**	**CISD**	**Mind**	**Story**	**Psych**	**Refl**	**Resil**
1	Share challenging/rewarding experiences about delivering patient care	May Not	No	Yes	May Not	Yes	Yes	No	May Not	Yes	Yes	May Not
2	Focus on psychosocial and emotional issues of patient-caregiver relationships	May Not	No	Yes	May Not	May Not	May Not	No	May Not	May Not	May Not	May Not
3	Provides an explicit opportunity for reflection	Yes	Yes	Yes	May Not	Yes	Yes	Yes	Yes	Yes	Yes	May Not
4	Open, honest communication	Yes	Yes	Yes	Yes	Yes	Yes	Yes	Yes	Yes	Yes	May Not
5	Provides an opportunity to give and/or receive peer support	Yes	May Not	Yes	Yes	Yes (if group)	Yes	Yes	Yes	No	Yes	Yes
6	Telling and hearing stories related by theme, scenario or patient case	No	Yes	No	May Not	No	No	No	Yes	No	No	No
7	Ongoing programme (vs. one-off)	No	No	Yes	No	Yes	No	No	No	No	Yes	No
8	Time-fixed session (vs. flexible length/unspecified)	No	No	Yes	Yes	Yes	No	Yes	No	Yes	No	Yes
9	Planned provision of food/refreshments	No	No	No	No	No	No	No	No	No	No	No
10	Open to all/any clinical and non-clinical staff	No	Yes	No	No	No	No	No	No	No	No	May Not
11	All levels of staff/no hierarchy	Yes	Yes	No	Yes	No	No	Yes	Yes	Yes	No	Yes
12	Open group membership (vs. closed/invited members only)	No	No	No	No	No	No	No	No	No	No	No
13	Multidisciplinary	May Not	Yes	May Not	Yes	No	May Not	Yes	May Not	Yes	No	May Not
14	Pre-prepared/rehearsed stories or focus	Yes	No	No	No	No	No	No	Yes	No	No	No
15	Facilitated discussions	May Not	Yes	Yes	Yes	Yes	Yes	Yes	No	Yes	Yes	Yes
16	Panel presenters tell stories giving their perspectives on a theme, scenario or patient case	No	No	No	No	No	No	No	No	No	No	No

For peer review only

For peer review only - http://bmjopen.bmj.com/site/about/guidelines.xhtml

	Feature of Rounds	Intervention (see footnote for full label)										
		**ALS**	**AAR**	**Balint**	**Care**	**Super**	**CISD**	**Mind**	**Story**	**Psych**	**Refl**	**Resil**
17	Group intervention	Yes	Yes	Yes	Yes	May Not	Yes	Yes	No	Yes	Yes	Yes
18	Organisational support: senior doctor/clinician champions	May Not	Yes	Yes	Yes	Yes	May Not	No	No	No	No	No
19	Safe and confidential environment	Yes	Yes	Yes	Yes	Yes	Yes	Yes	Yes	Yes	Yes	Yes

Features that define what Rounds are 'not'		Intervention										
		**ALS**	**AAR**	**Balint**	**Care**	**Super**	**CISD**	**Mind**	**Story**	**Psych**	**Refl**	Resil
1	Problem-solving	Yes	Yes	Yes	Yes	Yes	May Not	No	No	Yes	No	Yes
2	Production of actionable outputs	Yes	Yes	No	No	May Not	No	No	No	No	No	No
3	Flexibility in format (versus licenced/contract[1])	Yes	Yes	Yes	May Not	Yes	Yes	Yes	Yes	No	Yes	May Not
4	Focus on clinical aspects of patient care, their diagnosis or plan of care	May Not	May Not	No	May Not	May Not	May Not	No	May Not	Yes	May Not	May Not

*Manualised but adapted to experiences of group

ALS= Action Learning Sets; AAR= After Action Reviews; Balint= Balint Groups; Care=Caregiver Support Programme; Super=Clinical/Restorative Supervision; CISD= Critical Incident Stress Debriefing; Mind= Mindfulness-based stress reduction; Story=Peer supported story-telling; Psych= Psychosocial Intervention Training; Refl= Reflective Practice Groups; Resil=Resilience Training.

[1] Licenced/contract – fidelity to original intervention – i.e. one model/approaches or many, degree of flexibility offered.

For peer review only - http://bmjopen.bmj.com/site/about/guidelines.xhtml

Supplementary file 1: Composite definition of Rounds (key features shown in bold)

"Schwartz Rounds are the signature programme of the Schwartz Center for Compassionate Care. They provide a **regular** (usually monthly) **open forum** (drop-in rather than by invitation) for **multidisciplinary** clinical and non-clinical **staff at all positions** within the healthcare organisation to come together in an environment that is **safe and confidential**. They provide staff with a **level playing field** to **reflect** upon, explore and tell stories about the difficult, **challenging and rewarding experiences they face when delivering patient care**, and **receive the support of their colleagues**. Rounds are typically organised and managed by a steering group, **championed by a senior doctor/clinician**. They **last for one hour** and are often held during lunch periods (with **food provided**). They are a **group intervention** within which **multiple perspectives on a theme, scenario or patient case** (i.e. their stories) are briefly presented by a **pre-arranged and pre-prepared panel** and then opened to the audience for group reflection and discussion, usually **facilitated** by a senior doctor and psychosocial practitioner. The **focus is on the non-clinical aspects (e.g. psychosocial, ethical and emotional issues) surrounding the patient-caregiver relationship** – thereby addressing a wide range of important topics rarely discussed elsewhere - and the attendees are encouraged to be **open and honest** and reflect, discuss and explore their experiences thoughts and feelings. The interaction between the panellists and audience is felt to foster insight and support from colleagues, create a sense of working in a supportive environment and lead to improved relationships and communication within the hospital hierarchy, improved communication and teamwork between staff and patients and among staff, improved wellbeing of staff including enhanced resilience, improved compassionate care and ultimately impact on organisational culture."

Some sources explicitly articulated what Rounds were ***not***. This included: that aims were distinct from traditional clinical/grand rounds; that is stories and discussions should **not focus**

For peer review only

on the patient, their diagnosis or plan of care; that Rounds should not be used for **problem solving**; and that they are **not intended to produce actionable outputs**.

For peer review only

Supplementary File 2: Evidence base (in healthcare professionals) for Schwartz Rounds® and comparative interventions

Intervention	Healthcare settings	Number/type of papers included	Intervention fidelity between studies	Key Findings to:<br>Self/ Others/ Organisation	Overall strength of the evidence base*
**Schwartz Center Rounds®**	Hospitals, hospices, and educational settings (e.g. medical schools) – open to all staff of all grades (clinical and non-clinical).	10 papers (8 studies):<br>- 2 quantitative<br>- 1 qualitative<br>- 5 mixed methods	Yes as Rounds are licenced and have to be run according to a format subsequent to obtaining a licence.	**Self**<br>*Wellbeing:* reduced stress, emotional labour, and isolation at work<br>*Coping:* improved ability to cope with emotional difficulties at work; confidence in handling sensitive issues<br>*Self-reflection*: increased self-reflection/validation of experiences<br>*Knowledge*: students report increased knowledge/understanding about emotional side of providing patient care.<br><br>**Others**<br>*Patients*: increased compassion and empathy, increased interaction.<br>*Colleagues*: improved teamwork, communication, compassion/empathy<br><br>**Organisation**<br>*Access*: improved access to specific services (e.g palliative care)<br>*Culture change*: having dialogue that doesn't happen elsewhere; reinforced shared values and support strategic vision; reduced hierarchy	Quantitative: low/moderate due to cross-sectional design, non-validated measures, and lack of control (non-attender) data.<br>Qualitative: moderate due to limited reporting of theoretical underpinnings and strategies to improve rigour (e.g. deviant case analysis; reflexivity)<br>Mixed methods: Low

For peer review only

Intervention	Healthcare settings	Number/type of papers included	Intervention fidelity between studies	Key Findings to: Self/ Others/ Organisation	Overall strength of the evidence base*
**Action Learning Sets**	Pre/post registration education/training; primary care (e.g. online networks for GPs); acute and mental health services; palliative and continuing care; care homes and prisons.	14 papers:<br>- 6 quantitative<br>- 4 qualitative<br>4 mixed methods	Most hybrid interventions where ALS only one component. Group sizes between 4-8 members. Evaluation period 3months to a year, and 2- 6 sessions.	**Self**<br>*Empowerment:* foster greater psychological empowerment; sense of self-efficacy, self-esteem and confidence<br>*Awareness:* offer opportunity to explore self<br><br>**Others**<br>*Colleagues:* improved understanding and knowledge of colleagues; offers opportunity to share experiences and give/receive peer support<br><br>**Organisation**<br>*Workforce*: opportunities for mentoring and advice; satisfaction with the intervention being accessible and inclusive; possible limitations include having conflicting commitments and lack of group cohesiveness	Quantitative: Low due to lack of validated measures, and small sample sizes that were underpowered.<br><br>Qualitative: Moderate to high quality: some well-designed studies but others lacked detail on analytical strategy, limited transparency of findings (e.g. deviant cases not discussed, quotes not fitting themes and/or author interpretation)<br><br>Mixed methods: Low
**After Action Reviews (AAR)**	Acute settings	2 papers:<br>2 quantitative	Variability evident: 2 year evaluation of post-fall huddles vs. cross-sectional evaluation of one-off training	**Self**<br>*Empowerment:* improved confidence in dealing with difficult situations<br><br>**Others**<br>*Patients:* improved communication and listening skills	Quantitative: Low to moderate – mostly non-experimental designs with convenience samples and non-validated (study-specific) outcome measures;

For peer review only

Intervention	Healthcare settings	Number/type of papers included	Intervention fidelity between studies	Key Findings to: Self/ Others/ Organisation	Overall strength of the evidence base*
			in AAR	**Organisation**<br>*Practice:* reduced task and coordination errors; increased uptake of post-fall huddles; positive impact on patient care	poor survey response.
**Balint groups**	Primary care (predominantly GPs), medical schools, acute settings	26 papers:<br>- 12 quantitative<br>- 6 qualitative<br>- 8 mixed methods	Principles applied consistently but some variations or hybrid-models (e.g. Balint-inspired roundtable groups)	**Self**<br>*Awareness:* raised personal awareness in doctors; helped medical students build a professional identity as doctors<br>*Resilience:* reduced stress; helped doctors and students resolve challenging situations in practice<br>*Job satisfaction*: improved job satisfaction in doctors<br><br>**Others**<br>*Patients:* foster positive attitudes towards 'difficult' patients; improve students' ability to relate with patients; improve patient-centredness<br>*Colleagues:* promote teamwork<br><br>**Organisation**<br>*Practice:* reduced unnecessary prescriptions; increased uptake of psychosocial support; higher patient satisfaction	Quantitative: Low – mostly observational studies that lack randomisation, use non-validated measurement tools, and inadequately control confounding variables.<br><br>Qualitative: Moderate – mostly reflective case studies, with loose adherence to qualitative designs and limited description of approaches to rigour.<br><br>Mixed methods: Low

For peer review only

Intervention	Healthcare settings	Number/type of papers included	Intervention fidelity between studies	Key Findings to: Self/ Others/ Organisation	Overall strength of the evidence base*
**Caregiver Support Program**	Mental health /learning disability homes	3 papers:<br>- 3 quantitative (all the same study)	No variability – has only been used and evaluated in one study.	**Self**<br>_Empowerment:_ measured ability to cope with common work problems and ability to influence decision making (non-significant improvement);<br>_Wellbeing:_ psychological wellbeing (non-significant improvement)<br><br>**Others**<br>_Colleagues:_ greater supervisor support, less undermining, greater praise and feedback<br><br>**Organisation**<br>_Workforce:_ No change in outcomes for managers (only direct-care staff); "train the trainers" element (to translate intervention to workplace) did not work	Quantitative: Moderate to low – the main limitations concern the sample (low response rates to surveys), method of analysis (did not use all the data, could have included people responding at only one time point) and measures (use of measures that were not well validated)
**Clinical and Restorative Supervision**	Nursing education leadership, and nursing generally (including mental health, paediatrics, addiction, elderly and dementia care, oncology, hospice nursing), and in emergency	64 papers:<br>9 Secondary studies (literature reviews)<br>55 Primary studies: - 22 quantitative<br>27 qualitative<br>- 6 mixed methods	Wide variability in every aspect. Duration ranged from 1 mth to 5 yrs (median 12 mths). Session length ranged from 1- 3hrs	**Self**<br>_Awareness_: improved knowledge and insights, professional awareness and development<br>_Empowerment:_ improved communication skills, job resources, professional efficacy and capacity for reflection<br>_Wellbeing_: reduced psychological distress; improved vitality; reduced stress; reduced burnout (lower emotional exhaustion and	Quantitative: Mostly Low/Moderate. Most are cross-sectional studies, with a few pre-post (mostly without control groups) and short-term follow-up. Only a few RCTs have been conducted and these are moderate-

For peer review only

Intervention	Healthcare settings	Number/type of papers included	Intervention fidelity between studies	Key Findings to: Self/ Others/ Organisation	Overall strength of the evidence base*
	departments.		(median 1.5hrs), and frequency from weekly to monthly. Most were group supervision, ranging from 2 to 12 supervisees. Treatment fidelity was rarely referred to.	depersonalisation); improved sense of security, belonging and encouragement<br>*Resilience:* improved rational coping<br>*Job stress/satisfaction*: reduced job stress; improved job satisfaction<br><br>**Others**<br>Patients: better knowledge of patients' suffering and how to take responsibility; improved individualised documentation in patient notes;<br>Colleagues: foster solidarity, sharing and reflecting<br><br>***Organisation***<br>*Practice:* Reduced positive and total symptoms (in patients with psychosis) reported by student nurses<br>*Workforce:* staff retention<br><br>Continuity and quality of supervision are key moderators of outcomes.	high quality.<br>Inadequate sample sizes and unvalidated/ unreliable measures in most (mostly self-report of supervisor/supervisee rather than impact on patient/care).<br><br>Qualitative: Low/Moderate.<br>Majority lack conceptual/theoretical bases; have inappropriate sampling and lack of transparency in methods/analysis.<br><br>Mixed methods: Low
**Critical Incidence Stress Debriefing**	Acute and community settings.	2 papers:<br>-2 quantitative	Fairly consistent approach to CISD following the Mitchell model	**Self**<br>*Wellbeing:* no significant impact on stress reduction, but significantly lower PTSD scores; feeling part of a group and realising they were not alone	Quantitative: Moderate –weaknesses or limited reporting of group allocation, sampling approach, consideration of

For peer review only

Intervention	Healthcare settings	Number/type of papers included	Intervention fidelity between studies	Key Findings to: Self/ Others/ Organisation	Overall strength of the evidence base*
			(1983). Details of the content of the intervention lacking. Consistency in approach between group facilitators questionable.	**Others**<br>*Colleagues:* opportunity for sharing experiences, and learning from others<br><br>**Organisation**<br>*Workforce:* potentially negative impacts included increased intrusive thoughts	confounding variables, and psychometric values of instruments used.
**Mindfulness-based Stress Reduction**	Acute and community settings, with doctors and nurses (qualified and in-training) and a range of allied health professions.	17 papers:<br>-15 quantitative<br>-1 qualitative<br>-1 mixed method	Mostly informed by Kabat-Zinn (1990).<br>One study combined face-to-face and telephone delivery of sessions; and five studies reported on a shortened version.<br>Content of sessions was similar across studies, but	**Self**<br>*Wellbeing:* improved relaxation and mood. Some mixed results in relation to burnout, stress, anxiety, distress, and satisfaction with life<br>*Resilience:* improved self-compassion, self-care, acceptance and coping skills<br>**Others**<br>*Colleagues:* opportunity for peer support	Quantitative: Moderate due to non-probability sampling, bias from confounding and lack of power calculations.<br>Qualitative: Moderate due to limited reporting of approaches to enhancing rigour (e.g. member checking, deviant cases, reflexivity, data saturation).<br>Mixed Methods: Low

For peer review only

Intervention	Healthcare settings	Number/type of papers included	Intervention fidelity between studies	Key Findings to: Self/ Others/ Organisation	Overall strength of the evidence base*
			many lacked detail to judge fidelity.		
**Peer-supported storytelling**	Paediatric 17 papers<br>-15 quantitative<br>-1 qualitative<br>-1 mixed method<br>nurses who had experienced grief	1 paper:<br>- mixed methods	Only one study consisted of 3 self-selected dyads (6 nurses). Each dyad met biweekly for 2 months for brief informal storytelling sessions (each member taking a turn telling a story and listening at each session, mean length 17mins).	**Self**<br>*Wellbeing:* positive impact on grief<br>*Resilience:* positive impact on meaning-making, making sense of, and identifying benefit in, their experiences<br><br>**Others**<br>*Colleagues:* opportunity to receive and provide support during sessions<br><br>**Organisation**<br>*Workforce:* significant positive correlation between number of 'special' patient deaths during career and impact of sessions on grief	Quantitative: Low due to small (n=6), self-selected sample and no comparison group<br><br>Qualitative: Moderate due to descriptive approach to analysis, lacking in theoretical saturation<br><br>Mixed methods: Low
**Psychosocial intervention training**	Mental health settings in the UK	3 papers:<br>-3 quantitative	Consistent programme; delivered weekly over 4-5 months. Variable	**Self**<br>*Wellbeing:* improved burnout (emotional exhaustion, depersonalisation and personal achievement)<br>*Empowerment:* improved knowledge of, and attitude towards, mental illness and	Quantitative: Moderate –all 3 studies were quasi-experimental, utilising non-probability sampling, but lacking a power

Intervention	Healthcare settings	Number/type of papers included	Intervention fidelity between studies	Key Findings to: Self/ Others/ Organisation	Overall strength of the evidence base*
			duration between 4 to 8 months.	psychosocial approaches	calculation and not clearly accounting for confounding variables.
**Reflective Practice Groups**	Clinical psychology, psychotherapy, nursing, medicine, midwifery and radiology.	8 papers:<br>-3 quantitative<br>-3 qualitative<br>-2 mixed methods	All described as facilitated groups that explore practice related issues. Can last between 45-90 minutes; held weekly, fortnightly or monthly; group sizes varied from six to 20 attendees, with one or two facilitators.	**Self**<br>*Awareness:* improved self-awareness and clinical insight<br>*Empowerment:* increased confidence and capacity for reflection; better understanding of psychological ideas<br>*Wellbeing:* increased ability to cope with stress<br><br>**Others**<br>*Colleagues:* opportunity for peer support, sharing experience and learning; improved communication skills<br>*Patients:* positive impact on empathy<br><br>**Organisation**<br>*Practice:* perceived improvements in the quality of care provided	Quantitative: Low – mostly weakened by the absence of probability sampling, lack of validated tools, missing baseline measurements and inadequate control for confounding variables.<br><br>Qualitative: Low – mostly weakened by absence of an overall design and inadequate attention to data saturation, sampling and researcher reflexivity.<br><br>Mixed methods: Low
**Resilience Training**	Medical students, intensive care unit nurses, general medicine, emergency service	6 papers:<br>- 6 quantitative (reporting on five studies)	Two studies (three papers) evaluated resilience training alone.	**Self**<br>*Wellbeing:* reduced depression, PTSD, stress and anxiety<br>*Resilience:* improved cognitive appraisal, control of stress; decreased negative	Quantitative: Moderate –mostly pilot studies to determine the feasibility, acceptability or prove

For peer review only

Intervention	Healthcare settings	Number/type of papers included	Intervention fidelity between studies	Key Findings to: Self/ Others/ Organisation	Overall strength of the evidence base*
	personnel.		The remainder combined resilience training with other interventions. Interventions varied from 10-12 weeks of regular training, to 40min one-off.	emotion and expression inhibition	the concept of the intervention. Limitations included only comparing within groups (despite having control group), ambiguous details regarding sampling and/or lacking control group.

References:

Kabat-Zinn, J. (1990). *Full catastrophe living: Using the wisdom of your body and mind to face stress, pain and illness.* New York: Delacorte

Mitchell, J. T. (1983). When disaster strikes: The critical incident stress debriefing process. Journal of Emergency Medical Services, **8**(1):36-9.

For peer review only

Supplementary file 3: References for included papers from the scoping reviews of comparable interventions

Action Learning Sets

Baillie L, Bromley B, Walker M, Jones R, Mhlanga F (2014) Implementing service improvement projects within pre-registration nursing education: A multi-method case study evaluation. *Nurse Education in Practice*, 14(1): 62-68.

Booth A, Sutton A, Falzon L (2003) Working together: supporting projects through action Learning, *Health Information and Libraries Journal*, 20: **2**25–231.

Bourner T & Frost P (1996) Experiencing action learning. *Employee Counselling Today*, 8(6): 11–18.

Burgess H & Carpenter J (2008) Building capacity and capability for evaluating the outcomes of social work education (the OSWE project): Creating a culture change. *Social Work Education*, 27(8): 898-912.

Currie K, Biggam J, Palmer J, Corcoran T (2012) Participants' engagement with and reactions to the use of on-line action learning sets to support advanced nursing role development. *Nurse Education Today* 32: 267–272.

Douglas S & Machin T (2004) A model for setting up interdisciplinary collaborative working in groups: lessons from an experience of action learning. *Journal of psychiatric and mental health nursing*, 11: 189–193.

Eisen S, Sukhani S, Brightwell,A, Stoneham S, Long A (2013) Peer mentoring: evaluation of a novel programme in paediatrics, *Arch. Dis. Child.* 0:1–5.

Lamont S, Brunero S, Russell R (2010) An exploratory evaluation of an action learning set within a mental health service. *Nurse Education in Practice*, 10: 298-302.

Leggat S, Balding C and Anderson J. (2011) Empowering health-care managers in Australia: an action learning approach, *Health Services Management Research*, 24: 196–202.

Machin A & Pearson P (2014) Action learning sets in a nursing and midwifery practice learning context: A realistic evaluation. *Nurse Education in Practice* 14: 410-416.

Mann R, Ball K, Watson G (2011) Mentoring for NHS general practitioners: a prospective pilot study of an action learning approach, *Educ Prim Care,* 22(4): 235-240.

Moore L (2007) Partnerships and work-based learning: an evaluation of an opportunity to pioneer new ways to care for the older people in the community, *Assessment & Evaluation in Higher Education*, 32(1): 61-77.

Phillips S, Tapping J, Ooms A, Marks-Maran D, Godden R (2013) A preceptorship programme for health visitors and school nurses: a pilot study *Community Practitioner*, 86(1): 18-22.

Rivas K & Murray S (2010) Our shared experience of implementing action learning sets in an acute clinical nursing setting: approach taken and lessons learned. *Contemporary Nurse*, 35 (2): 182-7.

After Action Reviews

For peer review only

Reiter-Palmon R, Kennel V, Allen JA, Jones KJ, Skinner AM (2015) Naturalistic decision making in after-action review meetings: The implementation of and learning from post-fall huddles *Journal of Occupational and Organizational Psychology*, 88(2): 322–340.

Walker J, Andrews S, Grewcock D, Halligan A (2012) Life in the slow lane: making hospitals safer, slowly but surely. *Journal of the Royal Society of Medicine*, 105: 283–287.

Balint Groups

Abeni MS, Magni M, Conte S, Mangiacavalli L, Pochintesta G, Vicenzi VV, Ferretti A, Pompa F, Cocito C, Klersy, Corso C (2014) Psychological care of caregivers, nurses and physicians: A study of a new approach. *Cancer Medicine* 3(1): 101-110.

Adams KE, O'Reilly M, Romm J, James K (2006) Effect of Balint training on resident professionalism. *American Journal of Obstetrics & Gynecology* 195(5): 1431-1437.

Airagnes GSM, Consoli O, De Morlhon AM, Galliot C, Lemogne, Jaury P (2014) Appropriate training based on Balint groups can improve the empathic abilities of medical students: A preliminary study. *Journal of psychosomatic research* 76(5): 426-429.

Dahlgren MA, Almquist A, Krook J (2000) Physiotherapists in Balint group training. *Physiotherapy Research International* 5(2): 85-95.

Dokter HJ, Duivenvoorden HJ, Verhage HF (1986) Changes in the attitude of general practitioners as a result of participation in a Balint group. *Family practice* 3(3): 155-163.

Fitzgerald G & Hunter MD (2003) Organising and evaluating a Balint group for trainees in psychiatry. *Psychiatric Bulletin* 27(11): 434-436.

Johnson AH, Brock CD, Hamadeh G, Stock R (2001) The current status of Balint groups in US family practice residencies: A 10-year follow-up study, 1990-2000. *Family medicine* 33(9): 672-677.

Johnson AH, Brock CD, Hueston WJ (2003) Resident physicians who continue Balint training: a longitudinal study 1982-1999. *Family medicine* 35(6): 428-433.

Johnson AH, Nease DE, Milberg LC, Addison RB (2004) Essential characteristics of effective Balint group leadership. *Family medicine* 36(4): 253-259.

Kjeldmand D & Holmstrom I (2008) Balint groups as a means to increase job satisfaction and prevent burnout among general practitioners. *Annals of Family Medicine* 6(2): 138-145.

Kjeldmand D. and Holmstrom I (2010). Difficulties in Balint groups: a qualitative study of leaders' experiences. *British Journal of General Practice* 60(580): 808-814.

Kjeldmand D, Holmstrom I, Rosenqvist U (2004) Balint training makes GPs thrive better in their job. *Patient education and counseling* 55(2): 230-235.

Kjeldmand D, Holmstrom I, Rosenqvist U (2006) How patient-centred am I? A new method to measure physicians' patient-centredness. *Patient Education & Counseling* 62(1): 31-37.

Lee E & Kealy D (2014). Revisiting Balint's innovation: enhancing capacity in collaborative mental health care. *Journal of Interprofessional Care* 28(5): 466-470.

Leggett A (2012). Transcultural issues in the dynamics of a Balint clinical reflection group for community mental health workers. *Transcultural Psychiatry* 49(2): 366-376.

For peer review only

Levenstein S (1978). A report of three years' experience of a Balint group in Cape Town. *South African Medical Journal*, 54(3): 121-123.

Levenstein S. (1981). An undergraduate Balint group in Cape Town. *South African Medical Journal*. 59(18): 642-643.

Levenstein S (1982). An undergraduate Balint group in Cape Town - A follow-up report. *South African Medical Journal* 62(3): 89-90.

Margalit AP, Glick SM, Benbassat J, Cohen A (2004) Effect of a biopsychosocial approach on patient satisfaction and patterns of care. *Journal of General Internal Medicine* 19(5 Pt 2): 485-491.

Nielsen HG & Tulinius C (2009) Preventing burnout among general practitioners: is there a possible route? *Education for Primary Care* 20(5): 353-359.

Salander Pä, Sandström M (2014) A Balint-inspired reflective forum in oncology for medical residents: Main themes during seven years. *Patient Education & Counseling* 97(1): 47-51.

Samuel O (1989) How doctors learn in a Balint group. *Family practice* 6(2): 108-113.

Sekeres MA, Chernoff M, Lynch TJ, Kasendorf EI, Lasser DH, Greenberg DB (2003) The impact of a physician awareness group and the first year of training on hematology-oncology fellows. *Journal of Clinical Oncology* 21(19): 3676-3682.

Smith M & Anandarajah G (2007) Mutiny on the balint: balancing resident developmental needs with the balint process. *Family medicine* 39(7): 495-497.

Stojanovic-Spehar S, Blazekovic-Milakovic S, Matanic D (2004) Education about pharmacotherapy and psychotherapy of anxiety among primary care physicians in Croatia: Balint group approach, *Croatian medical journal* 45(5): 625-629.

Torppa MA, Makkonen E, Martenson C, Pitkala KH (2008) A qualitative analysis of student Balint groups in medical education: contexts and triggers of case presentations and discussion themes. *Patient Education & Counseling* 72(1): 5-11.

Yakeley J, Shoenberg P, Morris R, Sturgeon D, Majid S (2011) Psychodynamic approaches to teaching medical students about the doctor-patient relationship: Randomised controlled trial. *Psychiatrist* 35(8): 308-313.

Caregiver Support Program

Heaney C (1991) Enhancing Social Support at the Workplace: Assessing the Effects of the Caregiver Support Program *Health Educ Behav* 18(4); 477-494.

Heaney C, Price RH, Rafferty J (1995) Increasing Coping Resources at Work: A Field Experiment to Increase Social Support, Improve Work Team Functioning, and Enhance Employee Mental Health. *Journal of Organizational Behavior*, 16 (4): 335-352.

Heaney CA, Price RH, & Rafferty J (1995) The caregiver support program: An intervention to increase employee coping resources and enhance mental health. In L. R. Murphy, J. J. J. Hurrel, S. L. Sauter, & G. P. Keita (Eds.), *Job stress interventions* (pp. 93-108). Washington, DC: American Psychological Association.

Clinical and Restorative Supervision

Primary studies:

Arvidsson B, Lofgren H, Fridlund B (2001) Psychiatric nurses' conceptions of how a group supervision programme in nursing care influences their professional competence: a 4-year follow-up study. *Journal of Nursing Management*, 9(3): 161-171.

Ardvidsson B, Skarsater I, Oijervall J, Fridlund B (2008) Process-oriented group supervision implemented during nursing education: nurses' conceptions 1 year after their nursing degree. *Journal of Nursing Management*, 16: 868–875.

Ashmore R, Carver N, Clibbens N, Sheldon J (2012) Lecturers' accounts of facilitating clinical supervision groups within a pre-registration mental health nursing curriculum. *Nurse Education Today* 32: 224-228.

Begat IBE, Severinsson, EI (2001) Nurses' reflections on episodes occurring during their provision of care-An interview study. *International Journal of Nursing Studies*, 38: 71-77.

Bogo M, Paterson J, Tufford L, King R (2011) Interprofessional clinical supervision in mental health and addiction: Toward identifying common elements. *The Clinical Supervisor*, 30:1, 124-140.

Bondas T (2010) Nursing leadership from the perspective of clinical group supervision: A paradoxical practice. *Journal of Nursing Management*, 18: 477-486.

Bradshaw T, Butterworth A, Mairs H (2007) Does structured clinical supervision during psychosocial intervention education enhance outcome for mental health nurses and the service users they work with? *Journal of Psychiatric and Mental Health Nursing*, 14: 4-12.

Brunero S, Lamont S (2012) The process, logistics and challenges of implementing clinical supervision in a generalist tertiary referral hospital. *Scand J Caring Sci*; 26; 186–193.

Buus N, Angel S, Traynor M, Gonge H (2010) Psychiatric hospital nursing staff's experiences of participating in group-based clinical supervision: An interview study. *Issues in Mental Health Nursing*, 31:10, 654-661.

Danielsson A, Sundin-Andersson C, Hov R, Athlin E (2009) Norwegian and Swedish preceptors' views of their role before and after taking part in a group supervision program. *Nurs Health Sci*, 11(2):107-113.

Davey B, Desousa C, Robinson S, Murrells T (2006) The policy--practice divide: Who has clinical supervision in nursing? *Journal of Research in Nursing*, 11(3): 237-248.

Davys AM & Beddoe L (2009) Interprofessional learning for supervision: 'taking the blinkers off'. *Learning in Health and Social Care*, 8(1): 58-69.

Edwards D, Burnard P, Hannigan B, Cooper L, Adams J, Juggessur T, Fothergil A, Coyle D (2006) Clinical supervision and burnout: The influence of clinical supervision for community mental health nurses. *Journal of Clinical Nursing* 15, 1007–1015.

Edwards D, Cooper L, Burnard P, Hannigan B, Adams J, Fothergill A, Coyle D (2005) Factors influencing the effectiveness of clinical supervision. *Journal of Psychiatric and Mental Health Nursing*, 12: 405-414.

For peer review only

Flackman B, Fagerberg I, Haggstrom E, Kihlgren A, Kihlgren M (2007) Despite shattered expectations a willingness to care for elders remains with education and clinical supervision. *Scand J Caring Sci*, 21: 379-389.

Gonge H & Buus N (2011) Model for investigating the benefits of clinical supervision in psychiatric nursing: A survey study. *International Journal of Mental Health Nursing*, 20: 102-111.

Gonge H & Buus N (2010) Individual and workplace factors that influence psychiatric nursing staff's participation in clinical supervision: a survey study and prospective longitudinal registration. *Issues in Mental Health Nursing*, 31:5,345-354.

Hallberg IR (1995) Clinical group supervision and supervised implementation of planned individualized care of severely demented people: effects on nurses, provision of the care, and patients. *Journal of Psychiatric and Mental Health Nursing*, 2: 111-114.

Hallberg IR (1994) Systematic clinical supervision in a child psychiatric ward: Satisfaction with nursing care, tedium, burnout, and the nurses' own report on the effects of it. *Archives of Psychiatric Nursing*, 8(1): 44-52.

Hall-Lord M, Theander K, Athlin E (2013) A clinical supervision model in bachelor nursing education -- Purpose, content and evaluation. *Nurse Education in Practice*, 13: 506-511.

Hansebo G & Kihlgren M. Nursing home care: changes after supervision. *Journal of Advanced Nursing* 45(3), 269–279.

Heaven C, Clegg J, Maguire P (2006) Transfer of communication skills training from workshop to workplace: The impact of clinical supervision. *Patient Education and Counseling* 60, 313–325.

Holmlund K, Lindgren B, Athlin E (2010) Group supervision for nursing students during their clinical placements: its content and meaning. *Journal of Nursing Management*, 18, 678–688.

Hyrkas K (2005) Clinical supervision, burnout, and job satisfaction among mental health and psychiatric nurses in Finland. *Issues in Mental Health Nursing*, 26:5, 531-556.

Hyrkas K, Appelqvist-Schmidlechner K, Haataja R (2006) Efficacy of clinical supervision: Influence on job satisfaction, burnout and quality of care. *Journal of Advanced Nursing*, 55(4): 521-535.

Jones A (2003) Some benefits experienced by hospice nurses from group clinical supervision. *European Journal of Cancer Care* 12, 224–232.

Jones 2001 Jones, A. Some experiences of professional practice and beneficial changes derived from clinical supervision by community Macmillan nurses. *European Journal of Cancer Care*, 10: 21-30.

Kelly B, Long A, McKenna H (2001) A survey of community mental health nurses' perceptions of clinical supervision in Northern Ireland. *Journal of Psychiatric and Mental Health Nursing*, 8: 33-44.

Kennedy TJT, Lingard, L, Baker, GR, Kitchen L, Regehr, G (2007) Clinical oversight: Conceptualizing the relationship between supervision and safety. *Society of General Internal Medicine*, 22:1080–1085.

Kenny A & Allenby A (2013) Implementing clinical supervision for Australian rural nurses. *Nurse Education in Practice*, 13: 165-169.

Kilcullen N (2007). An analysis of the experiences of clinical supervision on Registered Nurses undertaking MSc/graduate diploma in renal and urological nursing and on their clinical supervisors. *Journal of Clinical Nursing* 16, 1029–1038.

For peer review only

Knox S, Caperton W, Phelps D, Pruitt N (2014) A qualitative study of supervisees internal representations of supervisors. *Counselling Psychology Quarterly*, 27:4, 334-352.

Koivu A, Saarinen PI, Hyrkas K (2012) Who benefits from clinical supervision and how? The association between clinical supervision and the work-related well-being of female hospital nurses. *Journal of Clinical Nursing*, 21, 2567–2578.

Koivu A, Hyrka, SK, Saarinen PI (2011) Who attends clinical supervision? The uptake of clinical supervision by hospital nurses. *Journal of Nursing Management* 19, 69–79.

Koivu A, Saarinen PI, Hyrkas K (2012) Does clinical supervision promote medical–surgical nurses_ well-being at work? A quasi-experimental 4-year follow-up study *Journal of Nursing Management* 20, 401–413.

Lakeman R & Glasgow C (2009) Introducing peer-group clinical supervision: an action research project. *International Journal of Mental Health Nursing,* 18: 204-210.

Lantz I & Severinsson E (2001) The influence of focus group-oriented supervision on intensive care nurses' reflections on family members' needs. *Intensive and Critical Care Nursing*, 17: 128-137.

Lindgren B & Athlin E (2010) Nurse lecturers' perceptions of what baccalaureate nursing students could gain from clinical group supervision. *Nurse Education Today* 30:360-364.

Lindgren B, Brulin C, Holmlund K, Athlin E (2005) Nursing students' perception of group supervision during clinical training. *Journal of Clinical Nursing* 14, 822–829.

Magnusson A, Lutzen K, Severinsson E (2002) The influence of clinical supervision on ethical issues in home care of people with mental illness in Sweden. *Journal of Nursing Management*, 10: 37-45.

Malin NA (2000) Evaluating clinical supervision in community homes and teams serving adults with learning disabilities. *Journal of Advanced Nursing*, 31(3): 548-557.

O'Connell B, Ockerby CM, Johnson S, Smenda H, Bucknall TK (2011) Team clinical supervision in acute hospital wards: a feasibility study. *Western Journal of Nursing Research*, 35(3): 330-347.

Odling G, Danielson E, Jansson L (2001) Caregivers' descriptions of patients with advanced breast cancer in connection with supervision sessions in a surgical ward. *Cancer Nursing*, 24(1): 28-34.

Olofsson B (2005) Opening up: psychiatric nurses' experiences of participating in reflection groups focusing on the use of coercion. *Journal of Psychiatric and Mental Health Nursing*, 12: 259-67.

Olsson (1998) Systematic clinical supervision of home carers working in the care of demented people who are at home: structure content and effect as experienced by participants. *Journal of Nursing Management,* 6: 239-46.

Palsson M-BE, Hallberg IR, Norbert A (1994) Systematic clinical supervision and its effects for nurses handling demanding care situations: interviews with Swedish district nurses and hospital nurses in cancer care. *Cancer Nursing* 17(5): 385-394.

Pesut DJ & Williams CA (1990) The nature of clinical supervision in psychiatric nursing: a survey of clinical specialists. *Archives of Psychiatric Nursing* 4(3): 188-194.

Saarikoski M, Marrow C, Abreu W, Riklikiene O, O'zbicakc S (2007) Student nurses' experience of supervision and Mentorship in clinical practice: A cross cultural perspective. *Nurse Education in Practice*, 7, 407–415.

For peer review only

Severinsson EI, Hallberg IR (1996) Systematic clinical supervision, working milieu and influence over duties: the psychiatric nurse's viewpoint -a pilot study. *Int J. Nurs. Stud.*, 33(4): 394-406.

Sirola-Karvinen P, Hyrkas K (2008) Administrative clinical supervision as evaluated by the first-line managers in one health care organization district. *Journal of Nursing Management*, 16: 588-600.

Sloan G (1999) Good characteristics of a clinical supervisor: a community mental health nurse perspective. *Journal of Advanced Nursing*, 30(3), 713-722.

Walsh K, Nicholson J, Keough C, Pridham R, Kramer M, Jeffrey J (2003) Development of a group model of clinical supervision to meet the needs of a community mental health nursing team. *International Journal of Nursing Practice*, 9: 33-39.

White E, Butterworth T, Bishop V, Carson J, Jeacock J, Clements A (1998) Clinical supervision: insider reports of a private world. *Journal of Advanced Nursing* 28(1), 185-192.

White E & Winstanley J (2009) A randomised controlled trial of clinical supervision: selected findings from a novel Australian attempt to establish the evidence base for causal relationships with quality of care and patient outcomes, as an informed contribution to mental health nursing practice development. *Journal of Research in Nursing*, 15(2): 151-167.

Williams L & Irvine F (2009) How can the clinical supervisor role be facilitated in nursing: a phenomenological exploration. *Journal of Nursing Management* 17: 474-483.

Secondary studies (Literature reviews):

Brunero S & Stein-Parbury J (2008) The effectiveness of clinical supervision in nursing: an evidenced based literature review. *Australian Journal of Advanced Nursing*, 25(3):86-94.

Butterworth T, Bell L, Jackson C, Majda P (2008) Wicked spell or magic bullet? A review of the clinical supervision literature 2001–2007. *Nurse Education Today*, 28: 264-72.

Dawson M, Phillips B, Leggat S (2013) Clinical Supervision for Allied Health Professionals: A Systematic Review. *J Allied Health,* 42(2):65–73.

Ducat WH, Kumar S (2015) A systematic review of professional supervision experiences and effects for allied health practitioners working in non-metropolitan health care settings. *Journal of Multidisciplinary Healthcare*, 8:397-407.

Farnan JM, Petty LA, Georgitis E, Martin S, Chiu E, Prochaska M, Arora VM (2012) A Systematic Review: The Effect of Clinical Supervision on Patient and Residency Education Outcomes *Academic Medicine*, 87(4): 428-442.

Henshaw A-M, Clarke D, Long AF (2013) Midwives and supervisors of midwives' perceptions of the statutory supervision of midwifery within the United Kingdom: A systematic review. *Midwifery*, 29: 75–85.

Kilminster SM & Jolly BC (2000) Effective supervision in clinical practice settings: a literature review. *Medical Education*, 34:827-840.

Pearce P, Phillips B, Dawson M, Leggat SG (2013) Content of clinical supervision sessions for nurses and allied health professionals: a systematic review. *Clinical Goverance: An international journal*, 18(2): 139-154.

For peer review only

Wheeler S & Richards K (2007) *The impact of clinical supervision on counsellors and therapists, their practice and their clients: a systematic review of the literature* (BACP). British Association for Counselling and Psychotherapy: Lutterworth.

Resilience Training

Mealer M, Conrad D, Evans J, Jooste K, Solyntjes J, Rothbaum B, Moss M (2014) Feasibility and Acceptability of a Resilience Training Program for Intensive Care Unit Nurses, *Am J Crit Care*, 23(6): e97-105.

Peng L, Min L, Zuo X, Miao Y, Chen L, Yu Y, Liu B, Wang T (2014) Application of the Pennsylvania resilience training program on medical students. *Personality and Individual Differences*, 61-62: 47-51.

Sharma V, Bauer B, Prasad K, Sood A, Schroeder D (2012) Self help intervention to decrease stress and increase mindfulness: A pilot trial. *BMC Complement Altern Med*, 12(suppl 1): P253.

Sharma V, Sood A, Prasad K, Loehrer L, Schroeder D, Brent B (2014) Bibliotherapy to decrease stress and anxiety and increase resilience and mindfulness: A pilot trial. *Explore* (NY), 10(4): 248-52 *(reports on the same study as the 2012 paper).*

Sood A, Prasad K, Schroeder D, Prathibha V (2011) Stress management and resilience training among department of medicine faculty: A pilot randomized clinical trial. *J Gen Intern Med*, 26(8): 858-61.

Varker T & Devilly GJ (2012) An analogue trial of inoculation/resilience training for emergency services personnel: Proof of concept. *J Anxiety Disord*, 26(6): 696-701.

Critical Incidence Stress Debriefing

Matthews LR (1998) Effect of Staff Debriefing on Posttraumatic Stress Symptoms After Assaults by Community Housing Residents. *Psychiatric Services*, 49(2): 207-212.

O'Connor J & Jeavons S (2003) Perceived effectiveness of critical incident stress debriefing by Australian nurses. *Australian Journal of Advanced Nursing*, 20(4): 22-29.

Peer Supported Story Telling

Macpherson CF (2008) Peer-supported storytelling for grieving pediatric oncology nurses *J Pediatr Oncol Nurs* 25(3): 148-163.

Reflective Practice Groups

Abbas K & Pierce CM (2013) Time to reflect: Reflective practice groups for consultants and registrars on a neonatal and paediatric intensive care unit (N/PICU). *24th Annual Meeting of the European Society of Paediatric and Neonatal Intensive Care, ESPNIC 2013 Rotterdam Netherlands,* 39, S51-S52.

Binks C, Jones FW & Knight K (2013) Facilitating reflective practice groups in clinical psychology training: A phenomenological study. *Reflective Practice,* 14: 305-318.

Boyd EM & Fayles AW (1983) Reflective learning: Key to learning from experience. *Journal of Humanistic Psychology*, 23: 99–117.

Dawber C (2013) Reflective practice groups for nurses: A consultation liaison psychiatry nursing initiative: Part 2 - the evaluation. *International Journal of Mental Health Nursing,* 22: 241-248.

For peer review only

Gould B & Masters H (2004). Learning to make sense: the use of critical incident analysis in facilitated reflective groups of mental health student nurses. *Learning in Health & Social Care,* 3: 53-3.

Hansom J & Butler M (2003) Sharing reflections in midwifery practice. *British Journal of Midwifery,* 11: 34-37.

Hayes J (1998) Learning from practice: developing the reflective skills of forensic psychiatric nurses. *Psychiatric Care,* 5: 30-33.

Heneghan C, Wright J, & Watson G (2014) Clinical psychologists' experiences of reflective staff groups in inpatient psychiatric settings: A mixed methods study. *Clinical Psychology & Psychotherapy,* 21:324-340.

Hong LP & Chew L (2008) Reflective practice from the perspectives of the bachelor of nursing students in International Medical University (IMU). *Singapore Nursing Journal,* 35:5.

Johnston J & Paley G (2013) Mirror mirror on the ward: Who is the unfairest of them all? Reflections on reflective practice groups in acute psychiatric settings. *Psychoanalytic Psychotherapy,* 27:170-186.

Kiff J, Holmes G, & Cushway D (2010) Personal awareness / development groups and clinical training. *Clinical Psychology Forum*, 207: 30–34.

Knight K, Sperlinger D, & Maltby M (2010) Exploring the personal and professional impact of reflective practice groups: A survey of 18 cohorts from a UK clinical psychology training course. *Clinical Psychology & Psychotherapy,* 17: 427-437.

Kung JW, Eisenberg RL, & Slanetz PJ (2012) Reflective Practice as a Tool to Teach Digital Professionalism. *Academic Radiology,* 19: 1408-1414.

Manning A, Cronin P, Monaghan A & Rawlings-Anderson K (2009) Supporting students in practice: an exploration of reflective groups as a means of support. *Nurse Education in Practice,* 9: 176-183.

Parish C, Bradley L, & Franks V (1997) Managing the stress of caring in ITU: a reflective practice group. *British journal of nursing (Mark Allen Publishing),* 6: 1192-1196.

Platzer H, Blake D, & Ashford D (2000) An evaluation of process and outcomes from learning through reflective practice groups on a post-registration nursing course. *Journal of advanced nursing,* 31: 689-695.

Schon D (1983) *The Reflective Practitioner: How Professionals Think in Action*. New York, NY: Basic Books.

Psychosocial Intervention Training

Redhead K; Bradshaw T; Braynion P; Doyle M (2011) An evaluation of the outcomes of psychosocial intervention training for qualified and unqualified nursing staff working in a low-secure mental health unit. *Journal of Psychiatric & Mental Health Nursing*, 18(1): 59-66.

Ewers P; Bradshaw T; McGovern J; Ewers B (2002) Does training in psychosocial interventions reduce burnout rates in forensic nurses? *Journal of Advanced Nursing* 37(5): 470-476.

Doyle M, Kelly D, Clarke S, Braynion P(2007) Burnout: the impact of psychosocial interventions training. *Mental Health Practice* 10: 16–19.

For peer review only

Mindfulness Based Stress Reduction

Bazarko D, Cate RA, Azocar F, Kreitzer MJ (2013) The impact of an innovative mindfulness-based stress reduction program on the health and well-being of nurses employed in a corporate setting. *Journal of Workplace Behavioral Health*, 28(2): 107-133.

Beddoe AE. Murphy SO (2004) Does mindfulness decrease stress and foster empathy among nursing students? *J.Nurs.Educ*.43(7): 305-312.

Brady S, O'Connor N, Burgermeister D, Hanson P (2012) The Impact of Mindfulness Meditation in Promoting a Culture of Safety on an Acute Psychiatric Unit. *Perspect.Psychiatr.Care*, 48(3): 129-137.

Cohen-Katz J, Wiley S, Capuano T, Baker DM, Deitrick L, Shapiro S (2005) The effects of mindfulness-based stress reduction on nurse stress and burnout: a qualitative and quantitative study, part III. *Holist.Nurs.Pract*, 19(2): 78-86.

de Vibe M, Solhaug I, Tyssen R, Friborg O, Rosenvinge JH, Sorlie T, Bjorndal A (2013) Mindfulness training for stress management: a randomised controlled study of medical and psychology students. *BMC medical education*, 13: 107.

Foureur M, Besley K, Burton G, Yu N, Crisp J (2013) Enhancing the resilience of nurses and midwives: Pilot of a mindfulness-based program for increased health, sense of coherence and decreased depression, anxiety and stress. *Contemporary Nurse*, 45(1): 114-125.

Geary C, Rosenthal S, (2011) Sustained impact of MBSR on stress, well-being, and daily spiritual experiences for 1 year in academic health care employees. *The Journal of Alternative and Complementary Medicine*, 17(1): 939-944.

Goodman MJ, Schorling JB (2012) A mindfulness course decreases burnout and improves well-being among healthcare providers. *Int.J.Psychiatry Med*., 43(2): 119-128.

Hallman I, O'Connor, N, Hasenau, S, Brady S (2014) Improving the Culture of Safety on a High-Acuity Inpatient Child/Adolescent Psychiatric Unit by Mindfulness-Based Stress Reduction Training of Staff. *J.Child Adolesc.Psychiatr.Nurs*. 27(4): 183-189.

Irving JA, ParkSaltzman J, Fitzpatrick M, Dobkin PL, Chen A, Hutchinson T (2014) Experiences of health care professionals enrolled in mindfulness-based medical practice: A grounded theory model. *Mindfulness*, 5(1): 60-71.

Mackenzie CS, Poulin PA, Seidman-Carlson R (2006) A brief mindfulness-based stress reduction intervention for nurses and nurse aides. *Applied Nursing Research*, 19(2): 105-109.

Martin-Asuero A, Garcia-Banda G (2010) The mindfulness-based stress reduction program (MBSR) reduces stress-related psychological distress in healthcare professionals. *Spanish Journal of Psychology*, 13(2): 897-905.

Marx R, Strauss C, Williamson C, Karunavira T (2014) The eye of the storm: A feasibility study of an adapted mindfulness-based cognitive therapy (MBCT) group intervention to manage NHS staff stress. *The Cognitive Behaviour Therapist*, 7 Dec, Art e18-17.

Moody K; Kramer D, Santizo RO, Magro L, Wyshogrod D, Ambrosio J, Castillo C, Lieberman R, Stein J (2013) Helping the helpers: Mindfulness training for burnout in pediatric oncology-a pilot program. *Journal of Pediatric Oncology Nursing,* 30(5): 275-284.

Poulin PA, Mackenzie CS, Soloway G, Karayolas E (2008) Mindfulness training as an evidenced-based approach to reducing stress and promoting well-being among human services professionals. *International Journal of Health Promotion and Education*, 46(2): 72-80.

Rosenzweig S, Reibel DK, Greeson JM, Brainard GC, Hojat M (2003) Mindfulness-based stress reduction lowers psychological distress in medical students. *Teach.Learn.Med*., 15(2): 88-92.

Shapiro SL, Astin JA, Bishop SR, Cordova M (2005) Mindfulness-Based Stress Reduction for Health Care Professionals: Results From a Randomized Trial. *International Journal of Stress Management*, 12(2): 164-176.

Shapiro SL, Schwartz GE, Bonner G (1998) Effects of mindfulness-based stress reduction on medical and premedical students. *J.Behav.Med.*,21(6): 581-599.

For peer review only

PRISMA 2009 Checklist

Section/topic	#	Checklist item	Reported on page #
**TITLE**			
Title	1	Identify the report as a systematic review, meta-analysis, or both.	1
**ABSTRACT**			
Structured summary	2	Provide a structured summary including, as applicable: background; objectives; data sources; study eligibility criteria, participants, and interventions; study appraisal and synthesis methods; results; limitations; conclusions and implications of key findings; systematic review registration number.	2-3
**INTRODUCTION**			
Rationale	3	Describe the rationale for the review in the context of what is already known.	4-6
Objectives	4	Provide an explicit statement of questions being addressed with reference to participants, interventions, comparisons, outcomes, and study design (PICOS).	6
**METHODS**			
Protocol and registration	5	Indicate if a review protocol exists, if and where it can be accessed (e.g., Web address), and, if available, provide registration information including registration number.	n/a
Eligibility criteria	6	Specify study characteristics (e.g., PICOS, length of follow-up) and report characteristics (e.g., years considered, language, publication status) used as criteria for eligibility, giving rationale.	6-7
Information sources	7	Describe all information sources (e.g., databases with dates of coverage, contact with study authors to identify additional studies) in the search and date last searched.	6
Search	8	Present full electronic search strategy for at least one database, including any limits used, such that it could be repeated.	6
Study selection	9	State the process for selecting studies (i.e., screening, eligibility, included in systematic review, and, if applicable, included in the meta-analysis).	7
Data collection process	10	Describe method of data extraction from reports (e.g., piloted forms, independently, in duplicate) and any processes for obtaining and confirming data from investigators.	7
Data items	11	List and define all variables for which data were sought (e.g., PICOS, funding sources) and any assumptions and simplifications made.	n/a
Risk of bias in individual studies	12	Describe methods used for assessing risk of bias of individual studies (including specification of whether this was done at the study or outcome level), and how this information is to be used in any data synthesis.	7
Summary measures	13	State the principal summary measures (e.g., risk ratio, difference in means).	8
Synthesis of results	14	Describe the methods of handling data and combining results of studies, if done, including measures of consistency (e.g., I^2) for each meta-analysis.	n/a

For peer review only - http://bmjopen.bmj.com/site/about/guidelines.xhtml

PRISMA 2009 Checklist

Section/topic	#	Checklist item	Reported on page #
Risk of bias across studies	15	Specify any assessment of risk of bias that may affect the cumulative evidence (e.g., publication bias, selective reporting within studies).	n/a
Additional analyses	16	Describe methods of additional analyses (e.g., sensitivity or subgroup analyses, meta-regression), if done, indicating which were pre-specified.	n/a
**RESULTS**			
Study selection	17	Give numbers of studies screened, assessed for eligibility, and included in the review, with reasons for exclusions at each stage, ideally with a flow diagram.	11, 25 (Table 1)
Study characteristics	18	For each study, present characteristics for which data were extracted (e.g., study size, PICOS, follow-up period) and provide the citations.	28-32 (Table 2)
Risk of bias within studies	19	Present data on risk of bias of each study and, if available, any outcome level assessment (see item 12).	28-32 (Table 2)
Results of individual studies	20	For all outcomes considered (benefits or harms), present, for each study: (a) simple summary data for each intervention group (b) effect estimates and confidence intervals, ideally with a forest plot.	28-32 (Table 2)
Synthesis of results	21	Present results of each meta-analysis done, including confidence intervals and measures of consistency.	Narrative synthesis 11-12
Risk of bias across studies	22	Present results of any assessment of risk of bias across studies (see Item 15).	11-12
Additional analysis	23	Give results of additional analyses, if done (e.g., sensitivity or subgroup analyses, meta-regression [see Item 16]).	n/a
**DISCUSSION**			
Summary of evidence	24	Summarize the main findings including the strength of evidence for each main outcome; consider their relevance to key groups (e.g., healthcare providers, users, and policy makers).	15-16
Limitations	25	Discuss limitations at study and outcome level (e.g., risk of bias), and at review-level (e.g., incomplete retrieval of identified research, reporting bias).	18
Conclusions	26	Provide a general interpretation of the results in the context of other evidence, and implications for future research.	18
**FUNDING**			
Funding	27	Describe sources of funding for the systematic review and other support (e.g., supply of data); role of funders for the systematic review.	19

From: Moher D, Liberati A, Tetzlaff J, Altman DG, The PRISMA Group (2009). Preferred Reporting Items for Systematic Reviews and Meta-Analyses: The PRISMA Statement. PLoS Med 6(7): e1000097.

For peer review only - http://bmjopen.bmj.com/site/about/guidelines.xhtml

PRISMA 2009 Checklist

doi:10.1371/journal.pmed1000097 For more information, visit: www.prisma-statement.org. Page 2 of 2

For peer review only

BMJ Open

Can Schwartz Center Rounds support healthcare staff with emotional challenges at work, and how do they compare to other interventions aimed at providing similar support? A systematic review and scoping reviews

Journal:	*BMJ Open*
Manuscript ID	bmjopen-2018-024254.R1
Article Type:	Research
Date Submitted by the Author:	27-Jul-2018
Complete List of Authors:	Taylor, Cath; University of Surrey, School of Health Sciences<br>Xyrichis, Andreas; King's College London, Florence Nightingale Faculty of Nursing, Midwifery and Palliative Care<br>Leamy, Mary; King's College London, Florence Nightingale Faculty of Nursing, Midwifery and Palliative Care<br>Reynolds, Ellie; King's College London, Florence Nightingale Faculty of Nursing, Midwifery and Palliative Care<br>Maben, Jill; University of Surrey, School of Health Sciences
**Primary Subject Heading**:	Health services research
Secondary Subject Heading:	Occupational and environmental medicine
Keywords:	Schwartz Rounds, Occupational Stress, Staff Wellbeing, Systematic Review, Compassion, Reflection

For peer review only

SCHOLARONE™ Manuscripts

Can Schwartz Center Rounds support healthcare staff with emotional challenges at work, and how do they compare to other interventions aimed at providing similar support? A systematic review and scoping reviews

Authors:

*Cath Taylor, School of Health Sciences, University of Surrey, Guildford GU2 7XH, UK

Email: cath.taylor@surrey.ac.uk Tel: +44 1483 688862

Andreas Xyrichis, Florence Nightingale Faculty of Nursing, Midwifery and Palliative Care, Kings College London, London UK

Mary Leamy, Florence Nightingale Faculty of Nursing, Midwifery and Palliative Care, Kings College London, London UK

Ellie Reynolds, Florence Nightingale Faculty of Nursing, Midwifery and Palliative Care, Kings College London, London UK

Jill Maben, School of Health Sciences, University of Surrey, Guildford, UK

*corresponding author

Word count (excluding title page, abstract, references, figures and tables): 4024

For peer review only - http://bmjopen.bmj.com/site/about/guidelines.xhtml

Abstract

Objectives: (i) To synthesise the evidence-base for Schwartz Center Rounds® (Rounds) to assess any impact on healthcare staff and identify key features; (ii) to scope evidence for interventions with similar aims, and compare effectiveness and key features to Rounds.

Design: Systematic review of Rounds literature; scoping reviews of comparator interventions (Action Learning Sets; After Action Reviews; Balint Groups; Caregiver Support Programme; Clinical Supervision; Critical Incident Stress Debriefing; Mindfulness-Based Stress Reduction; Peer Supported Storytelling; Psychosocial Intervention Training; Reflective Practice Groups; Resilience Training).

Data Sources: PsychINFO, CINAHL, MEDLINE, and EMBASE, internet search engines; consultation with experts.

Eligibility criteria: Empirical evaluations (qualitative or quantitative); any healthcare staff in any healthcare setting; published in English.

Results: The overall evidence base for Rounds is limited. We developed a composite definition to aid comparison with other interventions from 41 documents containing a definition of Rounds. Twelve (ten studies) were empirical evaluations. All were of low/moderate quality (weak study designs including lack of control groups). Findings showed the value of Rounds to attenders, with a self-reported positive impact on individuals, their relationships with colleagues and patients, and wider cultural changes. The evidence for the comparative interventions was scant and also low/moderate quality. Some features of Rounds were shared by other interventions, but Rounds offer unique features including being open to all staff and having no expectation for verbal contribution by attenders.

For peer review only - http://bmjopen.bmj.com/site/about/guidelines.xhtml

Conclusions: Evidence of effectiveness for all interventions considered here remains limited. Methods that enable identification of core features related to effectiveness are needed to optimise benefit for individual staff members and organisations as a whole. A systems approach conceptualising workplace wellbeing ~~as~~ arising from both individual and environmental/structural factors, and comprising interventions both for assessing and improving the wellbeing of healthcare staff, is required. Schwartz Rounds could be considered as one strategy to enhance staff wellbeing.

Keywords: Schwartz Rounds, Occupational Stress, Staff wellbeing, Systematic Review, Compassion, Reflection

Article Summary: Strengths and limitations of this study

- This is the first systematic review of Schwartz Center Rounds®, a healthcare staff intervention from the US that has spread rapidly through UK healthcare organisations
- Additional scoping reviews of 11 interventions with similar aims to support the wellbeing of healthcare staff, enables a novel comparative analysis to key features of Schwartz Rounds.
- This paper compares other staff wellbeing interventions to Rounds, thereby resulting in a focus on key features of Rounds; we did not explicitly draw out key features of other interventions or compare them against each other.
- The use of scoping reviews for comparator interventions, and exclusion of evidence in populations other than healthcare staff means that some evidence may have been omitted.
- The heterogeneity of study designs and outcomes, and weak study designs, means that findings are summarized narratively rather than using meta-analysis.

For peer review only

Introduction

In this paper, we report the systematic review of evidence regarding Schwartz Center Rounds® (Rounds) and conduct a comparative analysis to eleven interventions also broadly aimed at supporting healthcare staff with the emotional challenges of their work. In doing so, we define Rounds from the literature and discuss the future potential use of interventions to support staff with the emotional challenges of providing healthcare. Healthcare providers are amongst the largest employers in many countries worldwide. For example, the UK NHS employs 1.5 million staff,[1] and in 2014 there were approximately 1.8 million physicians,[2] and 3.4 million nurses[3] across the EU. Provision of healthcare relies upon both clinical and non-clinical staff (e.g. managers, administrators, porters/orderlies, caterers, and domestic staff) all of whom may be impacted by the emotional challenges they face in their interactions with the patients and families they come across in day to day life.

Numerous publications have highlighted the high prevalence of psychological morbidity amongst healthcare staff in both clinical and non-clinical roles, and in many different countries worldwide.[4-10] Indeed, studies have typically reported between a quarter to a third of healthcare staff to have levels of psychological distress indicative of the need for clinical intervention, and in the UK mental health reasons explain a third of all NHS sickness absence, costing approximately £1bn (of the total £2.4bn cost of sickness absence in 2015).[11] Together with the clear consequences of this for their wellbeing and quality of life, and impact on their families, there is now increasing recognition of the link between the wellbeing of healthcare staff and quality of patient care (in relation to both patient experience, and clinical outcomes).[12-15]

For peer review only - http://bmjopen.bmj.com/site/about/guidelines.xhtml

Consequently, the wellbeing of healthcare staff is high on the agenda of healthcare organisations in the UK and worldwide.[16-21] In the UK, NICE guidance published in 2009 recommended that organisations take a strategic approach to tackling staff wellbeing, encompassing approaches that focus on both prevention and treatment, and that include interventions for individuals as well as "organisation-wide approaches that encompass all employees".[22] However, the reviews underpinning this guidance highlighted the poor quality of evidence overall and in particular the limited evidence on organisation-wide policies or approaches, with the strongest evidence in relation to interventions aimed at stress management for individuals.[22]

Schwartz Rounds are a rare example of an organisation-wide intervention that has seen rapid spread across healthcare organisations in the UK.[23] Rounds originated in the U.S. where they now run in over 430 organisations. After a pilot introduction to two UK hospitals in 2009, they now run in over 170 UK health and social care organisations (hospitals, hospices, community settings). They were developed to support healthcare staff to deliver compassionate care by providing a safe space where staff could openly share and reflect on the emotional, social and ethical challenges faced at work. The premise is that caregivers will be more able to make personal connections with colleagues and patients if they have insight into their own responses and feelings. Their rapid adoption in the UK was despite a limited evidence base, though attendance at Rounds was reported to be associated with improved compassion for patients, better teamwork, and reduced stress in staff members, as well as having a positive impact on organisational culture.[24-25] Consequently, the National Institute for Health Research commissioned a national evaluation of Rounds that has recently concluded,[26] supporting these earlier findings and showing attendance at Rounds to be associated with a reduction in psychiatric morbidity. A key component of the evaluation,

For peer review only - http://bmjopen.bmj.com/site/about/guidelines.xhtml

intended to support organisational decision-making regarding staff wellbeing interventions, was to review the evidence for Schwartz Rounds and contextualise them by comparing the features of Rounds to other staff wellbeing interventions with similar aims. This paper reports the results from this, and thereby aims to answer the following review questions:

1) What are the defining features of Schwartz Center Rounds®, and what is their evidence base?
2) What comparable interventions providing staff support/reflective space exist, what key features do they share with Schwartz Rounds and what is their evidence base in healthcare professionals?

Specifically, we aim to (i) identify key features of Rounds by synthesising published descriptions of Rounds to create a composite definition; (ii) systematically review and appraise all empirical evaluations of Rounds; (iii) identify comparative interventions, describe their key features and scope their evidence base; and (iv) document similarities and differences between Rounds and comparative interventions.

Methods

The review of Schwartz Rounds literature followed PRISMA systematic literature review guidance where applicable.

Search strategy

The search strategies for the systematic review of Rounds literature involved: (i) a traditional database search (PsychINFO, CINAHL, MEDLINE, and EMBASE to give comprehensive coverage of medical, psychological, nursing and social sciences literature). As an example, the MEDLINE database search for Schwartz Rounds was: (Schwartz adj2 Round*).mp. [mp=title, abstract, original title, name of substance word, subject heading word, keyword heading word, protocol supplementary concept word, rare disease supplementary concept word, unique identifier] (ii) use of internet search engines; and (iii) consultation with

For peer review only - http://bmjopen.bmj.com/site/about/guidelines.xhtml

experts. Inclusion criteria included having a health professional sample (either qualified or trainee) and empirically evaluate the intervention using qualitative and/or quantitative methods. The review excluded non-English language sources, unpublished dissertations/theses and any papers not accessible via the institution's online library, Google Scholar or directly from the journal website. All records were pooled together into a bibliographic database. First, records were screened to exclude duplicate entries. Second, the title and abstract of remaining records was reviewed for eligibility. All database searches were conducted between 14th October 2014 and 5th February 2015, though searches for Schwartz Rounds evaluations and consultation with experts continued until September 2017.

Data extraction and quality appraisal

Standard data items were extracted to describe included papers (e.g. citation, country, setting, population/sample, overall design etc) and the evaluation (e.g. length of evaluation; data collection method/s; outcome measures; key findings) using extraction sheets that were developed and piloted by all data extractors. In addition, items were developed that were specific to each intervention, for example, whether group or individual focused, size of group, length/number of sessions, content of sessions, whether facilitated or not (and if facilitated whether training/supervision was provided). Quality assessment of qualitative and quantitative primary studies was undertaken for each study using the tools developed by Jones et al[27] which include assessment of key criteria and then an overall rating (High - No or few flaws; Moderate - Some flaws; Low - Significant flaws). Mixed methods studies were, in addition, assessed against the six criteria for good reporting of mixed methods studies developed by O'Cathain et al[28]. Quality was rated low (<3 criteria were met); moderate (3-4) or high (5+).

For peer review only - http://bmjopen.bmj.com/site/about/guidelines.xhtml

Synthesis

Thematic analysis of the types of outcomes reported resulted in the identification of three categories relating to: a) self; b) others (e.g. patients, colleagues); or c) wider organisation (e.g. changes to policies; organisational metrics such as safety or satisfaction). Findings are presented according to these three categories. Finally the overall quality of the evidence base for each intervention is described based on the range in quality for individual studies.

Constructing a composite definition of Schwartz Rounds

Whilst the Schwartz Center for Compassionate Care, where Rounds originated, have a description of Schwartz Rounds on their website, this was found to omit key aspects of their design that we knew from scoping the literature to be important (e.g. that it is an ongoing programme, time-fixed in length, that food is provided, that it is open to all staff, and that panellists stories are pre-prepared. Therefore, we constructed a 'composite' definition based on descriptions used in Rounds literature in order to determine the key features of Rounds for comparison with other interventions. For this process, we included all literature (including non-empirical literature for example letters, editorials) providing it included a description of Rounds. Text describing Rounds (what they were and their intended aims, e.g. structure and purpose, as well as any text describing what they were 'not') was extracted from published accounts. The text was analysed thematically by four team members independently (CT, JM, ML, MH), core concepts were discussed and agreed, and a single definition was produced. The face validity of the definition was confirmed after review by study advisory and steering group members.

Scoping reviews of comparative interventions

For peer review only - http://bmjopen.bmj.com/site/about/guidelines.xhtml

The reviews of comparable interventions followed an interpretative scoping literature review methodology based on the framework outlined by Arksey and O'Malley[29]. The searching, data extraction and synthesis followed similar steps to the review of Schwartz Rounds literature (except where noted below) but instead of producing a detailed critique and review of individual studies they were instead aimed at producing a summary description of the evidence base in relation to size, scope and quality, and used to extract data relevant for the comparative analysis. For each intervention, the number/type of included papers was recorded, and each intervention was described in relation to its original format (e.g. number of participants, original setting and healthcare setting/s, and intended aims/outcomes); and the variability in its application within the literature (fidelity to original format). Main findings were examined across all interventions and analysed thematically (using the same categories as for Schwartz Rounds: self, others, organisation) to enable synthesis within, and comparison across, each intervention.

Identification of comparative interventions to include

We aimed to identify interventions that support health professionals with the emotional challenges of delivering patient care. Initially we identified aspects that were fundamental to Rounds, including providing an opportunity for reflection, disclosure, and offering psychological safety; and these informed choices regarding potential comparative interventions. Included interventions needed to focus on psychological (as opposed to physical) wellbeing of staff; be person-directed (versus work directed); and provide primarily emotional rather than cognitive/clinical support (thus for example excluding mortality/morbidity meetings which aim to provide lessons in terms of cognitive errors or systems issues). Although Rounds are a 'group' (rather than individual) intervention we chose not to limit comparative interventions by this characteristic, due to the importance of

For peer review only - http://bmjopen.bmj.com/site/about/guidelines.xhtml

reflection and/or disclosure as a key potential mechanism in Rounds that is shared by other interventions that are not group-based. Potential comparative interventions were identified through published reviews of psychological/emotional support interventions for healthcare staff[30-33] and through consultation with steering and advisory group members (with expertise in Rounds/wellbeing interventions in healthcare). A total of 11 interventions were scoped: Action Learning Sets; After Action Reviews; Balint Groups; Caregiver Support Programme; Clinical Supervision; Critical Incident Stress Debriefing; Mindfulness-Based Stress Reduction; Peer Supported Storytelling; Psychosocial Intervention Training; Reflective Practice Groups; Resilience Training.

Comparative analysis to Schwartz Rounds

The composite definition of Rounds was disaggregated into its individual descriptive features which were extracted into a table, together with the features that were 'not' part of Rounds. Further clarification was added for some descriptive features to ensure clarity of meaning (e.g. "reflection" became "provides an explicit opportunity for reflection"). The description of each comparative intervention was then reviewed by the research team and assessed in relation to whether or not it also provided each of the key features of Rounds. The face validity of the comparison between Rounds and other interventions was confirmed with study advisory and steering groups (with expertise in Rounds/healthcare staff wellbeing interventions).

Patient Involvement

We actively involved patients through membership of the Project Steering Group (PSG) which included two Patient Public Involvement (PPI) representatives (Havi Carel, Christine Chapman) who had previously provided input to the original funding application. The PSG

For peer review only - http://bmjopen.bmj.com/site/about/guidelines.xhtml

provided oversight to all aspects of the study, and alongside other group members our PPI representatives and Rounds staff members advised on design, inclusion of comparative interventions, and commented on the findings.

Results

Key features of Rounds

Forty-three documents/sources were included in the review of descriptions of Rounds (Table 1) which allowed development of the definition.[24,25,34-74]. The majority (n=33) were non-empirical publications (e.g. commentaries, descriptive reports of a single Round). The thematic synthesis resulted in the production of the composite definition (supplementary file 1), a summary version is provided in Table 1.

Evidence base for Rounds: results from the systematic review

Twelve empirical evaluations of Rounds were included (Table 2) arising from ten studies (four in the USA, six in the UK). Most were mixed methods evaluations, typically comprising attenders completing evaluation forms post-Round attendance, followed by interviews or focus groups (n=5), one mixed method study comprised case studies (observation/interviews) together with descriptive analysis of evaluation forms[75] and one that used both quantitative and qualitative methods to analyse evaluation forms.[76] Two

For peer review only - http://bmjopen.bmj.com/site/about/guidelines.xhtml

were quantitative studies, and one qualitative study. Only one study included non-attenders[66] (Table 2).

Overall quality of the evidence-base was assessed to be low/moderate. Most studies had study designs prone to risk of bias (e.g. cross-sectional), used non-validated questionnaires (typically self-report views/satisfaction with Rounds and impact of attendance), and none of the quantitative evaluations had control group (non-attender) comparisons. Little information was provided on the samples/sampling frames in quantitative studies (e.g. in relation to breadth of professional group representation or role in Rounds), nor were findings analysed or presented in relation to such factors. In two studies that did report the characteristics of their quantitative sample, most were female and of white ethnicity, and nurses predominated (but neither study reported the seniority of nurse).[24,47] Findings from these studies included that Rounds are highly valued by attenders (though represented a small proportion of total staff). Most studies reported positive impact on 'self' (e.g. improved wellbeing, coping)[24-25,44,47,49,51,59,66,70,75-76] and impact on patients (increased compassion, empathy),[24-25,51,59,66,70,75-76] and colleagues (improved teamwork, compassion/empathy).[24-25,44,47,50-51,59,66,75-76] Six studies provide evidence of wider institutional impacts from interviews with attenders[24-25,44,51,66,75-76] (Table 2). Three of the included studies were evaluations of Rounds adapted for educational purposes;[39,50,70] all reporting that Rounds were felt to be useful and that students gained knowledge/understanding about the emotional side of providing patient care.

Comparative interventions: results from the scoping reviews

Electronic searches for the 11 comparative interventions yielded a total of 1725 papers, of which 146 were included (ranging between one and 64 across interventions, Table 1, see

For peer review only - http://bmjopen.bmj.com/site/about/guidelines.xhtml

supplementary file 2 for included references). A number of publications (n=253) were not obtainable due to being published in sources that no longer existed or not available through institutional subscription and internet searches. The largest evidence base was for clinical supervision (n=64) followed by Balint groups (n=26). Half of the studies were quantitative (n=74: RCT, observational, quasi experimental), 41 were qualitative (mixed designs, interviews, focus groups), 22 were mixed-methods, and nine were secondary studies (literature reviews). The literature was international with the majority of studies from the USA and the UK; other countries represented included Canada, Australia, Finland, Norway, Sweden, Croatia, Spain, Italy, Israel and South Africa. There was a distinct lack of studies from Asia, although that may be a reflection of the English language limit.

For most interventions, high-quality evidence was sparse. Populations for many of the interventions lacked diversity across health professions and settings, with many mostly nursing-focused. The aims of studies varied widely, with a few aimed at assessing efficacy or effectiveness but most were small-scale exploratory descriptive studies. The content and format of interventions (fidelity) was in most cases widely heterogeneous (and/or lacked detail), and consequently synthesis of findings is problematic. Most of the quantitative evaluations across all interventions relied upon weaker study designs (e.g. cross-sectional studies, post-intervention evaluations, lacking control comparisons), used non-probability sampling, had small samples likely to be underpowered, and used non-validated outcomes measures. Many qualitative studies also lacked clear reporting of aspects of rigour (e.g. limited reporting of member checking, deviant cases, reflexivity or evidence of data saturation). A summary of the evidence base for each intervention is provided in Supplementary file 3.

For peer review only - http://bmjopen.bmj.com/site/about/guidelines.xhtml

Synthesis

Most interventions presented evidence in relation to all three categories of outcomes ("self", "others" and "organisation"), though evidence for Resilience training, Mindfulness-based stress reduction and Reflective practice groups lacked inclusion of organisational outcomes. All of the interventions had evidence of positive benefits to self (e.g. raised self-awareness, resilience, job satisfaction, empowerment, or overall wellbeing), and most provided some evidence of positive benefits to 'others'. Impact on patients included fostering of better provider-service user relationships, communication with and/or attitudes towards patients; and improved patient-centredness, knowledge of patients' suffering and empathy. Impact on colleagues, included associations with better teamwork, peer support and knowledge/understanding of colleagues.

At organisational-level, there was evidence from some interventions of association with improved practice, for example, reductions in unnecessary prescriptions, increased uptake of psychosocial support (Balint groups), reduction in task and coordination errors and increased uptake of post-fall huddles (After action reviews). Two interventions provided evidence of a positive impact on the workforce, including providing opportunities for mentoring and advice (Action learning sets) and improved staff retention (Clinical supervision).

Schwartz vs alternative interventions: comparative features

In comparison to the other interventions, Rounds offer a unique organisation-wide 'all-staff' forum to share stories about the emotional impact of providing patient care (Table 3). Whilst many of the other interventions expect 'open, honest communication' as a key feature, and provide an explicit opportunity for reflection, none are open to all staff (e.g. clinical and non-clinical, voluntary attendance), and many are not ongoing programmes but instead are one-off training courses or events. Some of the training interventions (e.g. mindfulness-based stress

For peer review only - http://bmjopen.bmj.com/site/about/guidelines.xhtml

reduction, or resilience training) are multidisciplinary in training attendance, but conduct/practice of the intervention occurs subsequently and is individual, compared to Schwartz (and other interventions such as Balint Groups) where learning and practice take place simultaneously in group settings.

Other key aspects in which Rounds are distinct from the comparative interventions relate to what Rounds are intentionally 'not' meant to be. In particular, discussions within Rounds should not 'problem solve' in order to avoid focus on the clinical decision-making in a patient case, whereas problem solving/action planning are key features of many of the other interventions (e.g. Action learning sets, After action reviews, Critical incident stress debriefing). Most of the comparative interventions also offered flexibility in format, compared to Rounds which require a contractual license (with stipulated conditions) obtained via the Schwartz Center for Compassionate Care (USA) or Point of Care Foundation (UK).

Arguably the closest types of interventions to Rounds are Balint groups (though rooted in uni-disciplinary primary care - physicians only- with closed membership), and Reflective practice groups (again generally closed membership and can be uni-disciplinary). In particular, both are ongoing group programmes in which challenging/rewarding experiences about delivering patient care are shared and discussions are facilitated, and both provide the opportunity to give and/or receive peer support in safe and confidential environments. However, neither offers an organisation-wide opportunity for staff to attend, and both would have an expectation that members/attenders would contribute, whereas in Rounds attenders can choose to be silent listeners. Clinical supervision can also provide an opportunity to reflect on the emotional and ethical challenges of care without problem-solving/action planning –but unlike Rounds this usually occurs in a one to one situation, not group, and requires those being supervised to verbally contribute.

For peer review only - http://bmjopen.bmj.com/site/about/guidelines.xhtml

Discussion

Our work revealed a rich portfolio of available interventions to support staff with the emotional challenges of providing healthcare, each designed with different audiences and uses in mind. The evidence base regarding the effectiveness of these in the main remains weak, and more should be done to examine these more systematically. The studies reviewed here show some evidence of impact at different levels, and future work should seek to unpick which interventions work best, under which conditions and for which participants. To our knowledge this is the first comparative review of staff wellbeing interventions.

Given the high rates of work-related stress and mental health issues amongst healthcare staff it is not acceptable for employers not to act, despite the weak evidence base for most approaches and interventions currently. Some staff groups have clinical supervision for example as an integral part of their work (mental health nurses; midwives; psychologists and social workers) whereas most doctors and nurses do not, and such staff often have little or no support with the emotional, social and ethical challenges of their work. Non-clinical staff – who may have much contact with patients and the events they encounter - are even more neglected in relation to the impact of delivering patient care on them. Selection of interventions should be based on a strategic approach that incorporates needs assessment, implementation of interventions/approaches and policies, and monitoring and review to determine the impact of these and refine/revise as necessary. There is a need for a range of approaches, not a one size fits all and our work does not suggest an either/ or approach for individual interventions. Rounds should not be seen as a replacement for or instead of clinical supervision (or other support/interventions) but could be offered to staff in addition. Organisation-wide interventions are important to tackle workplace environmental/cultural

For peer review only - http://bmjopen.bmj.com/site/about/guidelines.xhtml

factors that impact on wellbeing; to change attitudes and cultural norms around staff needing support as well as changing conversations in organisations around empathy, compassion and the support required to deliver these. Involving all employees may improve co-worker and supervisor support, which in turn can facilitate the development of a supportive workplace environment that reduces stress by improving attitudes and behaviours.[77] Compared to other interventions reviewed here, Rounds offer a unique organisation-wide "all staff" forum to reflect on the emotional impact of providing patient care, offering opportunities for staff to reflect, whether or not they choose to disclose/contribute to discussions, and accruing evidence suggests they may have many benefits to individuals, others (colleagues, patients) as well as wider organisational impacts.[26]

Schwartz Rounds were originally conceived to meet a very specific identified need in healthcare: to support healthcare providers to be compassionate to patients through giving them insight into their own thoughts, feelings and behaviours.[26] In the UK the reasons given for adoption has been more about staff wellbeing, in line with evidence linking quality of patient care and experience with staff wellbeing.[13, 23] Unlike many of the other interventions, they have a structured format, and are specifically not intended to be 'problem-solving'. In doing so they provide a 'counter-cultural' space that differs from the protocol-driven, outcome-orientated healthcare environment that values emotional stoicism: "Good Rounds shift an organisation and its workers away from their default position of urgent action, reaction and problem solving to an hour of stillness and slowness".[78,p41] A key ingredient supporting Rounds to meet their intended aims is good facilitation, thus the role of the facilitator is key. Unlike the facilitation role in other interventions we reviewed, where there was often much variability in relation 'fidelity', in the UK, it is mandatory for Rounds facilitators to attend training provided by the Point of Care Foundation (PoCF, the UK

For peer review only - http://bmjopen.bmj.com/site/about/guidelines.xhtml

Licence holder for Schwartz Rounds), and they receive ongoing support from Schwartz mentors. It is recommended that there are at least two facilitators in each organisation, and the PoCF state that it helps if facilitators have experience of group work, and managing difficult emotions (many have psychology or social work backgrounds). In our national evaluation, we found despite most having these skills and background, they often shouldered the responsibility for Rounds on their own (some having only one facilitator too), which we found to impact negatively on their wellbeing, and on the sustainability of Rounds, recommending that a focus on facilitator support, and succession planning would be beneficial for Rounds [26, 79]

Workforce interventions are often complex in nature, with many components and aims. Their evaluation is thereby challenging, particularly with regard to attributing any changes to outcomes to the intervention as opposed to other causes within the organisation/system. The challenge of conducting a robust evaluations of organisation-wide interventions may be one explanation as to why such evidence is so sparse,[22] and for why there is instead a predominance of evidence regarding individually targeted interventions such as mindfulness-based stress reduction. The application of new methodologies to address these challenges, such as realist evaluation, could enable a more robust understanding of how and why interventions work (or don't work), and has recently been applied in the first UK national evaluation of Schwartz Rounds.[26]

Limitations

The focus of this review on the evidence within healthcare staff meant that wider evidence for some interventions, beyond healthcare, was not considered. Also, the scoping methods applied to the comparable interventions inevitably means that some relevant evidence may

For peer review only - http://bmjopen.bmj.com/site/about/guidelines.xhtml

have been omitted, though systematic electronic searching and consultation with experts aimed to minimise this risk. The rapid uptake of Rounds in the UK and need to contextualise them within staff wellbeing interventions, informed the design of this review. It was thereby a review that compared other interventions to the key features of Rounds, and did not thereby compare the key features of all the other interventions to each other, apart from by describing and synthesising their origins and evidence base.

Conclusion

Given the time and resources already committed to the interventions considered here, it is important to determine how best to identify the core features of effectiveness to optimise benefit for individual staff members and organisations as a whole. This work has now been undertaken for Schwartz Rounds using a realist-informed methodology that has identified the contextual factors that influence how and for whom Schwartz Rounds work, resulting in an organisational guide giving practical guidance and recommendations for organisations to maximise the effectiveness of Rounds in their organisations.[26,79] The application of similar methodologies for other interventions such as clinical supervision and Balint groups may further help ensure optimal outcomes. A systems approach as opposed to an individual approach to tackling staff wellbeing, in order to improve patient care, is required, comprising effective interventions for assessing and improving the wellbeing of healthcare staff.

Funding:

This work was supported by the National Institute for Health Research Health Services and Delivery Research Programme (project number 13/07/49).

For peer review only - http://bmjopen.bmj.com/site/about/guidelines.xhtml

Disclaimer:

The views and opinions expressed therein are those of the authors and do not necessarily reflect those of the of the NHS, the NIHR, MRC, CCF, NETSCC, the HS&DR programme or the Department of Health.

Author contributions:

CT and JM conceived the original idea and designed the study; AX designed the search strategies and performed the database searches; CT, AX, ML and ER performed internet searches; screened titles and abstracts independently; extracted data and assessed quality for three interventions each; CT, JM, ML and ER created the composite definition of Rounds; All authors contributed to the overall synthesis and comparative analysis; CT drafted the manuscript; AX, ML, ER and JM revised critically the manuscript. All the authors approved the final version of the manuscript.

Acknowledgements:

We thank the following members of our project advisory group and steering groups for their guidance and comments throughout the study.

Advisory Group: Joanna Goodrich, Barbara Wren, Jenny Firth-Cozens, Michael West, John Pepper, Glenn Robert.

Steering Group: Dean Fathers, Sharon Fleming, Nicola Mackintosh, Rachel Massey-Chase, Katherine Hopkins, Jude Bayly, Havi Carel, Christine Chapman, Elisabeth Buggins, Jennifer Gardner, Ruth Harris, and Lisl Klein.

We also thank Michelle Hope (MH) for her contribution to the analysis of definitions of Schwartz Rounds.

Competing interests: Jill Maben reports that she was a member of an advisory group from 2006 to 2009, advising on the development of the Point of Care project at The King's Fund,

For peer review only - http://bmjopen.bmj.com/site/about/guidelines.xhtml

and a member of the Point of Care Foundation Board 2013–14; she stepped down as board member at the start of the evaluation. All other authors declare no conflicts of interest.

Data sharing statement: No additional data are available

For peer review only

For peer review only - http://bmjopen.bmj.com/site/about/guidelines.xhtml

References

1. NHS (2018) https://www.nhs.uk/NHSEngland/thenhs/about/Pages/overview.aspx. Last accessed 15/2/18
2. Europa Eurostat (2018) http://ec.europa.eu/eurostat/statistics-explained/index.php/Healthcare_personnel_statistics_-_physicians. Last accessed 15/2/18.
3. Europa Eurostat (2018) http://ec.europa.eu/eurostat/statistics-explained/index.php/Healthcare_personnel_statistics_-_nursing_and_caring_professionals Last accessed 15/2/18.
4. Taylor C., Graham J., Potts H., Candy J., Richards M. and Ramirez A. Changes in mental health of UK hospital consultants since the mid-1990s Lancet. 2005; 366:742-44
5. Wall T.D., Bolton R.I., Borrill C.S., Carter A.J., Golya D.A., Hardy G.E., et al. Minor psychiatric disorders in NHS Trust employees: occupational and gender differences. British Journal of Psychiatry. 1997; 171:519-23.
6. Maslach C., Jackson S.E. Burnout in health professionals: a social psychological analysis In: Sanders GS, Suls J, editors. Social Psychology of Health and Illness. Hillsdale NJ: Lawrence Erlbaum Associates; 1982.
7. Arigoni, F., Bovier, P.A., Mermillod, B., Waltz, P., Sappino, A.-P. Prevalence of burnout among Swiss cancer clinicians, paediatricians and general practitioners: who are most at risk?. *Support Care Cancer*. 2009;17:75–81.
8. Trufelli, D., Bensi, C., Garcia, J. Burnout in cancer professionals: a systematic review and meta-analysis. *Eur J Cancer Care*. 2008;6:31.
9. Bressi, C., Manenti, S., Porcellana, M. et al, Haemato-oncology and burnout: an Italian survey. *Br J Cancer*. 2008;98:1046–1052.
10. Harvey, S.B., Laird, B., Henderson, M., Hotopf, M. The mental health of health care professionals: a review for the Department of Health. Department of Health, London; 2009
11. Quality Watch (2018) http://www.qualitywatch.org.uk/indicator/nhs-staff-sickness-absence# Last accessed 15/2/18.
12. Taylor C., Graham J., Potts H., Candy J., Richards M., Ramirez A. Impact of hospital consultants' poor mental health on patient care. British Journal of Psychiatry. 2007; 190:268-269
13. Maben J., Peccei R., Adams M., Robert G., Richardson A., Murrells T. and Morrow E. Patients' experiences of care and the influence of staff motivation, affect and well-being. Final report NIHR Service Delivery and Organisation programme; 2012. http://www.netscc.ac.uk/hsdr/files/project/SDO_FR_08-1819-213_V01.pdf
14. Maben J., Adams M., Peccei R., Murrells T., Robert G. Poppets and parcels: the links between staff experience of work and acutely ill older people's experience of hospital care. International Journal of Older People's Nursing. 2012;7(2):83-94
15. Raleigh V., Hussey D., Seccombe I. Do associations between staff and inpatient feedback have the potential for improving patient experience? An analysis of surveys in NHS acute trusts in England. Quality and safety in health care; 2009. 18:347-54.
16. Australian Government (2018) Healthy workers healthy futures initiative. http://www.healthyworkers.gov.au/ Last accessed 15.2.18.
17. American Nurses Association (2018) http://www.healthynursehealthynation.org Last accessed 15.2.18
18. Royal College of Physicians (2015)Work and Wellbeing in the NHS: why staff health matters to patient care. https://www.rcpsych.ac.uk/pdf/RCP-%20WorkWellbeingNHS.pdf 12th October 2015. Last accessed 15.2.18.
19. Rao AS, Bhutani G, Dosanjh N, Clarke J, Hughes JH, Easton S, Van Laar D, Cohen-Tovee E (2016) Psychological Wellbeing and Resilience: Resetting the Balance http://www.bps.org.uk/system/files/Public%20files/ar_v10_resetting_the_balance_160516.docx.pdf 2016 Last accessed 15.2.18.

For peer review only - http://bmjopen.bmj.com/site/about/guidelines.xhtml

20. The Kings Fund (2015) Staff Engagement: six building blocks for harnessing the creativity and enthusiasm of NHS staff. https://www.kingsfund.org.uk/sites/default/files/field/field_publication_file/staff-engagement-feb-2015.pdf Last accessed 15.2.18.
21. NHS Wales (2017) Staff Psychological Wellbeing Policy http://www.wales.nhs.uk/sitesplus/documents/862/340-PsychologicalWellbeingPolicy-ext31.12.17.pdf Last accessed 15.2.18.
22. NICE (2009) Mental wellbeing at work. Public Health Guidance (PH22) https://www.nice.org.uk/guidance/ph22 Last accessed 15.2.18.
23. Robert G, Philippou J, Leamy M, Reynolds E, Ross S, Bennett L, Taylor C, Shuldham C, & Maben J (2017) Exploring the adoption of Schwartz Center Rounds as an organisational innovation to improve staff wellbeing in England, 2009-2015. *BMJ Open*, 7(1): 1-10.
24. Lown BA, Manning CF The Schwartz Center Rounds: Evaluation of an Interdisciplinary Approach to Enhancing Patient-Centered Communication, Teamwork, and Provider Support Acad Med. 2010; 85:1073–1081.
25. Goodrich J (2012) Supporting hospital staff to provide compassionate care: Do Schwartz Center Rounds work in English hospitals? *JRSM*, **105**: 117–122. DOI 10.1258/jrsm.2011.110183.
26. Maben J, Taylor C, Dawson, J, Leamy M, McCarthy I, Reynolds E, Ross S, Shuldham, C, Bennett L, Foot C (2018) A realist informed mixed methods evaluation of Schwartz Center Rounds® in England. First Look Summary available from: https://www.journalslibrary.nihr.ac.uk/programmes/hsdr/130749/#/ Last accessed 15.2.18.
27. Jones CEL, Maben J, Jack RH, et al. A systematic review of barriers to early presentation and diagnosis with breast cancer among black women. BMJ Open 2014; 4:e004076. doi:10.1136/bmjopen-2013-004076
28. O'Cathain A, Murphy E, Nicholl J (2008) The quality of mixed methods studies in health services research. J Health Serv Res Policy 13(2):92-8. doi: 10.1258/jhsrp.2007.007074
29. Arksey, H. and O'Malley, L. (2005) Scoping studies: towards a methodological framework. *International Journal of Social Research Methodology*, 8(1):19-32.
30. Ruotsalainen J, Serra C, Marine A, Verbeek J. Systematic review of interventions for reducing occupational stress in health care workers. *Scand J Work Environ Health* 2008;**34**:169-78.
31. Boorman S. *NHS Health and Wellbeing Review*. London: Department of Health; 2009.
32. Ruotsalainen JH, Verbeek JH, Mariné A, Serra C. Preventing occupational stress in healthcare workers. *Cochrane Database of Systematic Reviews* 2015.
33. Harvey SB, Laird B, Henderson M, Hotopf M. The mental health of health care professionals: A review for the Department of Health. In. London; 2009.
34. Anon (2008) A place to Heal (2008) https://www.nurse.com/blog/2008/09/08/a-place-to-heal/ (Last accessed 15/2/18).
35. Barnard P (2013) Schwartz Rounds: Librarian Roles and Opportunities. *Journal of Hospital Librarianship*, 13:1, 71-78, DOI: 10.1080/15323269.2013.743368.
36. Baverstock AC, Finlay FO (2015) Maintaining compassion and preventing compassion fatigue: a practical guide *Arch Dis Child Educ Pract Ed* **0**:1–5. doi:10.1136/archdischild-2015-308582.
37. Berry M, Chaggar R (2014) Introducing Schwartz Center Rounds® to Watford General Hospital – challenging the status quo. *Anaesthesia*, **69**(Suppl. 4): 46 (no.72) Abstract Only.
38. Booth M (2014) Schwartz Center Rounds – a moment to reflect at St Wilfrid's Hospice eHospice: available from: http://www.ehospice.com/uk/Default/tabid/10697/ArticleId/11798 (last accessed 15/2/18).
39. Corless IB, Michel TH, Nicholas M, Jameson D, Purtilo R, Angelique D (2009) Educating Health Professions Students About the Issues Involved in Communicating Effectively: A Novel Approach *Journal of Nursing Education*, **48**(7): 367-373.
40. Cornwell J, Goodrich J (2010) Supporting staff to deliver compassionate care using Schwartz Center rounds – a UK pilot. *Nursing Times* **106**(5):10-12.

For peer review only - http://bmjopen.bmj.com/site/about/guidelines.xhtml

41. Davis C (2011) Forum to help staff deal with day-to-day stress. *Nursing Management* **18**(4): 18–21.
42. Dean E (2013) Time for Reflection. *Nursing Standard* **27**(48): 61.
43. Dedier J, Penson R, Williams W, Lynch T (1999) Race, Ethnicity, and the Patient-Caregiver Relationship. *The Oncologist* **4:**325-331 [included to represent the many publications with the same definition from the Schwartz Center, Massachusetts General Hospital, Boston USA].
44. Deppoliti DI, Côté-Arsenault D, Myers G, Barry J, Randolph C, Tanner B (2015) Evaluating Schwartz Center Rounds® in an urban hospital center. *Journal of Health Organization and Management*, **29**(7): 973 – 987.
45. Foster S (2015) Schwartz Rounds: our experience. *British Journal of Nursing* **24**(5):309.
46. Gannon C (2014) Contrasting Schwartz Rounds with clinical ethics: three perspectives on their potential to impact on end-of-life care. *Nursing Ethics* **21**(5): 621-626.
47. George M (2016) Stress in NHS staff triggers defensive inward-focussing and an associated loss of connection with colleagues: this is reversed by Schwartz Rounds. *Journal of Compassionate Health Care* **3**:9.
48. Gibson S (2008) Schwartz Center Rounds: Focusing on the Patient-Caregiver Relationship. *Health Progress*. **Nov-Dec 2008**: 40-43.
49. Gishen F, Wood M (2015) Using Schwartz Center Rounds to change hospice culture: does it work? *BMJ Supportive & Palliative Care,* **5**(1): 108 10.1136/bmjspcare-2014-000838.17. Abstract Only.
50. Gishen F Whitman S, Gill D, Barker R, Walker S. (2016) Schwartz Center Rounds: a new initiative in the undergraduate curriculum—what do medical students think? *BMC Medical Education* **16**:246.
51. Goodrich J (2011) Schwartz Center Rounds: Evaluation of the UK Pilots. The Kings Fund: London. Available from: https://www.kingsfund.org.uk/publications/schwartz-center-rounds-pilot-evaluation *(last accessed 15/2/18).*
52. Goodrich J (2014) Compassionate care and Schwartz Rounds: The nature of the work - Acknowledging it is hard. *Nurse Education Today*, **34**: 1185-87.
53. Hargreaves R (2015) "The value of Schwartz Rounds should not be underestimated" (comment) *Nursing Times* **111**(14):11.
54. Johal J (2015) Kindling Kindness for Compassionate Disaster Management. *PLOS Currents: Disasters* Available from: http://currents.plos.org/disasters/article/kindling-kindness-for-compassionate-disaster-management/ (last accessed 15/2/18).
55. Justice S, Rawles J, Brown J, Szczepanska S (2013) Will introducing 'Schwartz rounds' help foster a culture of compassionate care in the NHS? *Nursing Standard*, **27**(5)2: 26-27.
56. Kaplan, K (Powerpoint presentation): Leading Important Conversations in Healthcare: The Schwartz Center Rounds http://c.ymcdn.com/sites/www.odnetwork.org/resource/resmgr/2011_conf_ppts/g3_-_leading_important_conve.pdf (accessed 15/2/18).
57. Lee KJ, Forbes ML, Lukasiewicz GJ, Williams T, Sheets A, Fischer K, Niedner MF (2015) Promoting staff resilience in the pediatric intensive care unit. *Am J Crit Care*;**24**(5) 422-430 10.4037/ajcc2015720.
58. Lima J, Cavaliere R, Porensky E (2014) Closing the gap: Bereavement support for families, caregivers and medical staff. *Neuro-Oncology* **16**:178–183 doi:10.1093/neuonc/nou269.
59. Manning CF, Acker M, Houseman L, Pressman E, Goodman IF (2008) Schwartz Center Rounds: Evaluation Report (executive summary). Goodman Research Group Inc: Boston USA. Available from: http://www.theschwartzcenter.org/media/PTXAAE65CHR5UU4.pdf (Last accessed 15/2/18).
60. Moore CD, McCole Phillips J (2009) In These Rounds, Health-Care Professionals Heal Themselves. *Journal of Social Work in End-Of-Life & Palliative Care*, **5**(3-4): 116-125 DOI: 10.1080/15524250903555064.

For peer review only - http://bmjopen.bmj.com/site/about/guidelines.xhtml

61. Mudge B Stanzler M (2012) Schwartz Center Rounds® and Magnet®: Enhancing patient- and family-centered care American Nurse Today, 7(1) available from: https://www.americannursetoday.com/schwartz-center-rounds-and-magnet-enhancing-patient-and-family-centered-care/ Last accessed 1/3/17.
62. Mullick A, Wright A, Watmore-Eve J, Flatley M (2013) Supporting hospice staff: the introduction of Schwartz Center Rounds to a UK hospice setting. *European Journal of Palliative Care*, **20**(2): 62-65.
63. Pepper JR, Jaggar SI, Mason MJ, Finney SJ, Dusmet M (2012). Schwartz Rounds: reviving compassion in modern healthcare. *JRSM* **105:** 94–95.
64. Raphael-Grimm T (2008) Fostering compassionate patient care as an integrated, multidisciplinary outcome: Lessons learned from facilitating Schwartz Rounds. *International Journal for Human Caring*, 12(3):97.
65. Raso R (2009) Improving practice with Schwartz rounds; the DNP degree. *Nursing Management.* August 2009: 56.
66. Reed E, Cullen A, Gannon C, Knight A, Todd J (2015) Use of Schwartz Center Rounds in a UK hospice: Findings from a longitudinal evaluation. *J. Interprof. Care* **29**:365–366.
67. Rees-Adams D, Hughes L (2014) Introducing Schwartz Center Rounds into a Welsh NHS Health Board: six months in – a reflection on the process and experience to date. *Clinical Psychology Forum* **263**: 26-27 (*Special Issue of the British Psychological Society Clinical Psychology Forum on The Francis Report).*
68. Ryan B (2013) Schwartz Center Rounds are one way to boost compassion (Letter). *Nursing Standard* **27**(50):31.
69. Sanghavi D (2006) What makes for a compassionate patient-caregiver relationship? *Joint Commission Journal on Quality and Patient Safety* **32**(5):283-292.
70. Shield RER, Tong I, Tomas M, Besdine RW (2011) Teaching communication and compassionate care skills: An innovative curriculum for pre-clerkship medical students. *Medical Teacher* **33**: e408–e416.
71. Smyth M (2011) End of shift: goodbye to baby https://www.nurse.com/blog/2011/07/11/end-of-shift-goodbye-to-baby-2/ (last accessed 1/3/17).
72. Thompson A (2013) How Schwartz rounds can be used to combat compassion fatigue. *Nursing Management* 20(4):16–20.
73. Whitehead P (2015) The Impact of Schwartz Center Rounds on Moral Distress in a Large Healthcare System. *Journal of pain and symptom management* **49**(2):461. Abstract Only.
74. Wren B (2014) Schwartz Rounds: An intervention with potential to simultaneously improve staff experience and organisational culture. *Clinical Psychology Forum* **263** (*Special Issue of the British Psychological Society Clinical Psychology Forum on The Francis Report).*
75. Farr M, Barker R (2017) Can staff be supported to deliver compassionate care through implementing Schwartz Rounds in community and mental health services? *Qualitative Health Research*, 27(11): 1652-63.
76. Chadwick RJ, Muncer SJ, Hannon BC, Goodrich J, Cornwell J (2016) Support for compassionate care: Quantitative and qualitative evaluation of Schwartz Center Rounds in an acute general hospital. *JRSM Open*, 7(7): doi: 10.1177/2054270416648043
77. Babin BJ, Boles JS (1996) The effects of perceived co-worker involvement and supervisor support on service provider role stress, performance and job satisfaction. Journal of Retailing 72(1): p57.
78. Wren B. Schwartz Rounds: An intervention with potential to simultaneously improve staff experience and organisational culture. (Special Issue of the British Psychological Society Clinical Psychology Forum on The Francis Report). *Clinical Psychology Forum* 2014;**263**.
79. Maben J. Leamy M. Taylor C. Reynolds E. Shuldham C. Dawson J. McCarthy I. Ross S. Bennett L. (2018) *An Organisational guide: Understanding, implementing and sustaining Schwartz Rounds*® Copyright © 2018 King's College London Available from: https://www.surrey.ac.uk/SchwartzEvaluation

For peer review only - http://bmjopen.bmj.com/site/about/guidelines.xhtml

Table 1 Search results for each intervention

Intervention	Description of intervention	Database search result	Total excluded (duplicates; not eligible; full paper not available)	Papers from experts/ internet search	Total number included for review
Schwartz Center Rounds (Rounds)	Regular (usually monthly) open forum for staff in all positions. Organised and managed by a steering group, championed by a senior clinician, and facilitated – usually by a senior doctor and psychosocial practitioner. Last for one hour, with food provided. Multiple perspectives on a theme, scenario or patient case are briefly presented by a pre-arranged and pre-prepared panel and then opened to the audience for group reflection and discussion. Focus on non-clinical aspects (psychosocial, ethical, emotional issues) surrounding the patient-caregiver relationship. [*See Supplementary File 1 for a full definition*]	41	0	2	**43** **(33 non – empirical; 10 empirical)**
Action learning sets (ALS)	Based on the concept of learning by reflection on (or reviewing) an experience, ALS usually contains 4-6 members (peers), with (or without) a "set advisor" to facilitate the process. ALS tend to be held intermittently, over a fixed programme cycle, and most participants contract with the facilitator for an agreed length of time. They are often closed groups. The set is not a team, as the focus is on actions of individuals, rather than shared work objectives.	83	70 (8; 36; 26)	1	**14**
After action reviews (AAR)	AARs are facilitated meetings, led by a senior member of staff, which aim to encourage active reflection on performance following a specific event. An AAR is a one-off meeting post-event and includes those who were involved with the event. The focus is on gaps in performance, and what could be done differently to enhance the outcome. AARs generally last about 30 minutes.	76	74 (9; 64; 1)	0	**2**
Balint groups	Balint groups meet every 1-4 weeks for 1-3 years. In the group, typically a doctor presents a troubling patient incident while the group listens. The goal of the presentation is to understand the issue from both the patient's and doctor's perspectives. The presentation can last about ten minutes, after which group members can ask clarifying questions. When all questions are exhausted, the group is invited to imagine themselves in the roles of the doctor and the patient.	384	358 (170;151;37)	0	**26**
Caregiver support programme (CSP)	Originally developed for mental health/learning disability care homes, CSP is described as a theory-based social support intervention aimed at increasing exchanges of social support and decreasing negative social interaction. It consists of six 4-5-hour group training sessions (ten managers, ten direct-care staff and two facilitators) conducted over a nine week period. Strategies for improvement are drawn from the participants, based on their own experiences.	84	83 (10, 71, 2)	2	**3**

For peer review only - http://bmjopen.bmj.com/site/about/guidelines.xhtml

**Intervention**	**Description of intervention**	**Database search result**	**Total excluded (duplicates; not eligible; full paper not available)**	**Papers from experts/ internet search**	**Total number included for review**
Clinical supervision	Clinical supervision originated in psychotherapy but also adopted by other disciplines, e.g. psychology/nursing. Process described as identifying a key issue, describing and defining it, undertaking a critical analysis, examining solutions, formulating an action plan, implementation and evaluation. It can take five different forms: one-to-one with expert from same discipline; one-to-one with supervisor from different discipline; one-to-one with colleague of similar expertise; supervision between groups of colleagues working together, and network supervision between people who do not usually work together.	307	252<br>(42, 160, 50)	9	**64**
Critical incident stress debriefing (CISD)	In its original form, CISD is a single-issue debriefing session in a group context, led by an external team, following a traumatic event. CISD has seven phases: *introduction, fact, thought, reaction, symptom, teaching,* and *re-entry*. The debriefing session lasts for approximately 1.5-3 hours and takes place 24-72 hours after the traumatic event. The debriefing team is made up of a leader, a co-leader and a support, who work in conjunction to support the participants and to allow them to feel safe.	388	386<br>(62; 248; 76)	0	**2**
Mindfulness-based stress reduction (MBSR)	The central principle of MBSR is mindfulness - being focused on and aware of the present moment with a non-judging attitude of acceptance. The original training module is eight weeks long with weekly sessions of 2.5 hours each. There is a seven hour session which takes place between weeks six and seven, and participants are asked to complete 45 minutes of daily formal mindful practice. They are taught a variety of mindful meditative practices, and there are group discussions about the application of these practices.	127	110<br>(13; 72; 25)	0	**17**
Peer-supported storytelling	Narrative storytelling is the act of an individual recounting verbally to one or more people a plausible account of an event, or series of events, possessing narrative truth for the teller. The story is arranged in a time sequence with plot, characters, context, intentionality, and perspective taking, possibly including the teller's actions, thoughts and feelings.	4	3<br>(3; 0; 0)	0	**1**
Psychosocial intervention training	Psychosocial intervention training involves cognitive behavioural approaches for managing symptoms, understanding symptom-related behaviour, relationship formation and helping service users to cope with symptoms. Teaching sessions are supplemented by small group supervision. Students are required to provide brief case study presentations about service users they are working with and receive feedback. Early courses were developed for nurses but quickly became multidisciplinary.	37	35<br>(6; 25; 4)	1	**3**
Reflective practice groups (RPG)	RPGs are facilitated groups of about ten healthcare professionals or students in which participants share and explore professional, clinical, ethical, and personal insights arising from their clinical work or training. RPGs are ongoing, convening regularly with each group lasting for about one hour. Discussion topics can either be raised by the facilitator or by the participants. The discussion is meant to be supportive as well as challenging, encouraging consideration of alternative viewpoints.	91	83<br>(8; 73; 2)	0	**8**

For peer review only

For peer review only - http://bmjopen.bmj.com/site/about/guidelines.xhtml

Intervention	Description of intervention	Database search result	Total excluded (duplicates; not eligible; full paper not available)	Papers from experts/ internet search	Total number included for review
Resilience training	Resilience training is in part based on CBT theories and in its original form is a manualised intervention comprising 18 hours of workshops. The key characteristics include delivery to groups of practitioners who support one another and facilitated by an expert in personal and professional transition supervision. University of Pennsylvania well-known example consists of: learning ways to challenge unrealistic negative beliefs, strengthening problem solving, adopting assertiveness and negotiation skills, improving ability to deal with strong feelings, and learning how to tackle procrastination through use of decision-making and action-planning tools.	144	138<br>(36, 72, 30)	0	**6**
**TOTAL**		**1725**	**1592**<br>**(367; 972; 253)**	**13**	**146**

For peer review only - http://bmjopen.bmj.com/site/about/guidelines.xhtml

Table 2: Schwartz Rounds empirical evaluations: data extracted from included papers (Short legend: Schwartz Rounds Included Studies)

Authors	Setting	Aims/purpose	Design/methodology	Measures	Main findings	Quality
Corless et al (2009)[39]	Educational<br><br>USA	Development, implementation and evaluation of Educational Rounds for interdisciplinary graduate students to help them learn empathy, self-reflection and moral courage	Quantitative post-Round evaluation survey<br><br>Graduate students. Over a 4-years comprising 11 Rounds (n=329 individual evaluations)	Survey included 7 statements about Rounds (according to agreement on a 5-point Likert scale) plus an overall rating of the quality of the Round they are evaluating.	**Overall**<br>High support and satisfaction with Rounds (e.g. 86% rated Rounds as excellent or exceptional).<br>67% stated intention to attend future Rounds (range 57-93% for individual Rounds). Lowest intention from a Round presented by lab scientists. Highlighted importance of topic to encourage attendance.	Low/Moderate (lack of clarity e.g. sampling, measures, not all data presented).
Manning et al (2008)[59]<br>Lown et al (2010)[24]	Hospitals<br><br>USA	To assess the impact of Rounds e.g. changes in attendees behaviours and beliefs about patient care, teamwork, stress and personal support	Mixed method evaluations<br><br>- Retrospective survey of 256/413 (62%) attenders at 6 experienced Rounds sites (offering Rounds for 3+years) plus 44 interviews with providers, Rounds leaders, facilitators and hospital administrators.<br><br>- Prospective pre-post web-based survey of 222/399 (56%). Rounds attenders from 10 hospitals newly implementing Rounds (had held 7 or more Rounds)	Study-specific (non-standardised) - some adapted from published measures) Likert scale measures to investigate:<br>1) insights into psychosocial and emotional aspects of clinical care on patient interactions (15 items)<br>2) teamwork (9 items)<br>3) support for providers (number of items not mentioned)	**Overall**<br>Found "dose" effect: more rounds attended, more impact they have.<br>**Self**<br>Attendance at Rounds associated with decreased stress and improved ability to cope with psychosocial demands/emotional difficulties at work.<br>**Others**<br>Rounds attendance led to increased patient interaction and teamwork scores. Interviews highlighted benefits including: getting to know colleagues and putting themselves in their shoes, and an improved sense of connection/shared purpose.<br>**Organisation**<br>Both samples (51% retrospective; 40% prospective) reported changes in practices/polices including: culture change (dialogue that doesn't happened elsewhere); focus on patient-centred care; practice changes (e.g. increased/earlier palliative care use)	**Quantitative:** Moderate (lack of control group of non-attenders and used non-standardised measures).<br>**Qualitative:** Moderate (limited reporting of theoretical underpinnings and strategies to improve rigour e.g. deviant case analysis)<br>**Mixed methods:** low (limited reporting of mixed methods rationale/features)
Goodrich (2011)[51]<br>Goodrich (2012)[25]	Two hospitals<br><br>UK	Pilot study to evaluate introduction of Rounds to UK in 2 hospital sites.	Mixed methods evaluation over 2 year period:<br><br>1) Pre-post pilot surveys<br><br>2) Evaluation forms from each Round (quality,	Used same questionnaires as Lown et al. (2010)[24]	**Overall**<br>Majority (86% site A, 78% site B) rated rounds as excellent/good.<br>**Self**<br>- Increases (pre-post) in:<br>- confidence in handling sensitive issues<br>- beliefs in the importance of empathy	**Quantitative:** Moderate (limitations in measures used and lack of control group)<br><br>**Qualitative:** Moderate (low reporting of

For peer review only - http://bmjopen.bmj.com/site/about/guidelines.xhtml

Authors	Setting	Aims/purpose	Design/methodology	Measures	Main findings	Quality
			logistics, demographics, plans to attend future Rounds). Each site held 10 Rounds (n=301 attenders site A, 74% completed evaluation form; n=949 at site B, 69% completed evaluation form).<br><br>3) Qualitative interviews: Experience of attenders, steering group, panellist, facilitators (n=23). Second interview at end with n=13.		- confidence in handling non-clinical aspects of care<br>Also reported feeling less stressed and less isolated in their work.<br>Interview findings: increased compassion, reduced stress<br>**Others**<br>Increases (pre-post) in:<br>- actual empathy with patients<br>- openness to expressing thoughts, questions and feelings about patient care with colleagues<br>Interview findings: greater respect/empathy for colleagues, better teamwork/collaboration<br>**Organisation**<br>Interview findings:<br>- Board/senior support important<br>- Wider impacts: reduced hierarchy, help build shared values/support strategic vision.	strategies to improve rigour and theoretical underpinnings)<br><br>**Mixed methods:** low (limited reporting of mixed methods rationale/features)
Reed et al (2015)[66]	Hospice<br><br>UK	Evaluate the impact of Rounds on staff and the organisation	Longitudinal mixed methods evaluation (1 year): survey and focus groups<br><br>Exit survey: 398/535 (74%) attendees)<br><br>4 interprofessional focus groups (n=33, including attendees, non-attendees and presenters)	5-point Likert scale assessing:<br>Topic relevance, knowledge gained, impact on individual, facilitation and working relationships<br><br>(*Similar questions to Lown & Manning, 2010*[24]*/Goodrich, 2011*[51]).	**Overall**<br>78% rated Rounds as excellent or exceptional<br>**Self**<br>- Focus groups:<br>- Validation of experiences<br>- Honesty, openness and vulnerability allowed others to see person on human level<br>**Others**<br>87% gained insight into how others think/feel in caring for patients<br>- Focus groups: fostered understanding of importance of non-clinical staff contribution<br>- BUT non-attenders felt responsibility to smooth running of hospice and felt they contributed to wider team without needing to hear stark realities of care/work.<br>**Organisation**<br>- Focus groups: more connected, shared purpose	**Quantitative**: Moderate, (non-validated measures and lack of control group)<br>**Qualitative:** Moderate (lack of elements of rigour in qualitative component e.g. reflexivity, contradictory/ deviant cases other than non-attenders)<br>**Mixed methods:** low (limited reporting of mixed methods rationale/features)
Deppoliti et al (2015)[44]	Hospital<br><br>USA	- Learn why people attend Rounds<br>- Understand what is gained from the experience<br>- Identify key	Qualitative: 4 focus groups (n=27) and 3 telephone interviews<br><br>Purposive sampling of attenders by steering group to represent those that were active contributors and	N/A	**Overall**<br>Rounds viewed as beneficial.<br>**Self**<br>- impact on behaviour/attitudes "think differently"<br>**Others**<br>- exposing emotions (increased appreciation, awareness and sensitivity of what others in the healthcare team experience)	Qualitative: High (good reporting and transparency in methods of data collection and analysis including e.g. independent researcher coding/analysis, third party review of findings

For peer review only - http://bmjopen.bmj.com/site/about/guidelines.xhtml

Authors	Setting	Aims/purpose	Design/methodology	Measures	Main findings	Quality
		elements to use in measuring effectiveness	included range of roles/professions and frequency (low and high attenders).		- walking in another's shoes (empathic awareness)<br>**Organisation**<br>- culture change (strong message that staff matter; values/beliefs/ norms evolved positively; not about productivity; improved teamwork due to level playing field).<br>**Other findings:**<br>- inequality of topics (some topics more than others lead to increased learning, growth)<br>- influence of rules and boundaries (spoken/unspoken rules about what is acceptable to share)<br>*Suggested improvements:*<br>- providing list of upcoming topics so staff can plan attendance<br>- providing anonymised method to contribute (eg Qs on cards)	and use of illustrative quotations)
George (2016)[47]	Hospital<br><br>UK	To examine the impact of Schwartz Rounds on staff wellbeing and patient care	Mixed methods<br><br>Interviews with staff (nurses and HCAs) about stress (n=11, 10 were female, 10 were white British)<br><br>Key themes extracted using grounded theory ➔ development of a new measure administered at the beginning and end of 2 Rounds (n=55 forms completed) Mostly female, white and only 2 were over 59yrs old.	The Organisational Response to Emotions Scale (ORES) (investigator-designed): 9 scales<br><br>Analysis controlled for whether it was first ever Round, length of time in role, session attended.	**Self**<br>- Emotional Labour: significantly reduced in staff where pre-round was their first round.<br>- Self-reflection increased pre-post<br>Compared SCR attenders with 10/11 interviewees who also completed ORES (did not attend Rounds). Found non-attenders had higher burnout and emotional labour, and more negative appraisal of organisation.<br>**Others**<br>- More negative appraisal of line manager	**Quantitative:** Moderate (small biased sample, lack of control group, measure based on limited staff group input (nurses/HCA only).<br><br>**Qualitative:** Moderate (limited reporting of elements of rigour e.g. audit trail, theoretical saturation)<br><br>**Mixed methods:** high (good reporting of mixed methods)
Shield et al (2011)[70]	Medical school<br><br>USA	To improve communication skills, they designed "Schwartz Communication Sessions" Aimed to provide medical students with the	Quantitative: evaluation form (both quant and qual/content analysis).<br><br>Sampling is unclear (a sample of 92, 99 and 94 are reported) but report having evaluation forms from 66-95% of students for all three sessions (71-80% for two	Not specified (but appear similar to Lown & Manning, 2010)[24]	**Overall**<br>93% of faculty and 83% of students rated the sessions as good, excellent or exceptional<br>**Self**<br>80% of students and 96% of faculty believe students gained knowledge that will help them care for patients<br>**Others**<br>75% of students and 96% of faculty believe the sessions will help students communicate better with patients and family members	Moderate (lack of clarity regarding sampling/sample and measures)

For peer review only - http://bmjopen.bmj.com/site/about/guidelines.xhtml

Authors	Setting	Aims/purpose	Design/methodology	Measures	Main findings	Quality
		rationale and proficiency for effective communication.	sessions) and Faculty members (n=24) response rate 42-92%			
Gishen et al (2016)[50]	Medical school<br><br>UK	Examine the potential of Rounds within the undergraduate curriculum	Mixed method evaluation<br><br>2 student-focused Rounds were piloted at a medical school (1 Round each for year 5 and 6 students)<br><br>Evaluation questionnaire immediately following the Rounds:<br>258/334 (77%) year 5 students attended the Round and 247 (94%) responded.<br><br>180/343 (52%) year 6 students attended the Round and 126 (70%) responded.<br><br>Focus group (n=7 year 5 students) to explore student views on the Round	Feedback form from the Point of Care Foundation; plus free text comments. Questions either Yes/No or 5-point Likert rating scale (1= poor to 5 = exceptional)	**Overall**<br>Mean student ratings of a session were 3.5/5 (year 5) and 3.3/5 (year 6)<br>- 81% agreed/strongly agreed the presentation of cases was helpful<br>- 80% would attend a future Round<br>- 64% agreed Rounds should be integrated into the curriculum<br><br>Focus group finding:<br>Feelings about the Round (response to round, size of audience- large inhibiting, positive comparison to current reflective practice; post event peer discussions)<br>**Self**<br>- 69% year 5 vs 87% year 6 students were worried about compassion fatigue or burnout<br>- 92% agreed/strongly agreed that they appreciated hearing stories demonstrating human side of medicine<br>- Focus group finding: Psychological aspects of SCR (psychological pressures of medicine, how session encouraged positive processing of emotion, sharing personal stories between health professionals).<br>**Others**<br>82% agreed/strongly agreed that attending Round gave insight into how others feel/think about caring for patients	Quantitative: Moderate due to convenience sampling approach and lack of control group<br><br>Qualitative: Moderate due to limited reporting of measures taken to enhance rigour<br><br>Low for mixed methods reporting.
Farr and Baker (2016)[75]	Community and mental health services<br><br>UK	To investigate how Schwartz Rounds are implemented and how they support staff in mental health and community services	Mixed methods: Realist evaluation involving 3 case studies including 22 interviews, 5 observations and descriptive analysis of evaluation sheets as secondary data.<br><br>Key themes extracted using	Topic guides developed from a previous evaluation of Schwartz Rounds Goodrich, 2012[4]<br><br>Process data collected through	**Overall**<br>Rounds had the potential for successful implementation in these settings.<br>**Self**<br>More mindful of emotional impact of work<br>**Others**<br>Positive impact on relationships with patients and other staff (e.g. better communication, increased trust)<br>**Organisation**	**Qualitative:** Moderate (methods for realist methods and analysis lack clarity; e.g. unclear presentation of Context-Mechanism-Outcome configurations)<br>**Quantitative**: Low (descriptive data on

For peer review only - http://bmjopen.bmj.com/site/about/guidelines.xhtml

Authors	Setting	Aims/purpose	Design/methodology	Measures	Main findings	Quality
			framework analysis, then used to track context, mechanism outcome configurations	evaluation sheets i.e attendance, types of participants and perspectives	Rounds can positively influence wider culture of the organisation (e.g. prompt introduction of other forms of staff support)<br>**Other findings:**<br>Facilitators and barriers to implementation are identified including that strong leadership is a crucial element in success	attenders only; lack of clarity on analysis of evaluation sheet data and integration with interview data).<br>**Mixed methods**: Low (limited reporting of mixed methods rationale/features)
Chadwick et al (2016)[76]	Hospital<br><br>UK	To assess the perceived impact of Schwartz Rounds™ on hospital staff over a 3 year period	Mixed methods analysis of evaluation forms<br><br>795 participant evaluation forms analysed quantitatively and qualitatively<br><br>Key themes extracted using thematic analysis<br>Quantitative (scale) data analysed descriptively and inferentially (e.g. for differences in scores for different items/scales).	Standard evaluation form, eight statements rated on five-point scale and free text comments.	**Overall**<br>All aspects of Rounds highly rated by staff<br>**Self**<br>Improved reflection on own experiences<br>**Others**<br>Improved insight and understanding of others and their roles and support within the organization<br>Most agreed that attended helped caring for patients and work with colleagues<br>**Organisation**<br>Reducing the effect of 'silo working'<br>Staff were given a space where powerful emotions were accepted and responded to constructively<br>Need to make Rounds more accessible to wider cross section of staff<br>**Other findings:**<br>- No significant differences between disciplines/staff groups in survey ratings	**Qualitative:** High<br>**Quantitative:** Moderate (statistical methods unclear; lack of account of impact of sample size on outcomes of analysis)<br>**Mixed Methods:** Not a mixed methods study, just in analysis of evaluation data therefore criteria n/a

For peer review only - http://bmjopen.bmj.com/site/about/guidelines.xhtml

Table 3 Features of Rounds compared/contrasted with comparative interventions (Short Legend: Comparative Analysis)

		**Intervention (see footnote for full label)**										
	**Feature of Rounds**	**ALS**	**AAR**	**Balint**	**Care**	**Super**	**CISD**	**Mind**	**Story**	**Psych**	**Refl**	**Resil**
1	Share challenging/rewarding experiences about delivering patient care	May Not	No	Yes	May Not	Yes	Yes	No	May Not	Yes	Yes	May Not
2	Focus on psychosocial and emotional issues of patient-caregiver relationships	May Not	No	Yes	May Not	May Not	May Not	No	May Not	May Not	May Not	May Not
3	Provides an explicit opportunity for reflection	Yes	Yes	Yes	May Not	Yes	Yes	Yes	Yes	Yes	Yes	May Not
4	Open, honest communication	Yes	Yes	Yes	Yes	Yes	Yes	Yes	Yes	Yes	Yes	May Not
5	Provides an opportunity to give and/or receive peer support	Yes	May Not	Yes	Yes	Yes (if group)	Yes	Yes	Yes	No	Yes	Yes
6	Telling and hearing stories related by theme, scenario or patient case	No	Yes	No	May Not	No	No	No	Yes	No	No	No
7	Ongoing programme (vs. one-off)	No	No	Yes	No	Yes	No	No	No	No	Yes	No
8	Time-fixed session (vs. flexible length/unspecified)	No	No	Yes	Yes	Yes	No	Yes	No	Yes	No	Yes
9	Planned provision of food/refreshments	No	No	No	No	No	No	No	No	No	No	No
10	Open to all/any clinical and non-clinical staff	No	Yes	No	No	No	No	No	No	No	No	May Not
11	All levels of staff/intended to flatten hierarchy	Yes	Yes	No	Yes	No	No	Yes	Yes	Yes	No	Yes
12	Open group membership (vs. closed/invited members only)	No	No	No	No	No	No	No	No	No	No	No
13	Multidisciplinary	May Not	Yes	May Not	Yes	No	May Not	Yes	May Not	Yes	No	May Not
14	Pre-prepared/rehearsed stories or focus	Yes	No	No	No	No	No	No	Yes	No	No	No
15	Facilitated discussions	May Not	Yes	Yes	Yes	Yes	Yes	Yes	No	Yes	Yes	Yes

For peer review only

For peer review only - http://bmjopen.bmj.com/site/about/guidelines.xhtml

	Feature of Rounds	Intervention (see footnote for full label)										
		**ALS**	**AAR**	**Balint**	**Care**	**Super**	**CISD**	**Mind**	**Story**	**Psych**	**Refl**	**Resil**
16	Panel presenters tell stories giving their perspectives on a theme, scenario or patient case	No	No	No	No	No	No	No	No	No	No	No
17	Group intervention	Yes	Yes	Yes	Yes	May Not	Yes	Yes	No	Yes	Yes	Yes
18	Organisational support: senior doctor/clinician champions	May Not	Yes	Yes	Yes	Yes	May Not	No	No	No	No	No
19	Safe and confidential environment	Yes	Yes	Yes	Yes	Yes	Yes	Yes	Yes	Yes	Yes	Yes

Features that define what Rounds are 'not'		Intervention										
		**ALS**	**AAR**	**Balint**	**Care**	**Super**	**CISD**	**Mind**	**Story**	**Psych**	**Refl**	Resil
1	Problem-solving	Yes	Yes	Yes	Yes	Yes	May Not	No	No	Yes	No	Yes
2	Production of actionable outputs	Yes	Yes	No	No	May Not	No	No	No	No	No	No
3	Flexibility in format (versus licenced/contract[1])	Yes	Yes	Yes	May Not	Yes	Yes	Yes	Yes	No	Yes	May Not
4	Focus on clinical aspects of patient care, their diagnosis or plan of care	May Not	May Not	No	May Not	May Not	May Not	No	May Not	Yes	May Not	May Not

*Manualised but adapted to experiences of group

ALS= Action Learning Sets; AAR= After Action Reviews; Balint= Balint Groups; Care=Caregiver Support Programme; Super=Clinical/Restorative Supervision; CISD= Critical Incident Stress Debriefing; Mind= Mindfulness-based stress reduction; Story=Peer supported story-telling; Psych= Psychosocial Intervention Training; Refl= Reflective Practice Groups; Resil=Resilience Training.

[1] Licenced/contract – fidelity to original intervention – i.e. one model/approaches or many, degree of flexibility offered.

For peer review only
For peer review only - http://bmjopen.bmj.com/site/about/guidelines.xhtml

Supplementary file 1: Composite definition of Rounds (key features shown in bold)

"Schwartz Rounds are the signature programme of the Schwartz Center for Compassionate Care. They provide a **regular** (usually monthly) **open forum** (drop-in rather than by invitation) for **multidisciplinary** clinical and non-clinical **staff at all positions** within the healthcare organisation to come together in an environment that is intended to provide a **safe and confidential space**. They are intended to provide staff with a **level playing field** to **reflect** upon, explore and tell stories about the difficult, **challenging and rewarding experiences they face when delivering patient care**, and **receive the support of their colleagues**. Rounds are typically organised and managed by a steering group, **championed by a senior doctor/clinician**. They **last for one hour** and are often held during lunch periods (with **food provided**). They are a **group intervention** within which **multiple perspectives on a theme, scenario or patient case** (i.e. their stories) are briefly presented by a **pre-arranged and pre-prepared panel** and then opened to the audience for group reflection and discussion, usually **facilitated** by a senior doctor and psychosocial practitioner. The **focus is on the non-clinical aspects (e.g. psychosocial, ethical and emotional issues) surrounding the patient-caregiver relationship** – thereby addressing a wide range of important topics rarely discussed elsewhere - and the attendees are encouraged to be **open and honest** and reflect, discuss and explore their experiences thoughts and feelings. The interaction between the panellists and audience is felt to foster insight and support from colleagues, create a sense of working in a supportive environment and lead to improved relationships and communication within the hospital hierarchy, improved communication and teamwork between staff and patients and among staff, improved wellbeing of staff including enhanced resilience, improved compassionate care and ultimately impact on organisational culture."

Some sources explicitly articulated what Rounds were ***not***. This included: that aims were distinct from traditional clinical/grand rounds; that is stories and discussions should **not focus**

For peer review only

on the patient, their diagnosis or plan of care; that Rounds should not be used for **problem solving**; and that they are **not intended to produce actionable outputs**.

For peer review only

Supplementary file 2: References for included papers from the scoping reviews of comparable interventions

Action Learning Sets

Bailie L, Bromley B, Walker M, Jones R, Mhlanga F (2014) Implementing service improvement projects within pre-registration nursing education: A multi-method case study evaluation. *Nurse Education in Practice*, 14(1): 62-68.

Booth A, Sutton A, Falzon L (2003) Working together: supporting projects through action Learning, *Health Information and Libraries Journal*, 20: **2**25–231.

Bourner T & Frost P (1996) Experiencing action learning. *Employee Counselling Today*, 8(6): 11–18.

Burgess H & Carpenter J (2008) Building capacity and capability for evaluating the outcomes of social work education (the OSWE project): Creating a culture change. *Social Work Education*, 27(8): 898-912.

Currie K, Biggam J, Palmer J, Corcoran T (2012) Participants' engagement with and reactions to the use of on-line action learning sets to support advanced nursing role development. *Nurse Education Today* 32: 267–272.

Douglas S & Machin T (2004) A model for setting up interdisciplinary collaborative working in groups: lessons from an experience of action learning. *Journal of psychiatric and mental health nursing*, 11: 189–193.

Eisen S, Sukhani S, Brightwell,A, Stoneham S, Long A (2013) Peer mentoring: evaluation of a novel programme in paediatrics, *Arch. Dis. Child.* 0:1–5.

Lamont S, Brunero S, Russell R (2010) An exploratory evaluation of an action learning set within a mental health service. *Nurse Education in Practice*, 10: 298-302.

Leggat S, Balding C and Anderson J. (2011) Empowering health-care managers in Australia: an action learning approach, *Health Services Management Research*, 24: 196–202.

Machin A & Pearson P (2014) Action learning sets in a nursing and midwifery practice learning context: A realistic evaluation. *Nurse Education in Practice* 14: 410-416.

Mann R, Ball K, Watson G (2011) Mentoring for NHS general practitioners: a prospective pilot study of an action learning approach, *Educ Prim Care,* 22(4): 235-240.

Moore L (2007) Partnerships and work-based learning: an evaluation of an opportunity to pioneer new ways to care for the older people in the community, *Assessment & Evaluation in Higher Education*, 32(1): 61-77.

Phillips S, Tapping J, Ooms A, Marks-Maran D, Godden R (2013) A preceptorship programme for health visitors and school nurses: a pilot study *Community Practitioner*, 86(1): 18-22.

Rivas K & Murray S (2010) Our shared experience of implementing action learning sets in an acute clinical nursing setting: approach taken and lessons learned. *Contemporary Nurse*, 35 (2): 182-7.

For peer review only

After Action Reviews

Reiter-Palmon R, Kennel V, Allen JA, Jones KJ, Skinner AM (2015) Naturalistic decision making in after-action review meetings: The implementation of and learning from post-fall huddles *Journal of Occupational and Organizational Psychology*, 88(2): 322–340.

Walker J, Andrews S, Grewcock D, Halligan A (2012) Life in the slow lane: making hospitals safer, slowly but surely. *Journal of the Royal Society of Medicine*, 105: 283–287.

Balint Groups

Abeni MS, Magni M, Conte S, Mangiacavalli L, Pochintesta G, Vicenzi VV, Ferretti A, Pompa F, Cocito C, Klersy, Corso C (2014) Psychological care of caregivers, nurses and physicians: A study of a new approach. *Cancer Medicine* 3(1): 101-110.

Adams KE, O'Reilly M, Romm J, James K (2006) Effect of Balint training on resident professionalism. *American Journal of Obstetrics & Gynecology* 195(5): 1431-1437.

Airagnes GSM, Consoli O, De Morlhon AM, Galliot C, Lemogne, Jaury P (2014) Appropriate training based on Balint groups can improve the empathic abilities of medical students: A preliminary study. *Journal of psychosomatic research* 76(5): 426-429.

Dahlgren MA, Almquist A, Krook J (2000) Physiotherapists in Balint group training. *Physiotherapy Research International* 5(2): 85-95.

Dokter HJ, Duivenvoorden HJ, Verhage HF (1986) Changes in the attitude of general practitioners as a result of participation in a Balint group. *Family practice* 3(3): 155-163.

Fitzgerald G & Hunter MD (2003) Organising and evaluating a Balint group for trainees in psychiatry. *Psychiatric Bulletin* 27(11): 434-436.

Johnson AH, Brock CD, Hamadeh G, Stock R (2001) The current status of Balint groups in US family practice residencies: A 10-year follow-up study, 1990-2000. *Family medicine* 33(9): 672-677.

Johnson AH, Brock CD, Hueston WJ (2003) Resident physicians who continue Balint training: a longitudinal study 1982-1999. *Family medicine* 35(6): 428-433.

Johnson AH, Nease DE, Milberg LC, Addison RB (2004) Essential characteristics of effective Balint group leadership. *Family medicine* 36(4): 253-259.

Kjeldmand D & Holmstrom I (2008) Balint groups as a means to increase job satisfaction and prevent burnout among general practitioners. *Annals of Family Medicine* 6(2): 138-145.

Kjeldmand D. and Holmstrom I (2010). Difficulties in Balint groups: a qualitative study of leaders' experiences. *British Journal of General Practice* 60(580): 808-814.

Kjeldmand D, Holmstrom I, Rosenqvist U (2004) Balint training makes GPs thrive better in their job. *Patient education and counseling* 55(2): 230-235.

Kjeldmand D, Holmstrom I, Rosenqvist U (2006) How patient-centred am I? A new method to measure physicians' patient-centredness. *Patient Education & Counseling* 62(1): 31-37.

Lee E & Kealy D (2014). Revisiting Balint's innovation: enhancing capacity in collaborative mental health care. *Journal of Interprofessional Care* 28(5): 466-470.

For peer review only

Leggett A (2012). Transcultural issues in the dynamics of a Balint clinical reflection group for community mental health workers. *Transcultural Psychiatry* 49(2): 366-376.

Levenstein S (1978). A report of three years' experience of a Balint group in Cape Town. *South African Medical Journal*, 54(3): 121-123.

Levenstein S. (1981). An undergraduate Balint group in Cape Town. *South African Medical Journal*. 59(18): 642-643.

Levenstein S (1982). An undergraduate Balint group in Cape Town - A follow-up report. *South African Medical Journal* 62(3): 89-90.

Margalit AP, Glick SM, Benbassat J, Cohen A (2004) Effect of a biopsychosocial approach on patient satisfaction and patterns of care. *Journal of General Internal Medicine* 19(5 Pt 2): 485-491.

Nielsen HG & Tulinius C (2009) Preventing burnout among general practitioners: is there a possible route? *Education for Primary Care* 20(5): 353-359.

Salander Pä, Sandström M (2014) A Balint-inspired reflective forum in oncology for medical residents: Main themes during seven years. *Patient Education & Counseling* 97(1): 47-51.

Samuel O (1989) How doctors learn in a Balint group. *Family practice* 6(2): 108-113.

Sekeres MA, Chernoff M, Lynch TJ, Kasendorf EI, Lasser DH, Greenberg DB (2003) The impact of a physician awareness group and the first year of training on hematology-oncology fellows. *Journal of Clinical Oncology* 21(19): 3676-3682.

Smith M & Anandarajah G (2007) Mutiny on the balint: balancing resident developmental needs with the balint process. *Family medicine* 39(7): 495-497.

Stojanovic-Spehar S, Blazekovic-Milakovic S, Matanic D (2004) Education about pharmacotherapy and psychotherapy of anxiety among primary care physicians in Croatia: Balint group approach, *Croatian medical journal* 45(5): 625-629.

Torppa MA, Makkonen E, Martenson C, Pitkala KH (2008) A qualitative analysis of student Balint groups in medical education: contexts and triggers of case presentations and discussion themes. *Patient Education & Counseling* 72(1): 5-11.

Yakeley J, Shoenberg P, Morris R, Sturgeon D, Majid S (2011) Psychodynamic approaches to teaching medical students about the doctor-patient relationship: Randomised controlled trial. *Psychiatrist* 35(8): 308-313.

Caregiver Support Program

Heaney C (1991) Enhancing Social Support at the Workplace: Assessing the Effects of the Caregiver Support Program *Health Educ Behav* 18(4); 477-494.

Heaney C, Price RH, Rafferty J (1995) Increasing Coping Resources at Work: A Field Experiment to Increase Social Support, Improve Work Team Functioning, and Enhance Employee Mental Health. *Journal of Organizational Behavior*, 16 (4): 335-352.

Heaney CA, Price RH, & Rafferty J (1995) The caregiver support program: An intervention to increase employee coping resources and enhance mental health. In L. R. Murphy, J. J. J. Hurrel, S. L. Sauter, & G. P. Keita (Eds.), *Job stress interventions* (pp. 93-108). Washington, DC: American Psychological Association.

For peer review only

Clinical and Restorative Supervision

Primary studies:

Arvidsson B, Lofgren H, Fridlund B (2001) Psychiatric nurses' conceptions of how a group supervision programme in nursing care influences their professional competence: a 4-year follow-up study. *Journal of Nursing Management*, 9(3): 161-171.

Ardvidsson B, Skarsater I, Oijervall J, Fridlund B (2008) Process-oriented group supervision implemented during nursing education: nurses' conceptions 1 year after their nursing degree. *Journal of Nursing Management*, 16: 868–875.

Ashmore R, Carver N, Clibbens N, Sheldon J (2012) Lecturers' accounts of facilitating clinical supervision groups within a pre-registration mental health nursing curriculum. *Nurse Education Today* 32: 224-228.

Begat IBE, Severinsson, EI (2001) Nurses' reflections on episodes occurring during their provision of care-An interview study. *International Journal of Nursing Studies*, 38: 71-77.

Bogo M, Paterson J, Tufford L, King R (2011) Interprofessional clinical supervision in mental health and addiction: Toward identifying common elements. *The Clinical Supervisor*, 30:1, 124-140.

Bondas T (2010) Nursing leadership from the perspective of clinical group supervision: A paradoxical practice. *Journal of Nursing Management*, 18: 477-486.

Bradshaw T, Butterworth A, Mairs H (2007) Does structured clinical supervision during psychosocial intervention education enhance outcome for mental health nurses and the service users they work with? *Journal of Psychiatric and Mental Health Nursing*, 14: 4-12.

Brunero S, Lamont S (2012) The process, logistics and challenges of implementing clinical supervision in a generalist tertiary referral hospital. *Scand J Caring Sci*; 26; 186–193.

Buus N, Angel S, Traynor M, Gonge H (2010) Psychiatric hospital nursing staff's experiences of participating in group-based clinical supervision: An interview study. *Issues in Mental Health Nursing*, 31:10, 654-661.

Danielsson A, Sundin-Andersson C, Hov R, Athlin E (2009) Norwegian and Swedish preceptors' views of their role before and after taking part in a group supervision program. *Nurs Health Sci*, 11(2):107-113.

Davey B, Desousa C, Robinson S, Murrells T (2006) The policy--practice divide: Who has clinical supervision in nursing? *Journal of Research in Nursing*, 11(3): 237-248.

Davys AM & Beddoe L (2009) Interprofessional learning for supervision: 'taking the blinkers off'. *Learning in Health and Social Care*, 8(1): 58-69.

Edwards D, Burnard P, Hannigan B, Cooper L, Adams J, Juggessur T, Fothergil A, Coyle D (2006) Clinical supervision and burnout: The influence of clinical supervision for community mental health nurses. *Journal of Clinical Nursing* 15, 1007–1015.

Edwards D, Cooper L, Burnard P, Hannigan B, Adams J, Fothergill A, Coyle D (2005) Factors influencing the effectiveness of clinical supervision. *Journal of Psychiatric and Mental Health Nursing*, 12: 405-414.

For peer review only

Flackman B, Fagerberg I, Haggstrom E, Kihlgren A, Kihlgren M (2007) Despite shattered expectations a willingness to care for elders remains with education and clinical supervision. *Scand J Caring Sci*, 21: 379-389.

Gonge H & Buus N (2011) Model for investigating the benefits of clinical supervision in psychiatric nursing: A survey study. *International Journal of Mental Health Nursing*, 20: 102-111.

Gonge H & Buus N (2010) Individual and workplace factors that influence psychiatric nursing staff's participation in clinical supervision: a survey study and prospective longitudinal registration. *Issues in Mental Health Nursing*, 31:5,345-354.

Hallberg IR (1995) Clinical group supervision and supervised implementation of planned individualized care of severely demented people: effects on nurses, provision of the care, and patients. *Journal of Psychiatric and Mental Health Nursing*, 2: 111-114.

Hallberg IR (1994) Systematic clinical supervision in a child psychiatric ward: Satisfaction with nursing care, tedium, burnout, and the nurses' own report on the effects of it. *Archives of Psychiatric Nursing*, 8(1): 44-52.

Hall-Lord M, Theander K, Athlin E (2013) A clinical supervision model in bachelor nursing education -- Purpose, content and evaluation. *Nurse Education in Practice*, 13: 506-511.

Hansebo G & Kihlgren M. Nursing home care: changes after supervision. *Journal of Advanced Nursing* 45(3), 269–279.

Heaven C, Clegg J, Maguire P (2006) Transfer of communication skills training from workshop to workplace: The impact of clinical supervision. *Patient Education and Counseling* 60, 313–325.

Holmlund K, Lindgren B, Athlin E (2010) Group supervision for nursing students during their clinical placements: its content and meaning. *Journal of Nursing Management*, 18, 678–688.

Hyrkas K (2005) Clinical supervision, burnout, and job satisfaction among mental health and psychiatric nurses in Finland. *Issues in Mental Health Nursing*, 26:5, 531-556.

Hyrkas K, Appelqvist-Schmidlechner K, Haataja R (2006) Efficacy of clinical supervision: Influence on job satisfaction, burnout and quality of care. *Journal of Advanced Nursing*, 55(4): 521-535.

Jones A (2003) Some benefits experienced by hospice nurses from group clinical supervision. *European Journal of Cancer Care* 12, 224–232.

Jones 2001 Jones, A. Some experiences of professional practice and beneficial changes derived from clinical supervision by community Macmillan nurses. *European Journal of Cancer Care*, 10: 21-30.

Kelly B, Long A, McKenna H (2001) A survey of community mental health nurses' perceptions of clinical supervision in Northern Ireland. *Journal of Psychiatric and Mental Health Nursing*, 8: 33-44.

Kennedy TJT, Lingard, L, Baker, GR, Kitchen L, Regehr, G (2007) Clinical oversight: Conceptualizing the relationship between supervision and safety. *Society of General Internal Medicine*, 22:1080–1085.

Kenny A & Allenby A (2013) Implementing clinical supervision for Australian rural nurses. *Nurse Education in Practice*, 13: 165-169.

Kilcullen N (2007). An analysis of the experiences of clinical supervision on Registered Nurses undertaking MSc/graduate diploma in renal and urological nursing and on their clinical supervisors. *Journal of Clinical Nursing* 16, 1029–1038.

For peer review only

Knox S, Caperton W, Phelps D, Pruitt N (2014) A qualitative study of supervisees internal representations of supervisors. *Counselling Psychology Quarterly*, 27:4, 334-352.

Koivu A, Saarinen PI, Hyrkas K (2012) Who benefits from clinical supervision and how? The association between clinical supervision and the work-related well-being of female hospital nurses. *Journal of Clinical Nursing*, 21, 2567–2578.

Koivu A, Hyrka, SK, Saarinen PI (2011) Who attends clinical supervision? The uptake of clinical supervision by hospital nurses. *Journal of Nursing Management* 19, 69–79.

Koivu A, Saarinen PI, Hyrkas K (2012) Does clinical supervision promote medical–surgical nurses_ well-being at work? A quasi-experimental 4-year follow-up study *Journal of Nursing Management* 20, 401–413.

Lakeman R & Glasgow C (2009) Introducing peer-group clinical supervision: an action research project. *International Journal of Mental Health Nursing,* 18: 204-210.

Lantz I & Severinsson E (2001) The influence of focus group-oriented supervision on intensive care nurses' reflections on family members' needs. *Intensive and Critical Care Nursing*, 17: 128-137.

Lindgren B & Athlin E (2010) Nurse lecturers' perceptions of what baccalaureate nursing students could gain from clinical group supervision. *Nurse Education Today* 30:360-364.

Lindgren B, Brulin C, Holmlund K, Athlin E (2005) Nursing students' perception of group supervision during clinical training. *Journal of Clinical Nursing* 14, 822–829.

Magnusson A, Lutzen K, Severinsson E (2002) The influence of clinical supervision on ethical issues in home care of people with mental illness in Sweden. *Journal of Nursing Management*, 10: 37-45.

Malin NA (2000) Evaluating clinical supervision in community homes and teams serving adults with learning disabilities. *Journal of Advanced Nursing*, 31(3): 548-557.

O'Connell B, Ockerby CM, Johnson S, Smenda H, Bucknall TK (2011) Team clinical supervision in acute hospital wards: a feasibility study. *Western Journal of Nursing Research*, 35(3): 330-347.

Odling G, Danielson E, Jansson L (2001) Caregivers' descriptions of patients with advanced breast cancer in connection with supervision sessions in a surgical ward. *Cancer Nursing*, 24(1): 28-34.

Olofsson B (2005) Opening up: psychiatric nurses' experiences of participating in reflection groups focusing on the use of coercion. *Journal of Psychiatric and Mental Health Nursing*, 12: 259-67.

Olsson (1998) Systematic clinical supervision of home carers working in the care of demented people who are at home: structure content and effect as experienced by participants. *Journal of Nursing Management,* 6: 239-46.

Palsson M-BE, Hallberg IR, Norbert A (1994) Systematic clinical supervision and its effects for nurses handling demanding care situations: interviews with Swedish district nurses and hospital nurses in cancer care. *Cancer Nursing* 17(5): 385-394.

Pesut DJ & Williams CA (1990) The nature of clinical supervision in psychiatric nursing: a survey of clinical specialists. *Archives of Psychiatric Nursing* 4(3): 188-194.

Saarikoski M, Marrow C, Abreu W, Riklikiene O, O'zbicakc S (2007) Student nurses' experience of supervision and Mentorship in clinical practice: A cross cultural perspective. *Nurse Education in Practice*, 7, 407–415.

For peer review only

Severinsson EI, Hallberg IR (1996) Systematic clinical supervision, working milieu and influence over duties: the psychiatric nurse's viewpoint -a pilot study. *Int J. Nurs. Stud.*, 33(4): 394-406.

Sirola-Karvinen P, Hyrkas K (2008) Administrative clinical supervision as evaluated by the first-line managers in one health care organization district. *Journal of Nursing Management*, 16: 588-600.

Sloan G (1999) Good characteristics of a clinical supervisor: a community mental health nurse perspective. *Journal of Advanced Nursing*, 30(3), 713-722.

Walsh K, Nicholson J, Keough C, Pridham R, Kramer M, Jeffrey J (2003) Development of a group model of clinical supervision to meet the needs of a community mental health nursing team. *International Journal of Nursing Practice*, 9: 33-39.

White E, Butterworth T, Bishop V, Carson J, Jeacock J, Clements A (1998) Clinical supervision: insider reports of a private world. *Journal of Advanced Nursing* 28(1), 185-192.

White E & Winstanley J (2009) A randomised controlled trial of clinical supervision: selected findings from a novel Australian attempt to establish the evidence base for causal relationships with quality of care and patient outcomes, as an informed contribution to mental health nursing practice development. *Journal of Research in Nursing*, 15(2): 151-167.

Williams L & Irvine F (2009) How can the clinical supervisor role be facilitated in nursing: a phenomenological exploration. *Journal of Nursing Management* 17: 474-483.

Secondary studies (Literature reviews):

Brunero S & Stein-Parbury J (2008) The effectiveness of clinical supervision in nursing: an evidenced based literature review. *Australian Journal of Advanced Nursing*, 25(3):86-94.

Butterworth T, Bell L, Jackson C, Majda P (2008) Wicked spell or magic bullet? A review of the clinical supervision literature 2001–2007. *Nurse Education Today*, 28: 264-72.

Dawson M, Phillips B, Leggat S (2013) Clinical Supervision for Allied Health Professionals: A Systematic Review. *J Allied Health,* 42(2):65–73.

Ducat WH, Kumar S (2015) A systematic review of professional supervision experiences and effects for allied health practitioners working in non-metropolitan health care settings. *Journal of Multidisciplinary Healthcare*, 8:397-407.

Farnan JM, Petty LA, Georgitis E, Martin S, Chiu E, Prochaska M, Arora VM (2012) A Systematic Review: The Effect of Clinical Supervision on Patient and Residency Education Outcomes *Academic Medicine*, 87(4): 428-442.

Henshaw A-M, Clarke D, Long AF (2013) Midwives and supervisors of midwives' perceptions of the statutory supervision of midwifery within the United Kingdom: A systematic review. *Midwifery*, 29: 75–85.

Kilminster SM & Jolly BC (2000) Effective supervision in clinical practice settings: a literature review. *Medical Education*, 34:827-840.

Pearce P, Phillips B, Dawson M, Leggat SG (2013) Content of clinical supervision sessions for nurses and allied health professionals: a systematic review. *Clinical Goverance: An international journal*, 18(2): 139-154.

For peer review only

Wheeler S & Richards K (2007) *The impact of clinical supervision on counsellors and therapists, their practice and their clients: a systematic review of the literature* (BACP). British Association for Counselling and Psychotherapy: Lutterworth.

Resilience Training

Mealer M, Conrad D, Evans J, Jooste K, Solyntjes J, Rothbaum B, Moss M (2014) Feasibility and Acceptability of a Resilience Training Program for Intensive Care Unit Nurses, *Am J Crit Care*, 23(6): e97-105.

Peng L, Min L, Zuo X, Miao Y, Chen L, Yu Y, Liu B, Wang T (2014) Application of the Pennsylvania resilience training program on medical students. *Personality and Individual Differences*, 61-62: 47-51.

Sharma V, Bauer B, Prasad K, Sood A, Schroeder D (2012) Self help intervention to decrease stress and increase mindfulness: A pilot trial. *BMC Complement Altern Med*, 12(suppl 1): P253.

Sharma V, Sood A, Prasad K, Loehrer L, Schroeder D, Brent B (2014) Bibliotherapy to decrease stress and anxiety and increase resilience and mindfulness: A pilot trial. *Explore* (NY), 10(4): 248-52 *(reports on the same study as the 2012 paper).*

Sood A, Prasad K, Schroeder D, Prathibha V (2011) Stress management and resilience training among department of medicine faculty: A pilot randomized clinical trial. *J Gen Intern Med*, 26(8): 858-61.

Varker T & Devilly GJ (2012) An analogue trial of inoculation/resilience training for emergency services personnel: Proof of concept. *J Anxiety Disord*, 26(6): 696-701.

Critical Incidence Stress Debriefing

Matthews LR (1998) Effect of Staff Debriefing on Posttraumatic Stress Symptoms After Assaults by Community Housing Residents. *Psychiatric Services*, 49(2): 207-212.

O'Connor J & Jeavons S (2003) Perceived effectiveness of critical incident stress debriefing by Australian nurses. *Australian Journal of Advanced Nursing*, 20(4): 22-29.

Peer Supported Story Telling

Macpherson CF (2008) Peer-supported storytelling for grieving pediatric oncology nurses *J Pediatr Oncol Nurs* 25(3): 148-163.

Reflective Practice Groups

Abbas K & Pierce CM (2013) Time to reflect: Reflective practice groups for consultants and registrars on a neonatal and paediatric intensive care unit (N/PICU). *24th Annual Meeting of the European Society of Paediatric and Neonatal Intensive Care, ESPNIC 2013 Rotterdam Netherlands,* 39, S51-S52.

Binks C, Jones FW & Knight K (2013) Facilitating reflective practice groups in clinical psychology training: A phenomenological study. *Reflective Practice,* 14: 305-318.

Boyd EM & Fayles AW (1983) Reflective learning: Key to learning from experience. *Journal of Humanistic Psychology*, 23: 99–117.

Dawber C (2013) Reflective practice groups for nurses: A consultation liaison psychiatry nursing initiative: Part 2 - the evaluation. *International Journal of Mental Health Nursing,* 22: 241-248.

For peer review only

Gould B & Masters H (2004). Learning to make sense: the use of critical incident analysis in facilitated reflective groups of mental health student nurses. *Learning in Health & Social Care,* 3: 53-3.

Hansom J & Butler M (2003) Sharing reflections in midwifery practice. *British Journal of Midwifery,* 11: 34-37.

Hayes J (1998) Learning from practice: developing the reflective skills of forensic psychiatric nurses. *Psychiatric Care,* 5: 30-33.

Heneghan C, Wright J, & Watson G (2014) Clinical psychologists' experiences of reflective staff groups in inpatient psychiatric settings: A mixed methods study. *Clinical Psychology & Psychotherapy,* 21:324-340.

Hong LP & Chew L (2008) Reflective practice from the perspectives of the bachelor of nursing students in International Medical University (IMU). *Singapore Nursing Journal,* 35:5.

Johnston J & Paley G (2013) Mirror mirror on the ward: Who is the unfairest of them all? Reflections on reflective practice groups in acute psychiatric settings. *Psychoanalytic Psychotherapy,* 27:170-186.

Kiff J, Holmes G, & Cushway D (2010) Personal awareness / development groups and clinical training. *Clinical Psychology Forum*, 207: 30–34.

Knight K, Sperlinger D, & Maltby M (2010) Exploring the personal and professional impact of reflective practice groups: A survey of 18 cohorts from a UK clinical psychology training course. *Clinical Psychology & Psychotherapy,* 17: 427-437.

Kung JW, Eisenberg RL, & Slanetz PJ (2012) Reflective Practice as a Tool to Teach Digital Professionalism. *Academic Radiology,* 19: 1408-1414.

Manning A, Cronin P, Monaghan A & Rawlings-Anderson K (2009) Supporting students in practice: an exploration of reflective groups as a means of support. *Nurse Education in Practice,* 9: 176-183.

Parish C, Bradley L, & Franks V (1997) Managing the stress of caring in ITU: a reflective practice group. *British journal of nursing (Mark Allen Publishing),* 6: 1192-1196.

Platzer H, Blake D, & Ashford D (2000) An evaluation of process and outcomes from learning through reflective practice groups on a post-registration nursing course. *Journal of advanced nursing,* 31: 689-695.

Schon D (1983) *The Reflective Practitioner: How Professionals Think in Action*. New York, NY: Basic Books.

Psychosocial Intervention Training

Redhead K; Bradshaw T; Braynion P; Doyle M (2011) An evaluation of the outcomes of psychosocial intervention training for qualified and unqualified nursing staff working in a low-secure mental health unit. *Journal of Psychiatric & Mental Health Nursing*, 18(1): 59-66.

Ewers P; Bradshaw T; McGovern J; Ewers B (2002) Does training in psychosocial interventions reduce burnout rates in forensic nurses? *Journal of Advanced Nursing* 37(5): 470-476.

Doyle M, Kelly D, Clarke S, Braynion P(2007) Burnout: the impact of psychosocial interventions training. *Mental Health Practice* 10: 16–19.

For peer review only

Mindfulness Based Stress Reduction

Bazarko D, Cate RA, Azocar F, Kreitzer MJ (2013) The impact of an innovative mindfulness-based stress reduction program on the health and well-being of nurses employed in a corporate setting. *Journal of Workplace Behavioral Health*, 28(2): 107-133.

Beddoe AE. Murphy SO (2004) Does mindfulness decrease stress and foster empathy among nursing students? *J.Nurs.Educ*.43(7): 305-312.

Brady S, O'Connor N, Burgermeister D, Hanson P (2012) The Impact of Mindfulness Meditation in Promoting a Culture of Safety on an Acute Psychiatric Unit. *Perspect.Psychiatr.Care*, 48(3): 129-137.

Cohen-Katz J, Wiley S, Capuano T, Baker DM, Deitrick L, Shapiro S (2005) The effects of mindfulness-based stress reduction on nurse stress and burnout: a qualitative and quantitative study, part III. *Holist.Nurs.Pract*, 19(2): 78-86.

de Vibe M, Solhaug I, Tyssen R, Friborg O, Rosenvinge JH, Sorlie T, Bjorndal A (2013) Mindfulness training for stress management: a randomised controlled study of medical and psychology students. *BMC medical education*, 13: 107.

Foureur M, Besley K, Burton G, Yu N, Crisp J (2013) Enhancing the resilience of nurses and midwives: Pilot of a mindfulness-based program for increased health, sense of coherence and decreased depression, anxiety and stress. *Contemporary Nurse*, 45(1): 114-125.

Geary C, Rosenthal S, (2011) Sustained impact of MBSR on stress, well-being, and daily spiritual experiences for 1 year in academic health care employees. *The Journal of Alternative and Complementary Medicine*, 17(1): 939-944.

Goodman MJ, Schorling JB (2012) A mindfulness course decreases burnout and improves well-being among healthcare providers. *Int.J.Psychiatry Med.*, 43(2): 119-128.

Hallman I, O'Connor, N, Hasenau, S, Brady S (2014) Improving the Culture of Safety on a High-Acuity Inpatient Child/Adolescent Psychiatric Unit by Mindfulness-Based Stress Reduction Training of Staff. *J.Child Adolesc.Psychiatr.Nurs*. 27(4): 183-189.

Irving JA, ParkSaltzman J, Fitzpatrick M, Dobkin PL, Chen A, Hutchinson T (2014) Experiences of health care professionals enrolled in mindfulness-based medical practice: A grounded theory model. *Mindfulness*, 5(1): 60-71.

Mackenzie CS, Poulin PA, Seidman-Carlson R (2006) A brief mindfulness-based stress reduction intervention for nurses and nurse aides. *Applied Nursing Research*, 19(2): 105-109.

Martin-Asuero A, Garcia-Banda G (2010) The mindfulness-based stress reduction program (MBSR) reduces stress-related psychological distress in healthcare professionals. *Spanish Journal of Psychology*, 13(2): 897-905.

Marx R, Strauss C, Williamson C, Karunavira T (2014) The eye of the storm: A feasibility study of an adapted mindfulness-based cognitive therapy (MBCT) group intervention to manage NHS staff stress. *The Cognitive Behaviour Therapist*, 7 Dec, Art e18-17.

Moody K; Kramer D, Santizo RO, Magro L, Wyshogrod D, Ambrosio J, Castillo C, Lieberman R, Stein J (2013) Helping the helpers: Mindfulness training for burnout in pediatric oncology-a pilot program. *Journal of Pediatric Oncology Nursing,* 30(5): 275-284.

For peer review only

Poulin PA, Mackenzie CS, Soloway G, Karayolas E (2008) Mindfulness training as an evidenced-based approach to reducing stress and promoting well-being among human services professionals. *International Journal of Health Promotion and Education*, 46(2): 72-80.

Rosenzweig S, Reibel DK, Greeson JM, Brainard GC, Hojat M (2003) Mindfulness-based stress reduction lowers psychological distress in medical students. *Teach.Learn.Med.*, 15(2): 88-92.

Shapiro SL, Astin JA, Bishop SR, Cordova M (2005) Mindfulness-Based Stress Reduction for Health Care Professionals: Results From a Randomized Trial. *International Journal of Stress Management*, 12(2): 164-176.

Shapiro SL, Schwartz GE, Bonner G (1998) Effects of mindfulness-based stress reduction on medical and premedical students. *J.Behav.Med.*,21(6): 581-599.

For peer review only

Supplementary File 3: Evidence base (in healthcare professionals) for Schwartz Rounds® and comparative interventions

Intervention	Healthcare settings	Number/type of papers included	Intervention fidelity between studies	Key Findings to:<br>Self/ Others/ Organisation	Overall strength of the evidence base*
**Schwartz Center Rounds®**	Hospitals, hospices, and educational settings (e.g. medical schools) – open to all staff of all grades (clinical and non-clinical).	10 papers (8 studies):<br>- 2 quantitative<br>- 1 qualitative<br>- 5 mixed methods	Yes as Rounds are licenced and have to be run according to a format subsequent to obtaining a licence.	**Self**<br>_Wellbeing:_ reduced stress, emotional labour, and isolation at work<br>_Coping:_ improved ability to cope with emotional difficulties at work; confidence in handling sensitive issues<br>_Self-reflection_: increased self-reflection/validation of experiences<br>_Knowledge_: students report increased knowledge/understanding about emotional side of providing patient care.<br><br>**Others**<br>_Patients_: increased compassion and empathy, increased interaction.<br>_Colleagues_: improved teamwork, communication, compassion/empathy<br><br>**Organisation**<br>_Access_: improved access to specific services (e.g palliative care)<br>_Culture change_: having dialogue that doesn't happen elsewhere; reinforced shared values and support strategic vision; reduced hierarchy	Quantitative: low/moderate due to cross-sectional design, non-validated measures, and lack of control (non-attender) data.<br>Qualitative: moderate due to limited reporting of theoretical underpinnings and strategies to improve rigour (e.g. deviant case analysis; reflexivity)<br>Mixed methods: Low

For peer review only

Intervention	Healthcare settings	Number/type of papers included	Intervention fidelity between studies	Key Findings to: Self/ Others/ Organisation	Overall strength of the evidence base*
**Action Learning Sets**	Pre/post registration education/training; primary care (e.g. online networks for GPs); acute and mental health services; palliative and continuing care; care homes and prisons.	14 papers:<br>- 6 quantitative<br>- 4 qualitative<br>4 mixed methods	Most hybrid interventions where ALS only one component. Group sizes between 4-8 members. Evaluation period 3months to a year, and 2- 6 sessions.	**Self**<br>*Empowerment:* foster greater psychological empowerment; sense of self-efficacy, self-esteem and confidence<br>*Awareness:* offer opportunity to explore self<br><br>**Others**<br>*Colleagues:* improved understanding and knowledge of colleagues; offers opportunity to share experiences and give/receive peer support<br><br>**Organisation**<br>*Workforce*: opportunities for mentoring and advice; satisfaction with the intervention being accessible and inclusive; possible limitations include having conflicting commitments and lack of group cohesiveness	Quantitative: Low due to lack of validated measures, and small sample sizes that were underpowered.<br><br>Qualitative: Moderate to high quality: some well-designed studies but others lacked detail on analytical strategy, limited transparency of findings (e.g. deviant cases not discussed, quotes not fitting themes and/or author interpretation)<br><br>Mixed methods: Low
**After Action Reviews (AAR)**	Acute settings	2 papers:<br>2 quantitative	Variability evident: 2 year evaluation of post-fall huddles vs. cross-sectional evaluation of one-off	**Self**<br>*Empowerment:* improved confidence in dealing with difficult situations<br><br>**Others**<br>*Patients:* improved communication and listening skills	Quantitative: Low to moderate – mostly non-experimental designs with convenience samples and non-validated (study-specific)

For peer review only

Intervention	Healthcare settings	Number/type of papers included	Intervention fidelity between studies	Key Findings to:<br>Self/ Others/ Organisation	Overall strength of the evidence base*
			training in AAR	**Organisation**<br>*Practice:* reduced task and coordination errors; increased uptake of post-fall huddles; positive impact on patient care	outcome measures; poor survey response.
**Balint groups**	Primary care (predominantly GPs), medical schools, acute settings	26 papers:<br>- 12 quantitative<br>- 6 qualitative<br>- 8 mixed methods	Principles applied consistently but some variations or hybrid-models (e.g. Balint-inspired roundtable groups)	**Self**<br>*Awareness:* raised personal awareness in doctors; helped medical students build a professional identity as doctors<br>*Resilience:* reduced stress; helped doctors and students resolve challenging situations in practice<br>*Job satisfaction*: improved job satisfaction in doctors<br><br>**Others**<br>*Patients:* foster positive attitudes towards 'difficult' patients; improve students' ability to relate with patients; improve patient-centredness<br>*Colleagues:* promote teamwork<br><br>**Organisation**<br>*Practice:* reduced unnecessary prescriptions; increased uptake of psychosocial support; higher patient satisfaction	Quantitative: Low – mostly observational studies that lack randomisation, use non-validated measurement tools, and inadequately control confounding variables.<br><br>Qualitative: Moderate – mostly reflective case studies, with loose adherence to qualitative designs and limited description of approaches to rigour.<br><br>Mixed methods: Low

For peer review only

Intervention	Healthcare settings	Number/type of papers included	Intervention fidelity between studies	Key Findings to: Self/ Others/ Organisation	Overall strength of the evidence base*
**Caregiver Support Program**	Mental health /learning disability homes	3 papers:<br>- 3 quantitative (all the same study)	No variability – has only been used and evaluated in one study.	**Self**<br>Empowerment: measured ability to cope with common work problems and ability to influence decision making (non-significant improvement);<br>Wellbeing: psychological wellbeing (non-significant improvement)<br><br>**Others**<br>Colleagues: greater supervisor support, less undermining, greater praise and feedback<br><br>**Organisation**<br>Workforce: No change in outcomes for managers (only direct-care staff); "train the trainers" element (to translate intervention to workplace) did not work	Quantitative: Moderate to low – the main limitations concern the sample (low response rates to surveys), method of analysis (did not use all the data, could have included people responding at only one time point) and measures (use of measures that were not well validated)
**Clinical and Restorative Supervision**	Nursing education leadership, and nursing generally (including mental health, paediatrics, addiction, elderly and dementia care, oncology, hospice nursing), and in	64 papers:<br>9 Secondary studies (literature reviews)<br>55 Primary studies: - 22 quantitative<br>27 qualitative<br>- 6 mixed methods	Wide variability in every aspect. Duration ranged from 1 mth to 5 yrs (median 12 mths). Session length ranged from 1- 3hrs	**Self**<br>Awareness: improved knowledge and insights, professional awareness and development<br>Empowerment: improved communication skills, job resources, professional efficacy and capacity for reflection<br>Wellbeing: reduced psychological distress; improved vitality; reduced stress; reduced burnout (lower emotional	Quantitative: Mostly Low/Moderate. Most are cross-sectional studies, with a few pre-post (mostly without control groups) and short-term follow-up. Only a few RCTs have been conducted and these

For peer review only

Intervention	Healthcare settings	Number/type of papers included	Intervention fidelity between studies	Key Findings to:<br>Self/ Others/ Organisation	Overall strength of the evidence base*
	emergency departments.		(median 1.5hrs), and frequency from weekly to monthly. Most were group supervision, ranging from 2 to 12 supervisees. Treatment fidelity was rarely referred to.	exhaustion and depersonalisation); improved sense of security, belonging and encouragement<br>*Resilience:* improved rational coping<br>*Job stress/satisfaction*: reduced job stress; improved job satisfaction<br><br>**Others**<br>Patients: better knowledge of patients' suffering and how to take responsibility; improved individualised documentation in patient notes;<br>Colleagues: foster solidarity, sharing and reflecting<br><br>***Organisation***<br>*Practice:* Reduced positive and total symptoms (in patients with psychosis) reported by student nurses<br>*Workforce:* staff retention<br><br>Continuity and quality of supervision are key moderators of outcomes.	are moderate-high quality. Inadequate sample sizes and unvalidated/ unreliable measures in most (mostly self-report of supervisor/supervisee rather than impact on patient/care).<br><br>Qualitative: Low/Moderate. Majority lack conceptual/theoretical bases; have inappropriate sampling and lack of transparency in methods/analysis.<br><br>Mixed methods: Low
**Critical Incidence Stress Debriefing**	Acute and community settings.	2 papers:<br>-2 quantitative	Fairly consistent approach to CISD following the	**Self**<br>*Wellbeing:* no significant impact on stress reduction, but significantly lower PTSD scores; feeling part of a group and realising they were not alone	Quantitative: Moderate –weaknesses or limited reporting of group allocation, sampling approach,

For peer review only

Intervention	Healthcare settings	Number/type of papers included	Intervention fidelity between studies	Key Findings to: Self/ Others/ Organisation	Overall strength of the evidence base*
			Mitchell model (1983). Details of the content of the intervention lacking. Consistency in approach between group facilitators questionable.	**Others**<br>*Colleagues:* opportunity for sharing experiences, and learning from others<br><br>**Organisation**<br>*Workforce:* potentially negative impacts included increased intrusive thoughts	consideration of confounding variables, and psychometric values of instruments used.
**Mindfulness-based Stress Reduction**	Acute and community settings, with doctors and nurses (qualified and in-training) and a range of allied health professions.	17 papers:<br>-15 quantitative<br>-1 qualitative<br>-1 mixed method	Mostly informed by Kabat-Zinn (1990).<br>One study combined face-to-face and telephone delivery of sessions; and five studies reported on a shortened version.<br>Content of sessions was similar across	**Self**<br>*Wellbeing:* improved relaxation and mood. Some mixed results in relation to burnout, stress, anxiety, distress, and satisfaction with life<br>*Resilience:* improved self-compassion, self-care, acceptance and coping skills<br>**Others**<br>*Colleagues:* opportunity for peer support	Quantitative: Moderate due to non-probability sampling, bias from confounding and lack of power calculations.<br>Qualitative: Moderate due to limited reporting of approaches to enhancing rigour (e.g. member checking, deviant cases, reflexivity, data saturation).<br>Mixed Methods: Low

For peer review only

Intervention	Healthcare settings	Number/type of papers included	Intervention fidelity between studies	Key Findings to:<br>Self/ Others/ Organisation	Overall strength of the evidence base*
			studies, but many lacked detail to judge fidelity.		
**Peer-supported storytelling**	Paediatric 17 papers<br>-15 quantitative<br>-1 qualitative<br>-1 mixed method<br>nurses who had experienced grief	1 paper:<br>- mixed methods	Only one study consisted of 3 self-selected dyads (6 nurses). Each dyad met biweekly for 2 months for brief informal storytelling sessions (each member taking a turn telling a story and listening at each session, mean length 17mins).	**Self**<br>Wellbeing: positive impact on grief<br>Resilience: positive impact on meaning-making, making sense of, and identifying benefit in, their experiences<br><br>**Others**<br>Colleagues: opportunity to receive and provide support during sessions<br><br>**Organisation**<br>Workforce: significant positive correlation between number of 'special' patient deaths during career and impact of sessions on grief	Quantitative: Low due to small (n=6), self-selected sample and no comparison group<br><br>Qualitative: Moderate due to descriptive approach to analysis, lacking in theoretical saturation<br><br>Mixed methods: Low
**Psychosocial intervention training**	Mental health settings in the UK	3 papers:<br>-3 quantitative	Consistent programme; delivered weekly over 4-5 months.	**Self**<br>Wellbeing: improved burnout (emotional exhaustion, depersonalisation and personal achievement)	Quantitative: Moderate –all 3 studies were quasi-experimental, utilising non-probability sampling,

Intervention	Healthcare settings	Number/type of papers included	Intervention fidelity between studies	Key Findings to: Self/ Others/ Organisation	Overall strength of the evidence base*
			Variable duration between 4 to 8 months.	_Empowerment:_ improved knowledge of, and attitude towards, mental illness and psychosocial approaches	but lacking a power calculation and not clearly accounting for confounding variables.
**Reflective Practice Groups**	Clinical psychology, psychotherapy, nursing, medicine, midwifery and radiology.	8 papers:<br>-3 quantitative<br>-3 qualitative<br>-2 mixed methods	All described as facilitated groups that explore practice related issues. Can last between 45-90 minutes; held weekly, fortnightly or monthly; group sizes varied from six to 20 attendees, with one or two facilitators.	**Self**<br>_Awareness:_ improved self-awareness and clinical insight<br>_Empowerment:_ increased confidence and capacity for reflection; better understanding of psychological ideas<br>_Wellbeing:_ increased ability to cope with stress<br><br>**Others**<br>_Colleagues:_ opportunity for peer support, sharing experience and learning; improved communication skills<br>_Patients:_ positive impact on empathy<br><br>**Organisation**<br>_Practice:_ perceived improvements in the quality of care provided	Quantitative: Low – mostly weakened by the absence of probability sampling, lack of validated tools, missing baseline measurements and inadequate control for confounding variables.<br><br>Qualitative: Low – mostly weakened by absence of an overall design and inadequate attention to data saturation, sampling and researcher reflexivity.<br><br>Mixed methods: Low
**Resilience Training**	Medical students, intensive care unit nurses, general medicine,	6 papers:<br>- 6 quantitative (reporting on five studies)	Two studies (three papers) evaluated resilience	**Self**<br>_Wellbeing:_ reduced depression, PTSD, stress and anxiety	Quantitative: Moderate –mostly pilot studies to determine the feasibility,

For peer review only

Intervention	Healthcare settings	Number/type of papers included	Intervention fidelity between studies	Key Findings to: Self/ Others/ Organisation	Overall strength of the evidence base*
	emergency service personnel.		training alone. The remainder combined resilience training with other interventions. Interventions varied from 10-12 weeks of regular training, to 40min one-off.	*Resilience:* improved cognitive appraisal, control of stress; decreased negative emotion and expression inhibition	acceptability or prove the concept of the intervention. Limitations included only comparing within groups (despite having control group), ambiguous details regarding sampling and/or lacking control group.

References:

Kabat-Zinn, J. (1990). *Full catastrophe living: Using the wisdom of your body and mind to face stress, pain and illness.* New York: Delacorte

Mitchell, J. T. (1983). When disaster strikes: The critical incident stress debriefing process. Journal of Emergency Medical Services, **8**(1):36-9.

For peer review only

PRISMA 2009 Checklist

Section/topic	#	Checklist item	Reported on page #
**TITLE**			
Title	1	Identify the report as a systematic review, meta-analysis, or both.	1
**ABSTRACT**			
Structured summary	2	Provide a structured summary including, as applicable: background; objectives; data sources; study eligibility criteria, participants, and interventions; study appraisal and synthesis methods; results; limitations; conclusions and implications of key findings; systematic review registration number.	2-3
**INTRODUCTION**			
Rationale	3	Describe the rationale for the review in the context of what is already known.	4-6
Objectives	4	Provide an explicit statement of questions being addressed with reference to participants, interventions, comparisons, outcomes, and study design (PICOS).	6
**METHODS**			
Protocol and registration	5	Indicate if a review protocol exists, if and where it can be accessed (e.g., Web address), and, if available, provide registration information including registration number.	n/a
Eligibility criteria	6	Specify study characteristics (e.g., PICOS, length of follow-up) and report characteristics (e.g., years considered, language, publication status) used as criteria for eligibility, giving rationale.	6-7
Information sources	7	Describe all information sources (e.g., databases with dates of coverage, contact with study authors to identify additional studies) in the search and date last searched.	6
Search	8	Present full electronic search strategy for at least one database, including any limits used, such that it could be repeated.	6
Study selection	9	State the process for selecting studies (i.e., screening, eligibility, included in systematic review, and, if applicable, included in the meta-analysis).	7
Data collection process	10	Describe method of data extraction from reports (e.g., piloted forms, independently, in duplicate) and any processes for obtaining and confirming data from investigators.	7
Data items	11	List and define all variables for which data were sought (e.g., PICOS, funding sources) and any assumptions and simplifications made.	n/a
Risk of bias in individual studies	12	Describe methods used for assessing risk of bias of individual studies (including specification of whether this was done at the study or outcome level), and how this information is to be used in any data synthesis.	7
Summary measures	13	State the principal summary measures (e.g., risk ratio, difference in means).	8
Synthesis of results	14	Describe the methods of handling data and combining results of studies, if done, including measures of consistency (e.g., I^2) for each meta-analysis.	n/a

For peer review only - http://bmjopen.bmj.com/site/about/guidelines.xhtml

PRISMA 2009 Checklist

Section/topic	#	Checklist item	Reported on page #
Risk of bias across studies	15	Specify any assessment of risk of bias that may affect the cumulative evidence (e.g., publication bias, selective reporting within studies).	n/a
Additional analyses	16	Describe methods of additional analyses (e.g., sensitivity or subgroup analyses, meta-regression), if done, indicating which were pre-specified.	n/a
**RESULTS**			
Study selection	17	Give numbers of studies screened, assessed for eligibility, and included in the review, with reasons for exclusions at each stage, ideally with a flow diagram.	11, 25 (Table 1)
Study characteristics	18	For each study, present characteristics for which data were extracted (e.g., study size, PICOS, follow-up period) and provide the citations.	28-32 (Table 2)
Risk of bias within studies	19	Present data on risk of bias of each study and, if available, any outcome level assessment (see item 12).	28-32 (Table 2)
Results of individual studies	20	For all outcomes considered (benefits or harms), present, for each study: (a) simple summary data for each intervention group (b) effect estimates and confidence intervals, ideally with a forest plot.	28-32 (Table 2)
Synthesis of results	21	Present results of each meta-analysis done, including confidence intervals and measures of consistency.	Narrative synthesis 11-12
Risk of bias across studies	22	Present results of any assessment of risk of bias across studies (see Item 15).	11-12
Additional analysis	23	Give results of additional analyses, if done (e.g., sensitivity or subgroup analyses, meta-regression [see Item 16]).	n/a
**DISCUSSION**			
Summary of evidence	24	Summarize the main findings including the strength of evidence for each main outcome; consider their relevance to key groups (e.g., healthcare providers, users, and policy makers).	15-16
Limitations	25	Discuss limitations at study and outcome level (e.g., risk of bias), and at review-level (e.g., incomplete retrieval of identified research, reporting bias).	18
Conclusions	26	Provide a general interpretation of the results in the context of other evidence, and implications for future research.	18
**FUNDING**			
Funding	27	Describe sources of funding for the systematic review and other support (e.g., supply of data); role of funders for the systematic review.	19

From: Moher D, Liberati A, Tetzlaff J, Altman DG, The PRISMA Group (2009). Preferred Reporting Items for Systematic Reviews and Meta-Analyses: The PRISMA Statement. PLoS Med 6(7): e1000097.

For peer review only - http://bmjopen.bmj.com/site/about/guidelines.xhtml

PRISMA 2009 Checklist

doi:10.1371/journal.pmed1000097 For more information, visit: **www.prisma-statement.org**. Page 2 of 2

For peer review only
